# Association between type I interferon pathway activation and clinical outcomes in rheumatic and musculoskeletal diseases: a systematic literature review informing EULAR points to consider

**DOI:** 10.1136/rmdopen-2022-002864

**Published:** 2023-03-07

**Authors:** Javier Rodríguez-Carrio, Agata Burska, P G Conaghan, Willem A Dik, Robert Biesen, Maija-Leena Eloranta, Giulio Cavalli, Marianne Visser, Dimitrios T Boumpas, George Bertsias, Marie Wahren-Herlenius, Jan Rehwinkel, Marie-Louise Frémond, Mary K Crow, Lars Ronnblom, Ed Vital, Marjan Versnel

**Affiliations:** 1 Area of Immunology, University of Oviedo, Instituto de Investigación Sanitaria del Principado de Asturias (ISPA), Asturias, Spain; 2 Leeds Institute of Rheumatic and Musculoskeletal Medicine, University of Leeds & NIHR Leeds Biomedical Research Centre, Leeds, UK; 3 Laboratory Medical Immunology, department of Immunology, Erasmus MC University Medical Center Rotterdam, The Netherlands; 4 Department of Rheumatology, Charité University Medicine Berlin, Berlin, Germany; 5 Department of Medical Sciences, Rheumatology, Uppsala University, Uppsala, Sweden; 6 Unit of Immunology, Rheumatology, Allergy and Rare Diseases, Vita-Salute San Raffaele University, Milan, Italy; 7 EULAR, PARE Patient Research Partners, Amsterdam, The Netherlands; 8 Department of Internal Medicine, University of Crete, Medical School, Heraklion, Greece; 9 Department of Rheumatology-Clinical Immunology, University of Crete, Medical School, Heraklion, Greece; 10 Karolinska Institutet, Division of Rheumatology, Stockholm, Sweden; 11 Broegelmann Research Laboratory, Department of Clinical Science, University of Bergen, Norway; 12 Medical Research Council Human Immunology Unit, Medical Research Council Weatherall Institute of Molecular Medicine, Radcliffe Department of Medicine, University of Oxford, UK; 13 Université de Paris Cité, Hôpital Necker-Enfants Malades, Immuno-Hématologie et Rhumatologie pédiatriques, Paris, France; 14 Hospital for Special Surgery, Weill Cornell Medical College, Mary Kirkland Center for Lupus Research, New York, USA; 15 Department of Immunology, Erasmus MC University Medical Center Rotterdam, The Netherlands

**Keywords:** cytokines, arthritis, rheumatoid, autoimmunity, immune system diseases, lupus erythematosus, systemic

## Abstract

**Background:**

Type I interferons (IFN-I) contribute to a broad range of rheumatic and musculoskeletal diseases (RMDs). Compelling evidence suggests that the measurement of IFN-I pathway activation may have clinical value. Although several IFN-I pathway assays have been proposed, the exact clinical applications are unclear. We summarise the evidence on the potential clinical utility of assays measuring IFN-I pathway activation.

**Methods:**

A systematic literature review was conducted across three databases to evaluate the use of IFN-I assays in diagnosis and monitor disease activity, prognosis, response to treatment and responsiveness to change in several RMDs.

**Results:**

Of 366 screened, 276 studies were selected that reported the use of assays reflecting IFN-I pathway activation for disease diagnosis (n=188), assessment of disease activity (n=122), prognosis (n=20), response to treatment (n=23) and assay responsiveness (n=59). Immunoassays, quantitative PCR (qPCR) and microarrays were reported most frequently, while systemic lupus erythematosus (SLE), rheumatoid arthritis, myositis, systemic sclerosis and primary Sjögren’s syndrome were the most studied RMDs. The literature demonstrated significant heterogeneity in techniques, analytical conditions, risk of bias and application in diseases. Inadequate study designs and technical heterogeneity were the main limitations. IFN-I pathway activation was associated with disease activity and flare occurrence in SLE, but their incremental value was uncertain. IFN-I pathway activation may predict response to IFN-I targeting therapies and may predict response to different treatments.

**Conclusions:**

Evidence indicates potential clinical value of assays measuring IFN-I pathway activation in several RMDs, but assay harmonisation and clinical validation are urged. This review informs the EULAR points to consider for the measurement and reporting of IFN-I pathway assays.

**WHAT IS ALREADY KNOWN ON THIS TOPIC**

- The type I interferon (IFN-I) activation pathway has been related to a number of rheumatic and musculoskeletal diseases (RMDs), but the optimal assays for detection and clinical applications are unclear.

**WHAT THIS STUDY ADDS**

- This systematic review revealed significant heterogeneity in IFN-I evidence in RMDs in terms of clinical applications (diagnosis, measurement of disease activity, prognosis, prediction of response and assay responsiveness) that may account for the lack of transition of IFN-I assays into clinical practice.
- Immunoassays, quantitative PCR and microarrays were reported most frequently, while systemic lupus erythematosus (SLE), rheumatoid arthritis, myositis, systemic sclerosis and primary Sjögren’s syndrome were the most studied RMDs.
- IFN-I pathway activation was associated with disease activity and flare occurrence in SLE, although in most contexts the added value to existing instruments in clinical care needs to be determined.
- IFN-I assays can predict response to IFN-I targeting drugs and may also predict response to other classes of therapies.

**HOW THIS STUDY MIGHT AFFECT RESEARCH, PRACTICE OR POLICY**

- Assays measuring IFN-I pathway activation show considerable promise to improve the management of RMDs, guiding clinical decisions along the whole therapeutic process. We demonstrate where harmonisation of assay methods, improved study designs and clinical validation studies are required to realise this potential.
- This review informs the EULAR points to consider for the measurement, reporting and application of IFN-I pathway activation assays in clinical and research practice, which may also be of interest beyond the field of rheumatology.

## Introduction

Type I interferons (IFN-I) are cytokines with well-known antiviral and immunomodulatory activities, involved in both innate and adaptive responses.^1^ Aberrant IFN-I production, signalling or altered regulation has been observed in a wide range of rheumatic and musculoskeletal diseases (RMDs).^2–4^ Moreover, preclinical research has provided mechanistic insights for IFN-I pathway activation in RMD pathogenesis. Furthermore, drugs targeting different components of the IFN-I pathway have been licensed for systemic lupus erythematosus (SLE) and thereby support the pathogenic role of IFN-I in autoimmunity.^4^

The identification of biomarkers to improve clinical management is an important area in the field of rheumatology. However, the contribution of IFN-I pathway activation assays as biomarkers in this setting remains unclear. In this regard, it is important to note that different assays, which measure different molecules reflecting distinct aspects and components of the IFN-I pathway activation, or different IFN-I family members have been proposed. Several IFN-I pathway activation assays have been reported to correlate with clinical features across RMDs. As such, there is a substantial body of evidence suggesting a role for IFN-I in disease diagnosis, prognosis, monitoring and prediction of response. Despite this potential, the translation of assays evaluating IFN-I pathway activation into clinical practice has been rare.

Under these circumstances, and due to the relevance for RMD management, a EULAR Task Force was convened to address this unmet need. The aim of the present study was to perform a comprehensive systematic literature review (SLR) to appraise the existing literature about the potential clinical relevance of IFN-I pathway assays in RMDs to develop points to consider.

## Material and methods

This SLR was performed in accordance with the EULAR standardised operating procedures for EULAR-endorsed projects.^5^ A multidisciplinary task force of 17 members (from 8 EULAR countries and the USA), with different backgrounds including rheumatologists, medical immunologists, virologists, translational researchers and experts in interferonopathies was formed. The task force outlined the scope of the literature search and identified six topics about the use and reporting of IFN-I pathway assays in RMDs. The first research question was focused on assay methodology (properties and classification) and it was published as a separate SLR.^6^ The remaining five research questions concerned the association with clinical outcomes and were formulated under the Population, Intervention, Comparator, Outcome (PICO) framework (online supplemental text 1) for the purpose of this SLR.

10.1136/rmdopen-2022-002864.supp1 Supplementary data

### Search strategy

A search strategy (online supplemental texts 2–4) was developed based on the predefined PICO and implemented in Ovid Medline, Embase and Web of Science on 31 October 2019, with the support of an experienced librarian. Titles and abstracts, followed by full-text screening was performed by two reviewers (AB and JR-C). The agreement between reviewers was high (>95%), and discrepancies were resolved by discussion or consultation with the convenor (EV).

### Inclusion and exclusion criteria

Papers were included in the SLR by a two-step process. First, articles were selected if they report an assay to measure IFN-I pathway activation, according to predefined inclusion and exclusion criteria (online supplemental text 5). Next, these papers were further screened for specific eligibility criteria related to the associations with clinical outcomes (diagnosis, disease activity, prognosis, response to treatment and assay responsiveness/change over time) in RMDs (online supplementary text 6).

### Data extraction and synthesis

Data from the included studies were extracted using a standardised template. The risk of bias for each study was assessed using validated tools according to the study design (online supplemental text 7) and classified as low, unclear or high. Data were organised by RMD and method used (online supplemental text 8), based on the classification proposed in the accompanying SLR.^6^ Due to the broad heterogeneity, results were presented in the form of a narrative summary.

## Results

**Figure 1:**
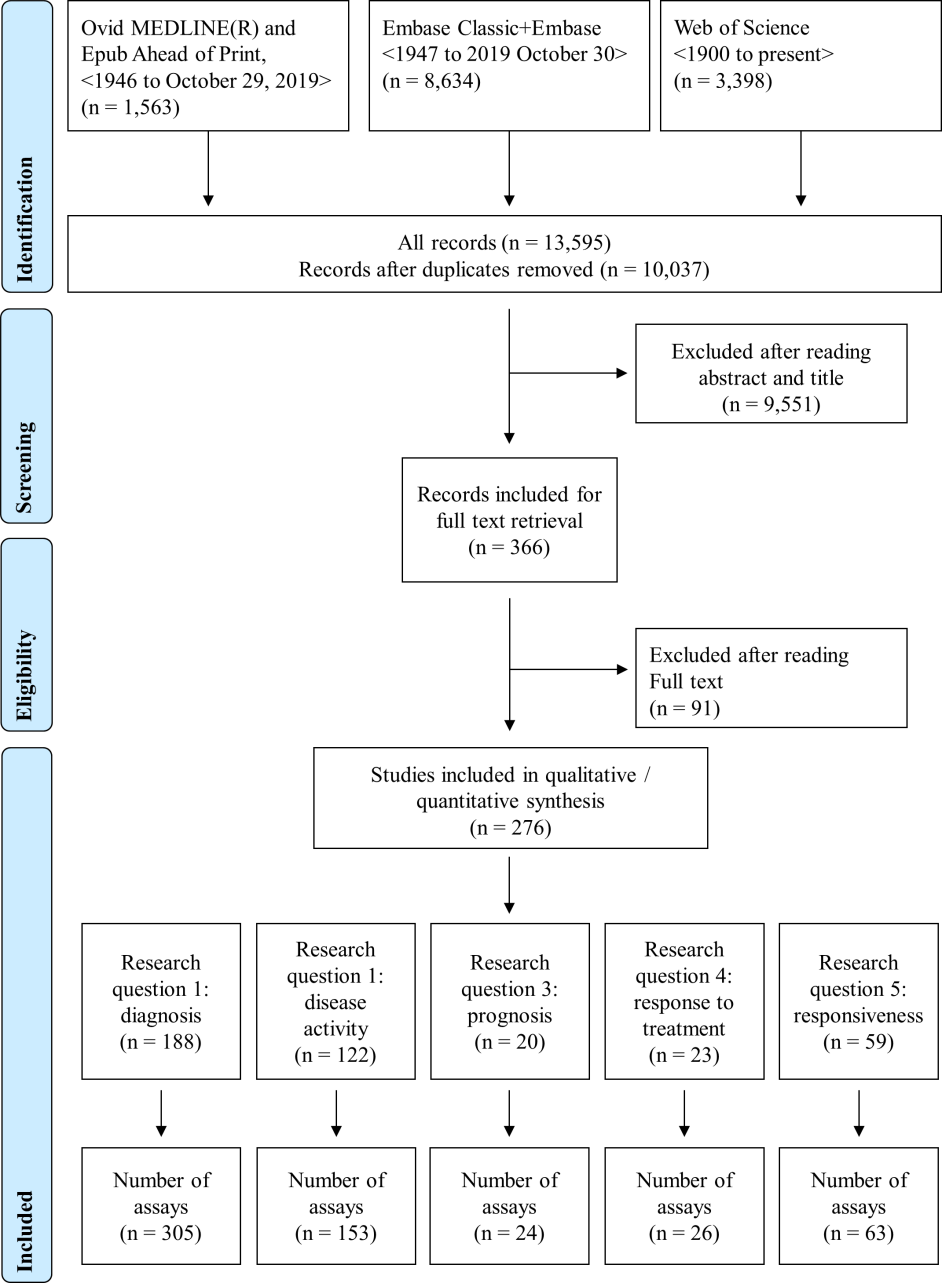
PRISMA (Preferred Reporting Items for Systematic Reviews and Meta-Analyses) flow diagram. This flow chart shows the study selection and the search strategies. Since the different research questions proposed had different eligibility criteria, the number of excluded and included articles varies.

The search strategy yielded a total of 366 full-texts, of which 276 papers were related to methods to measure IFN-I pathway activation in RMDs in association with clinical outcomes. According to the different eligibility criteria depending on the research questions, overlapping sets of papers were included for each question (figure 1). Information about assay characteristics can be found in online supplemental text 8.

### Research question 1: what is the evidence that interferon measurement is useful in the diagnosis of RMDs?

**Figure 2:**
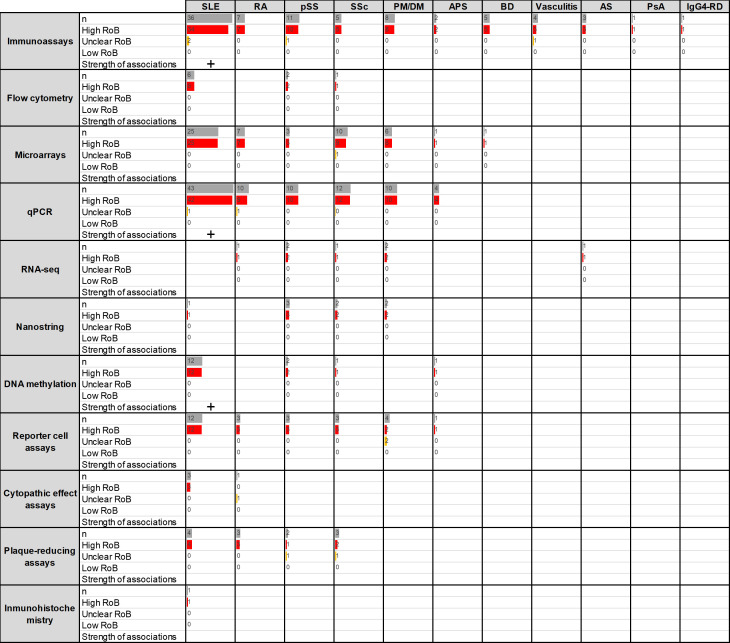
Summary of the studies reporting associations between IFN assays and diagnosis of RMDs (research question 1). Assays and RMDs are listed in rows and columns, respectively. The first number within each cell (n) represents the number of assays retrieved in the SLR for the corresponding technique and disease. The following numbers summarise the classification of these studies into RoB categories (high (red)/unclear (yellow)/low (green)). Bars are relative to the highest number of hits in the table. The strength of the associations (defined as the proportion of studies including diagnostic statistics) observed for each technique/disease combination is summarised as follows: ‘-’: no associations, ‘+’: <25%, ‘++’: 25%–50%, ‘+++’: 50%–75%, ‘++++’: >75%. APS, antiphospholipid syndrome; AS, ankylosing spondylitis; BD, Behçet’s disease; PM/DM, polymyositis/dermatomyositis; IFN, interferon; IgG4RD, IgG4-related disease; PsA, psoriatic arthritis; pSS, primary Sjögren’s syndrome; qPCR, quantitative PCR; RA, rheumatoid arthritis; RoB, risk of bias; RMD, rheumatic and musculoskeletal disease; RNA-seq, RNA sequencing; SLE, systemic lupus erythematosus; SLR, systematic literature review; SSc, systemic sclerosis.

A total of 188 papers were reviewed. Since many papers included more than one assay or disease group, these resulted in 305 analyses related to the diagnostic role of IFN-I pathway activation, distributed as follows: SLE (n=139), rheumatoid arthritis (RA, n=34), primary Sjögren’s syndrome (pSS, n=39), systemic sclerosis (SSc, n=39), dermatomyositis/polymyositis (DM/PM, n=32), antiphospholipid syndrome (APS, n=9), Behçet’s disease (BD, n=6), vasculitis (n=1), ankylosing spondylitis (n=1), IgG4-related disease (IgG4RD, n=1) and psoriatic arthritis (PsA, n=1) (figure 2). The distribution of the different assays was not uniform, SLE, pSS and SSc being the conditions reporting a higher number of different techniques. The vast majority of the studies were classified as having a high risk of bias.

#### Systemic lupus erythematosus

From a total of 139 assays reported,^7–123^ most of them used quantitative PCR (qPCR), immunoassays or microarrays. Most of the studies reported markedly increased IFN-I pathway activation in SLE compared with controls, but diagnostic statistics were scarcely reported and study designs did not usually recruit a prediagnosis population (online supplemental table 1). Studies on SLE using different methods consistently reported a substantial activation of the IFN-I pathway in patients, with percentages of patients classified as ‘high’ or ‘positive’ varying from 57% to 100%.

#### Rheumatoid arthritis

A total of 34 analyses were retrieved,^53 58 62 71 78 85 108 120 121 124–141^ qPCR, immunoassays and microarrays being the most widely used. Studies using methylation or flow cytometry were not found. Again, studies did not recruit prediagnosis populations and little information was given regarding diagnostic statistics (online supplemental table 2). Overall, although the activation of the IFN-I pathway was confirmed by different techniques, the extent of the activation was relatively lower than in SLE (patients with RA classified as ‘high’ or ‘positive’ ranged from 13% to 45%). Within-group analyses revealed differences across disease stages (early vs established disease) as well as among subsets of interferon-stimulated genes (ISGs) in RA populations.^85 124 134 136 137^

#### Primary Sjögren’s syndrome

In total, 39 analyses were found to evaluate differences between pSS and control populations.^68 84 89 97 103 110 116 120 121 127 142–157^ Most of the analyses were performed by immunoassays or qPCR, and diagnostic statistics were only reported in methylation assays (online supplemental table 3). Overall, the papers identified confirmed an IFN-I pathway activation in pSS, but to a lower degree than in SLE patient populations (51% to 70% patients with pSS classified as ‘high’ or ‘positive’).

#### Systemic sclerosis

Thirty-nine analyses were observed to evaluate differences between SSc and control populations^42 48 53 58 84 89 97 116 120 121 158–172^ (online supplemental table 4). The majority of them used qPCR or microarrays. Regarding immunoassay usage, most of them were directed against IFN-induced proteins, whereas the IFN-α protein was only assessed in one study. Different assays confirmed activation of the IFN-I pathway in SSc, but with important differences depending on clinical phenotypes (from 33% to 100%).^169^

#### Polymyositis/dermatomyositis

A total of 32 analyses were identified investigating the differences between patients with PM/DM and controls,^37 49 53 58 74 84 97 135 173–183^ mostly using qPCR and immunoassays (although assays studying IFN-induced proteins were lacking) (online supplemental table 5). Despite the lower number of papers and assays, results in PM/DM were highly consistent and found a strong upregulation of the IFN-I pathway in PM/DM (especially in DM), to a similar degree to SLE.

#### Other RMDs

The literature search identified a lower number of papers focused on APS (9),^31 61 88 105 184–186^ BD (6),^187–190^ vasculitis (4),^190–192^ ankylosing spondylitis (2),^189 193^ PsA (1)^194^ and IgG4-RD (1)^195^ compared with control-matched populations. Immunoassays were the most used assays.

#### Summary

Although there were a considerable number of papers reporting differences in IFN-I pathway activation between different RMDs and control populations, most of the studies were correlative/associative studies; diagnostic statistics (area under the curve (AUC), specificity, sensitivity and predictive values) were only reported in a limited number of studies and were of high risk of bias from a diagnostic standpoint (mostly due to inappropriate study designs, knowledge of diagnosis status, interval between tests and lack of appropriate disease controls). None of the studies had an appropriate design to evaluate the diagnostic performance (pretest/post-test probability, likelihood ratios, etc). Therefore, the strength of association with clinical outcome was classified as low/very low. Prospective studies with preclinical autoimmunity or at-risk populations point to an association between IFN-I pathway activation and fulfilment of classification criteria in individuals reaching a diagnostic clinical outcome, thus strengthening the promising relevance of IFN-I pathway activation in relation to disease diagnosis and reinforcing the need for better study designs to address this question.

Stronger evidence was obtained for SLE (in terms of number of analyses reported and different techniques), where results were largely homogeneous. Of note, the IFN-I pathway association in this group was found to be consistently elevated and relatively higher than the rest of the RMDs studied. Results were also consistent in PM/DM. Less consistent results were observed for pSS, SSc and RA, in which differences by clinical features were reported. An additional point illustrated by this literature collectively is that since IFN-I pathway activation is seen in many RMDs, these assays may differentiate inflammatory RMDs from non-inflammatory conditions but may not be able to differentiate between multiple possible RMDs.

### Research question 2: what is the evidence that interferon measurement reflects disease activity in RMDs?

**Figure 3:**
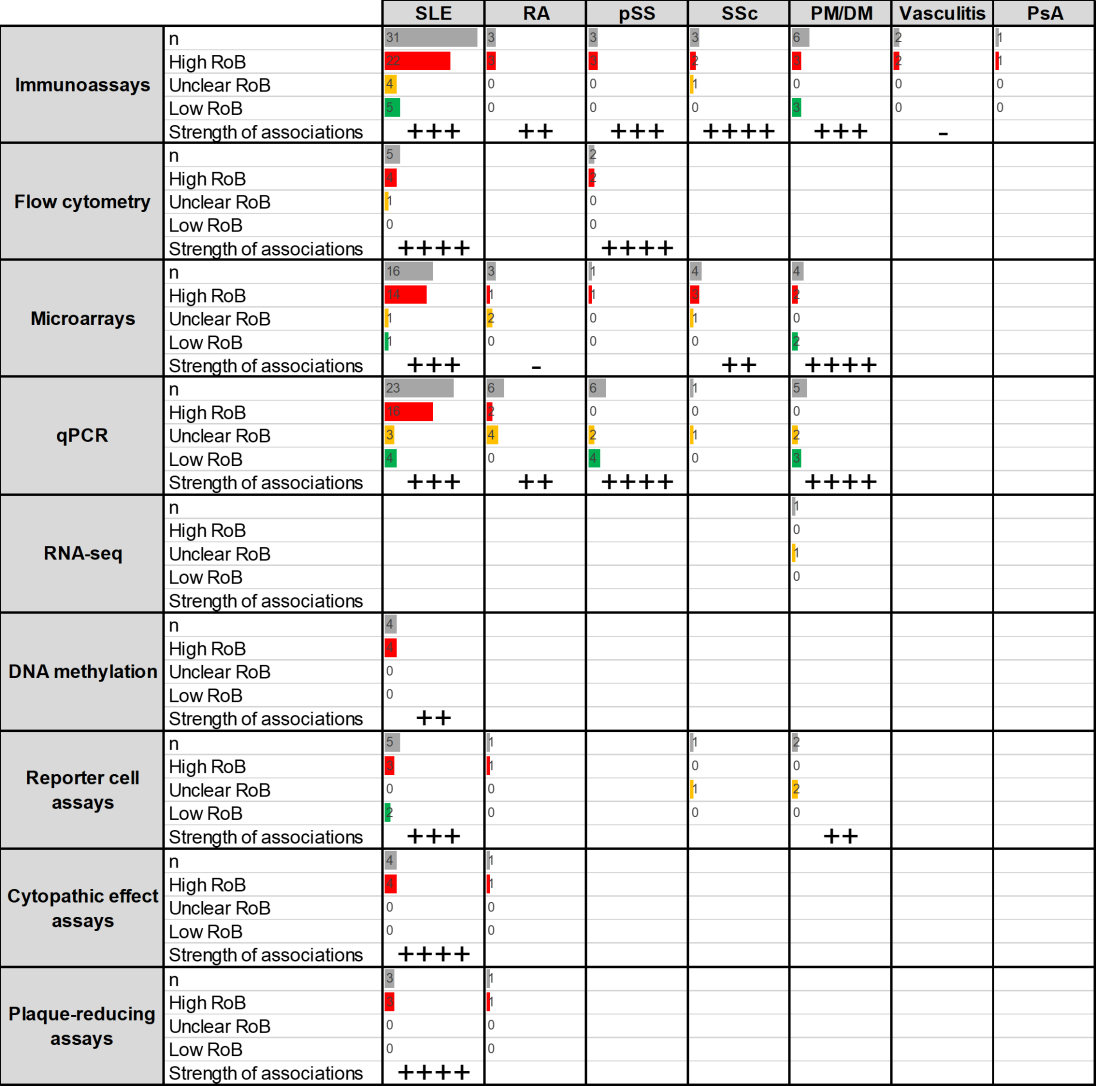
Summary of the studies reporting associations between IFN assays and disease activity (research question 2). Assays and RMDs are listed in rows and columns, respectively. The first number within each cell (n) represents the number of assays retrieved in the SLR for the corresponding technique and disease. The following numbers summarise the classification of these studies into RoB categories (high (red)/unclear (yellow)/low (green)). Bars are relative to the highest number of hits in the table. The strength of the associations (defined as the proportion of studies reporting positive associations between disease activity measures and IFN assays) observed for each technique/disease combination is summarised as follows: ‘-’: no associations, ‘+’: <25%, ‘++’: 25%–50%, ‘+++’: 50%–75%, ‘++++’: >75%. PM/DM, polymyositis/dermatomyositis; IFN, interferon; PsA, psoriatic arthritis; pSS, primary Sjögren’s syndrome; qPCR, quantitative PCR; RA, rheumatoid arthritis; RoB, risk of bias; RMD, rheumatic and musculoskeletal disease; RNA-seq, RNA sequencing; SLE, systemic lupus erythematosus; SLR, systematic literature review; SSc, systemic sclerosis.

A total of 122 papers were retrieved, which lead to 153 analyses related to the association between IFN-I pathway activation and disease activity distributed as follows: SLE (97), RA (15), pSS (12), SSc (9), DM/PM (17), vasculitis (2) and PsA (1) (figure 3). A significant proportion of studies were of high or unclear risk of bias (mostly due to the lack of confounding identification, adjustment and analysis). Again, the distribution of the different assays was not uniform, SLE being the condition reporting the highest number of different techniques and usage of several disease activity instruments.

#### Systemic lupus erythematosus

A total of 97 analyses of the association between disease activity and IFN-I pathway activation were identified,^7–9 12 13 15 17 19 20 22 24 25 27–29 31 32 36–42 45 47–51 53 59 64 66 67 69 72 75–78 81 82 85 86 90 92 93 98 99 101 106 107 115 116 118–120 122 123 130 196–213^ mostly being immunoassays, qPCR and microarrays (online supplemental table 6). Overall, IFN-I pathway activation has been reported to reflect disease activity in patients with SLE, with results being highly consistent across populations and disease activity instruments, although the inclusion of serological biomarkers in the SLE Disease Activity Index (SLEDAI) is an additional bias for that instrument in the validation of other biomarkers. These studies were mainly cross-sectional. Regardless of the technique used, studies with a low risk of bias were consistent in reporting a positive association. A higher consistency was observed for flow cytometry, microarrays and functional assays (cytopathic and plaque-reducing assays). When reported, correlation coefficients were homogeneous and moderate (0.3–0.6). Of note, the associations with disease activity may exhibit some differences depending on the organ affected,^47 75^ thus suggesting that clinical domains should be taken into consideration when interpreting these associations and when comparing results from different populations or backgrounds.

#### Rheumatoid arthritis

From a total of 16 analyses retrieved,^53 71 85 124 125 129 131 136 137 140 141 214 215^ most of the evidence came from qPCR and microarrays (online supplemental table 7). Most of the studies were of high or unclear risk of bias. Overall, there is some evidence that IFN-I pathway activation correlates to disease activity, but a low consistency across assays and important differences across disease stages were reported. When mentioned, coefficient correlations were often low and highly heterogeneous (ranging from −0.3 to 0.5).

#### Primary Sjögren’s syndrome

A total of 12 analyses were found,^89 147 148 151–153 216 217^ mostly using qPCR methods and immunoassays (online supplemental table 8). There is limited evidence that IFN-I pathway activation correlates with disease activity in patients with pSS, results being more consistent with flow cytometry and qPCR assays (especially those of low risk of bias, although most were classified as having high risk). As for the SLEDAI in SLE, the EULAR pSS Disease Activity Index instrument includes some immune biomarkers, which may introduce additional bias when validating other biomarkers.

#### Systemic sclerosis

The association between disease activity in SSc and IFN-I pathway activation was analysed in nine studies,^48 53 158 161 164 165 167^ mostly from microarrays and immunoassays (online supplemental table 9) and of high/unclear risk of bias. There was limited evidence of a positive association, mostly with immunoassays, qPCR and functional assays, despite the low number of the latter.

#### Polymyositis/dermatomyositis

A total of 17 analyses were retrieved from the literature,^49 53 173 176–179 181 183 218 219^ mostly reporting on immunoassays, qPCR and microarrays and showing a positive association with disease activity using different instruments (online supplemental table 10). Their results were very consistent across methods, especially in qPCR, microarrays and RNA sequencing, as well as in immunoassays to a lower extent. Correlation coefficients were homogeneous and moderate (from 0.4 to 0.8).

#### Other RMDs

Evidence was weaker in vasculitis (2)^190^ and PsA (1),^194^ all using immunoassays.

#### Summary

In general, there was evidence associating IFN-I pathway activation with disease activity in RMDs, most consistently in SLE and PM/DM, but also in RA and pSS to a lower extent. The number of different methods largely differed across RMDs. Most of the studies focused on qPCR, microarrays and immunoassays, with variable results across diseases. Despite being less reported, flow cytometry and functional assays were very likely to consistently exhibit positive associations with disease activity across RMDs. In addition to assay characteristics, variables such as the components of the disease activity instruments and clinical features (organ involvement, disease stage, etc) should be considered.

Overall, most of the studies were cross-sectional and associative and of unclear/high risk of bias. Despite the positive associations reported, the added clinical utility of measuring IFN-I pathway activation to monitor disease activity or whether they outperformed the current clinical instruments was not generally evaluated.

### Research question 3: what is the evidence that interferon measurement is useful for the prognosis (natural history) of clinical status in RMDs?

**Figure 4:**
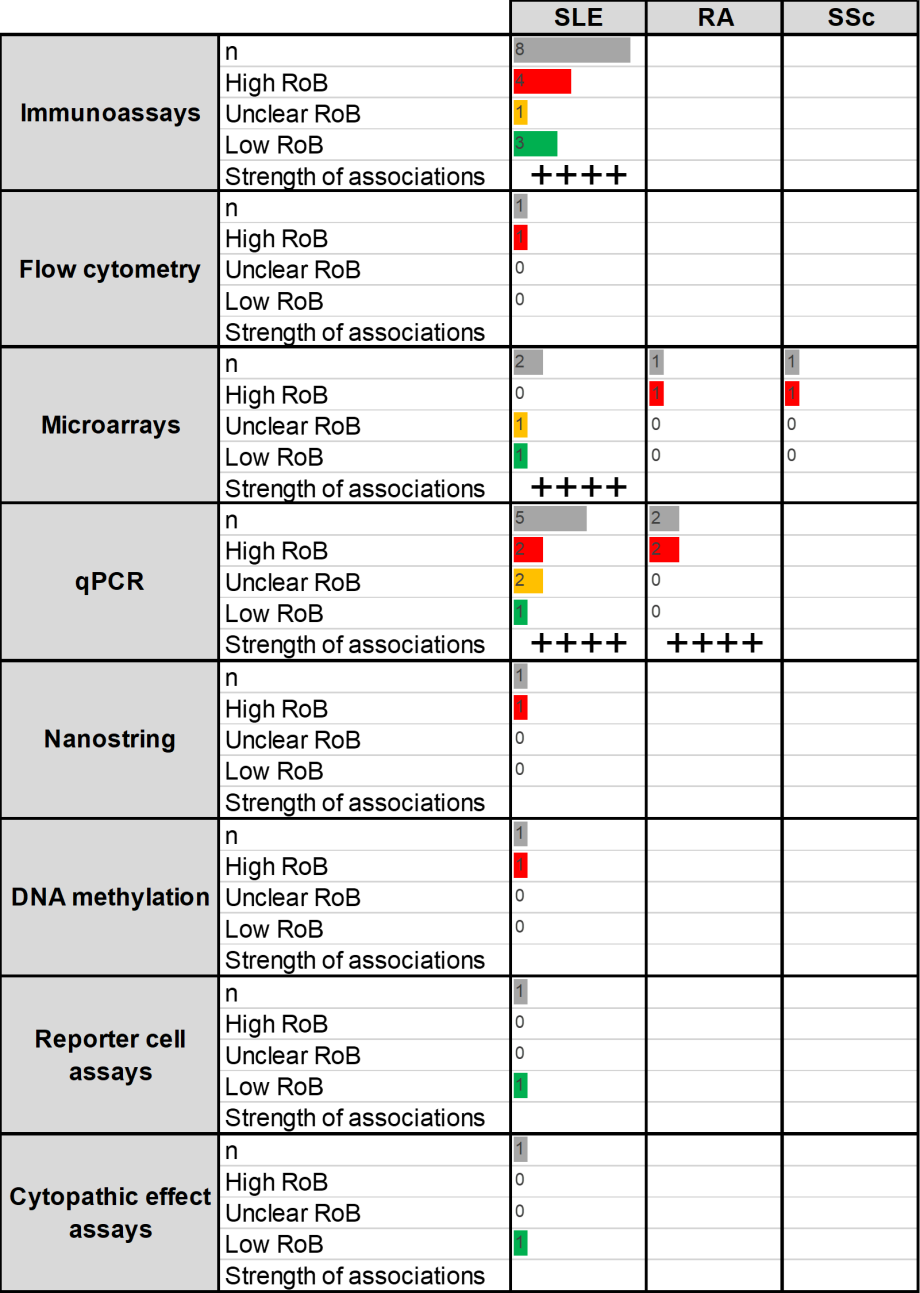
Summary of the studies reporting associations between IFN assays and disease prognosis (research question 3). Assays and RMDs are listed in rows and columns, respectively. The first number within each cell (n) represents the number of assays retrieved in the SLR for the corresponding technique and disease. The following numbers summarise the classification of these studies into RoB categories (high (red)/unclear (yellow)/low(green)). Bars are relative to the highest number of hits in the table. The strength of the associations (defined as the proportion of studies reporting IFN assays prospectively predicted disease outcomes) observed for each technique/disease combination is summarised as follows: ‘-’: no associations, ‘+’: <25%, ‘++’: 25%–50%, ‘+++’: 50%–75%, ‘++++’: >75%. IFN, interferon; qPCR, quantitative PCR; RA, rheumatoid arthritis; RoB, risk of bias; RMD, rheumatic and musculoskeletal disease; SLE, systemic lupus erythematosus; SLR, systematic literature review; SSc, systemic sclerosis.

A total of 20 papers were retrieved, resulting in 24 analyses related to the association between IFN-I pathway activation and disease prognosis as follows: SLE (20), RA (3) and SSc (1) (figure 4).

#### Systemic lupus erythematosus

A total of 20 analyses were retrieved relevant to the use of IFN-I pathway assays to predict flare development in patients with SLE,^36 45 54 75 81 83 92 97 104 107 198 199 202 204 220 221^ mostly being immunoassays and qPCR (online supplemental table 11). Most of the analyses showed that IFN-I pathway activation could predict flare occurrence, defined by different clinical instruments and using different biosamples, along different follow-up periods (from 6 to 24 months), with a high consistency across methods. Studies with a low risk of bias (including appropriate confounding handling) were consistent in showing a positive predictive effect. There is some evidence that IFN-I pathway activation is a better predictor of flare (based on higher AUC, sensitivity or specificity values) than classical features (clinical or laboratory findings). However, other studies failed to confirm this. Of note, comparative analyses evaluating the added value of assays measuring IFN-I pathway activation over existing clinical instruments were scarcely reported. Therefore, the incremental value provided by analysis of the IFN-I pathway activation is uncertain.

Additionally, the use of IFN-I pathway assays in predicting progression from pre-clinical autoimmunity to clinical disease (SLE and/or others) has been also evaluated in a study with low risk of bias with positive results.^83^ No associations were found in another study with a high risk of bias.^97^ However, the type of assays, preclinical autoimmunity population studied and clinical outcomes limit comparative analyses.

#### Rheumatoid arthritis

The use of IFN-I pathway assays to predict prognosis in RA was evaluated in three analyses,^137 222 223^ focused on progression from arthralgia to clinical RA (2) and prediction of disease activity at follow-up (1) (online supplemental table 12). Microarray (1) and qPCR (1) studies supported an association between IFN-I pathway activation and progression to clinical RA. All studies were of high risk of bias.

#### Systemic sclerosis

One study was found to evaluate the association between IFN-I pathway activation and disease progression in SSc,^163^ thus reporting a negative correlation with increased forced vital capacity whereas no association was found with modified Rodnan skin score at 26 months (high risk of bias) (online supplemental table 13).

#### Summary

Evidence was supportive of the use of assays measuring IFN-I pathway activation in predicting disease prognosis, although depending on the prognostic outcome and disease context. Stronger evidence related to the prediction of flares in SLE populations, mostly by immunoassays and qPCR methods. A single higher-quality study supported the prediction of progression to clinical disease in antinuclear antibody–positive individuals. Better clinical characterisation of these populations, confounder identification and handling as well as clinical validation remain suboptimal for other questions and contexts.

### Research question 4: what is the evidence that interferon measurement is useful for the prognosis of the response to treatment in RMDs?

**Figure 5:**
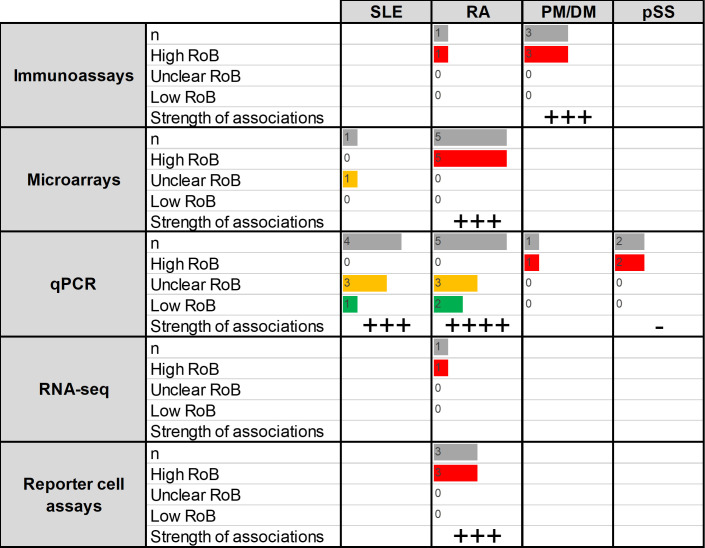
Summary of the studies reporting associations between IFN assays and response to treatment in RMDs (research question 4). Assays and RMDs are listed in rows and columns, respectively. The first number within each cell (n) represents the number of assays retrieved in the SLR for the corresponding technique and disease. The following numbers summarise the classification of these studies into RoB categories (high (red)/unclear (yellow)/low (green)). Bars are relative to the highest number of hits in the table. The strength of the associations (defined as the proportion of studies reporting IFN assays predicted response to treatment) observed for each technique/disease combination is summarised as follows: ‘-’: no associations, ‘+’: <25%, ‘++’: 25%–50%, ‘+++’: 50%–75%, ‘++++’: >75%. PM/DM, polymyositis/dermatomyositis; IFN, interferon; pSS, primary Sjögren’s syndrome; qPCR, quantitative PCR; RA, rheumatoid arthritis; RoB, risk of bias; RMD, rheumatic and musculoskeletal disease; RNA-seq, RNA sequencing; SLE, systemic lupus erythematosus; SLR, systematic literature review.

A total of 23 papers were retrieved, leading to 26 analyses related to the association between IFN-I pathway activation and response to treatment distributed as follows: SLE (5), RA (15), PM/DM (4) and pSS (2) (figure 5).

#### Systemic lupus erythematosus

A total of five analyses evaluated the association between IFN-I pathway activation, mostly using qPCR methods, and response to treatment with four different agents, including therapies targeting the IFN-I pathway^54 224–227^ (online supplemental table 14). One study with tabalumab (anti-B-cell-activating factor) using microarrays failed to show any association.^54^ Among studies with antibodies against IFN-α protein, one study (using sifalimumab) showed no association,^227^ whereas another study (using rontalizumab) concluded that IFN-I pathway activation could predict clinical response. In this study, better response to IFN-I blockade was observed in patients with low IFN-I pathway activation.^225^ On the contrary, studies with anifrolumab (2)—all randomised controlled trial (RCT) with low/unclear risk of bias, using different clinical response criteria—reported that increased IFN-I pathway activation was predictive of better clinical response, in contrast to results for rontalizumab.^224 226^

#### Rheumatoid arthritis

The use of IFN-I pathway activation to predict treatment outcomes was evaluated in 15 analyses,^125 131 136–139 214 215 222 228–232^ mostly by microarrays and qPCR methods (online supplemental table 15). IFN-I pathway activation was found to predict clinical response to anti-tumour necrosis factor (anti-TNF)^125 131 136 138 139 214 232^ (unclear/high risk of bias) and the anti-CD20 monoclonal antibody rituximab^215 228 230 231^ (low/unclear risk of bias) using different assays. However, the direction of the association between IFN-I pathway activation and clinical response to anti-TNF treatment was different in studies using different assays, biosamples and sample timings. Functional assays highlighted the need of combined qualitative (ie, the relative contribution of the actual IFN proteins underlying IFN-I pathway activation) and quantitative approaches (ie, the absolute level of IFN-I pathway activation).^232^ There was also some consistent but limited evidence on conventional synthetic disease modifying antirheumatic drug (csDMARD),^136 137^ and rather limited with tocilizumab (anti-interleukin 6 monoclonal antibody).^229^

#### Polymyositis/dermatomyositis

Four analyses addressing the use of IFN pathway activation and clinical response in PM/DM were retrieved^233 234^ (online supplemental table 16). The association with clinical response to combined immunosuppressive agents (3)^233^ was not consistent among assays, with significant associations being observed in qPCR and immunoassays (only with some outcomes). The results with rituximab (1)^234^ were significant although variable across clinical outcomes and antibody status.

#### Primary Sjögren’s syndrome

The use of IFN-I pathway assays to predict treatment outcomes in pSS was evaluated in two studies^152 235^ (online supplemental table 17). No significant associations were found.

#### Summary

There was consistent evidence that measuring the IFN-I pathway activation by gene assays predicted better response to IFN-I targeting therapies in SLE across RCTs. Other than this, while there were a number of relatively high-quality studies reporting an association between IFN-I pathway activation assays and response to therapy in RMDs, especially in SLE, some of the results appeared contradictory. A potential issue here relates to the properties of the IFN-I pathway biomarkers described above. These biomarkers associate with baseline disease activity, clinical features and serological markers, which may themselves predict response to both standard of care and investigational targeted therapy. To what extent IFN-I pathway activation outperforms these variables and existing instruments is yet to be elucidated.

### Research question 5: what is the evidence that interferon measurement is responsive to changes with changing disease status or treatment?

**Figure 6:**
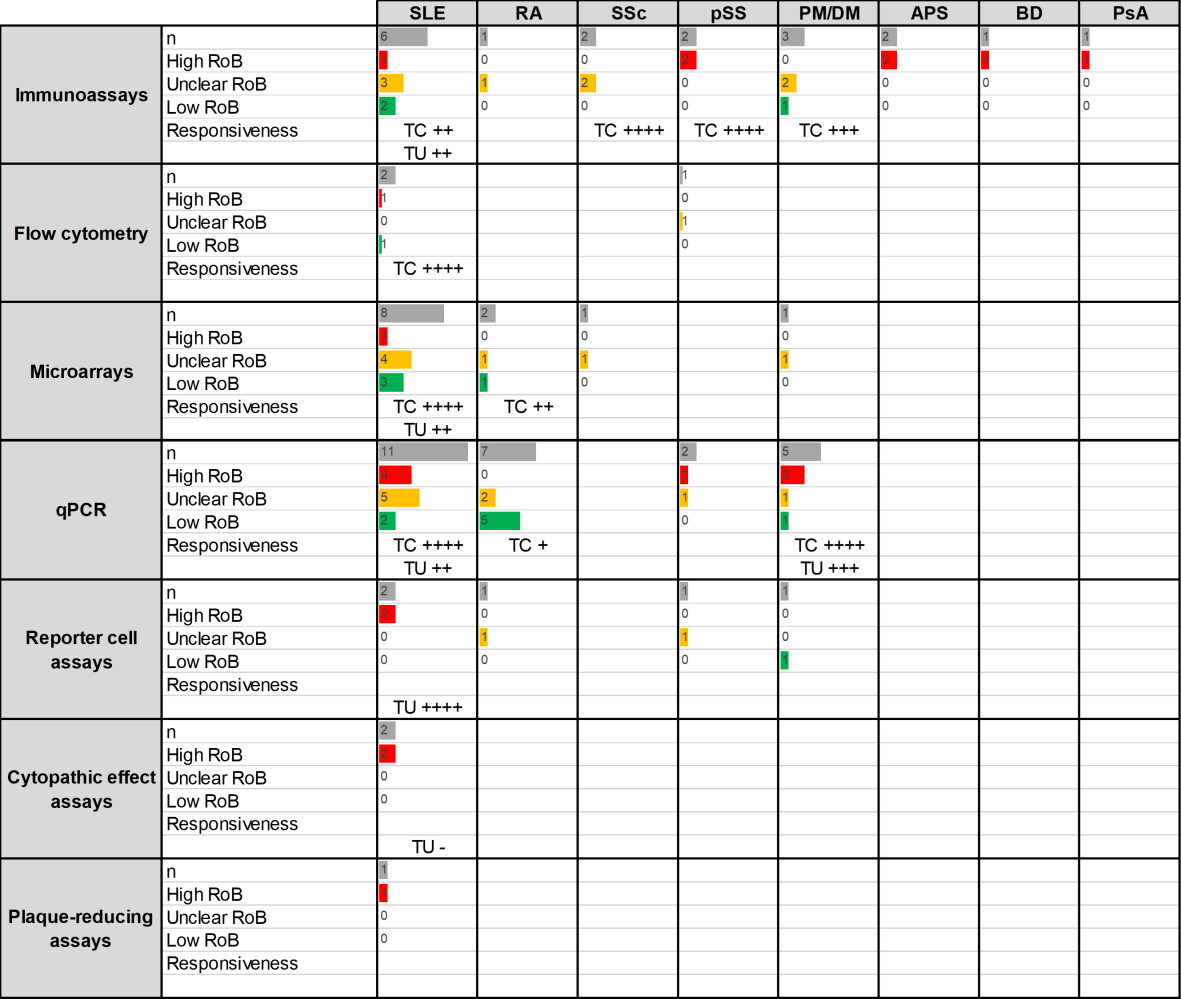
Summary of the studies reporting responsiveness to change of IFN assays in RMDs (research question 5). Assays and RMDs are listed in rows and columns, respectively. The first number within each cell (n) represents the number of assays retrieved in the SLR for the corresponding technique and disease. The following numbers summarise the classification of these studies into RoB categories (high (red)/unclear (yellow)/low (green)). Bars are relative to the highest number of hits in the table. The strength of the associations (defined as the proportion of (studies reporting significant changes) observed for each technique/disease combination in patients with treatment changes (TC) or treatment unchanged (TU, usual/standard care) observed for each technique/disease combination is summarised as follows: ‘-’: no associations, ‘+’: <25%, ‘++’: 25%–50%, ‘+++’: 50%–75%, ‘++++’: >75%.APS, antiphospholipid syndrome; BD, Behçet’s disease; PM/DM, polymyositis/dermatomyositis; IFN, interferon; PsA, psoriatic arthritis; pSS, primary Sjögren’s syndrome; qPCR, quantitative PCR; RA, rheumatoid arthritis; RoB, risk of bias; RMD, rheumatic and musculoskeletal disease; SLE, systemic lupus erythematosus; SLR, systematic literature review; SSc, systemic sclerosis.

A total of 59 papers were retrieved, leading to 63 analyses related to the association between IFN-I pathway activation and assay responsiveness (change over time) as follows: SLE (31), RA (11), SSc (3), pSS (6) and PM/DM (10) (figure 6).

#### Systemic lupus erythematosus

Among 32 analyses retrieved, 18 studies analysed the changes in IFN-I pathway activation on initiation of novel treatment or modification of treatment dosages,^15 40 54 59 64 78 79 90 94 130 208 236–239^ whereas 14 analysed fluctuations in the absence of group-level changes in treatments^9 47 72 82 108 110 117 123 196 204 206 213 240–242^ (online supplemental table 18). The use of novel treatment regimens was associated with decreases in IFN-I pathway activation, mostly with drugs targeting this pathway,^59 64 90 94 130 238 239^ but also with high doses of glucocorticoids (oral or intravenous).^40 208^ Studies with a low risk of bias were consistent in this regard. These changes were observed in the short (few days) and the long term (until 6 months) and were consistent across methods. Biological drugs not targeting the IFN-I pathway (omalizumab: anti-IgE,^237^ tabalumab: anti-B-cell activating factor^54^) and other agents (hydroxychloroquine,^15^ vitamin D^236^) did not modulate IFN-I pathway activation. In most of the studies with no group-level changes in treatment, no fluctuations (5) or uncertain patterns (2) were observed in IFN-I measurements. Of note, microarrays revealed heterogeneity among expression modules. For example, module 5.12 was more responsive to change in clinical status than module 1.2, with the latter including the ISGs most commonly measured in other qPCR studies.^43 47^ Studies finding fluctuations in IFN-I pathway activation (6) reported parallel changes in disease trajectories (disease exacerbation, flares, remission).

#### Rheumatoid arthritis

From 18 analyses identified in the literature^85 124 136 137 214 222 228 232 243 244^ almost all came from studies analysing changes in treatments (online supplemental table 19). Studies in patients initiating anti-TNF treatment (5) did not reveal changes in IFN-I pathway activation,^85 136 214 222^ except in a study using a functional assay.^232^ The retrieved studies used different methods and a similar timeframe (from 1 to 3 months). Evidence from studies with other agents (anakinra (1),^243^ combined csDMARDs (1)^137^ and rituximab (1)^228^) was more limited but suggested potential changes (in different directions) in IFN-I pathway activation on initiation of treatment.

#### Systemic sclerosis

Among three analyses retrieved from the literature, two came from studies with changes in treatment regimens to immunosuppression with cyclophosphamide and revealed suppression of the IFN-I pathway activation using different methods (immunoassays and microarrays)^163^ (online supplemental table 20). Studies with no group-level changes in treatment^161^ found unaltered IFN-I pathway activation in long follow-ups (>2 years).

#### Primary Sjögren’s syndrome

From six analyses identified in the literature,^142 148 152 153 157^ five of them revealed suppression of the IFN-I pathway activation in patients starting new treatments, mainly rituximab (2)^142^ and HCQ (2)^148 152^ (online supplemental table 21). Only one analysis was retrieved with no changes in treatment and revealed unaltered IFN-I pathway activation.

#### Polymyositis/dermatomyositis

Among 10 analyses retrieved from the literature, 7 analysed the effect of novel treatments and most of them revealed changes in IFN-I pathway activation in relation to clinical improvement along different time points^178 233 234 245–247^ (online supplemental table 22). Results were more consistent with immunosuppressive-combined regimens (3)^178 233^ and not consistent with rituximab (2),^234 245^ whereas other drugs (sifalimumab and the anti-TNF infliximab) were less studied. Fluctuations in IFN-I pathway activation were also found in studies with no changes in treatments (3),^176 177 219^ but changes in disease activity were reported in parallel in all cases. Patients with no or little changes in disease activity were found not to exhibit fluctuations in IFN-I pathway activation.^176 177^

#### Other RMDs

Analyses of changes in IFN pathway activation were also identified in vasculitis (1)^190^ and PsA (1)^194^ populations, with very low sample sizes in both cases (n<10).

#### Summary

IFN-I pathway activation seemed to be stable over time at the group level across different RMDs and different assays in the absence of systematic treatment changes or disease activity fluctuations (exacerbation or remission). However, in studies in which groups of patients started the same treatment, there was evidence that certain treatments can modulate IFN-I pathway activation, especially drugs targeting the IFN-I pathway and high-dose glucocorticoids; the effect of other agents seems to be weaker and differed across RMDs. Of note, not all ISGs or gene modules exhibited the same assay responsiveness.

## Discussion

Despite the pivotal role of IFN-I in the pathogenesis of RMDs, the numerous assays for this pathway have so far not successfully translated into clinical practice. The aim of this SLR was to provide a comprehensive review of the existing evidence to understand causes, identify gaps and provide solid foundations to enable future clinical and research applications of IFN-I assays. This is the first study where the evidence underlying the analysis of IFN-I pathway activation in rheumatology is investigated in a systematic manner. A key strength of this review is that it provides an overall picture of findings, since a large number of RMDs were included and the clinical questions formulated covered the entire disease process.

Evidence has been encouraging for the potential role of IFN-I pathway activation as a biomarker in several RMDs with different clinical applications and outcomes. However, despite extensive research over the last decades, our SLR revealed (1) an enormous diversity of assays, (2) a high methodological heterogeneity, also related to reporting and analysis and (3) a number of important flaws. Several issues were detected in study design, clinical validation, outcome definition and assessment, gold standard definition, as well as in the analysis and reporting of the results. These issues prevented the possibility of performing pooled analyses to generate robust, first-level clinical evidence. Taken together, these points may account for the lack of transition of IFN-I assays into routine care and emphasise the need for harmonisation of the clinical and experimental requirements along the whole process (from sample choice and collection to results reporting). Although our SLR was focused on RMDs, the observed methodological concerns are not rheumatology-specific, and due to the involvement of IFN-I pathway activation in other areas,^248–250^ our findings might be generalisable to other clinical fields.

An important message from our SLR is that although there was a certain degree of consistency among IFN-I pathway assays for a given research/clinical question, there was not a single, universal assay that can satisfy all the needs. This may be explained, at least in part, by the fact that by measuring different components of the IFN-I pathway, they likely provide different information. From a biological standpoint, there is a huge difference in measuring the production of IFN proteins (which belong to different subtypes in different proportions depending on the stimulus and require highly sensitive and reliable assays) compared with the cellular response(s) to IFNs (which can be analysed at functional or genetic levels by several read-outs and may differ in their specificity to the IFN proteins). These differences may provide a different degree of added value for a clinical question. In this sense, there is a need for more comparative studies using different assays, both in terms of head-to-head analyses to allow direct and indirect comparisons across assays, but also in terms of simultaneous assessments in several RMDs to evaluate if the clinical added value is similar across the RMD spectrum or if, on the contrary, it needs to be regarded as disease-specific. Furthermore, whether this added clinical value may be seen by using combinations of different assays or combinations with other signatures and biomarkers remains to be explored.^43 251^

Another remarkable finding from this review was that the level of evidence about the value of IFN-I assays across RMDs and clinical outcomes within a single RMD was largely skewed. This may reflect that the use of IFN-I assays has been proposed to resolve disease-specific unmet clinical needs, so it may be difficult to compare its overall value in different disease scenarios. Moreover, differences in added value may be also obscured by the use of different treatment modalities across different RMDs, hence underscoring the need of evaluating untreated populations and/or appropriate analysis adjustments by treatment.^252^

From a clinical perspective, an important flaw detected in the existing literature was the noticeable lack of the well-designed diagnostic studies, despite yielding the highest number of hits. Regarding the remaining clinical endpoints, evidence was consistent about the use of IFN-I assays to predict disease prognosis in patients with SLE (flare occurrence), as well as to monitor disease activity (although with less evidence about its added value and potential confounding due to serological markers in composite indices). In RA, heterogeneous associations were linked, at least in part, to the disease stage, so the choice and target(s) of the assay may need to be adapted along the disease course. An equivalent picture was observed in SLE populations depending on organ involvement. It must be noted that IFN-I assays have been also linked to several clinical features and patient-reported outcomes. Recent evidence suggests that some assays fail to exhibit an association with fatigue in SLE and pSS,^96 253 254^ whereas some immunoassays^255 256^ and gene expression assays^257–259^ showed conflicting results, hence strengthening the need for careful selection of assays and target(s) depending on the clinical question.

Regarding the prediction of response to treatment, evidence was stronger for SLE in general and consistent for drugs targeting the IFN-I pathway. Following the analysis of this SLR, an additional major study with a pooled population from two phase III RCTs with anifrolumab demonstrated a better response in patients with a high IFN-I pathway activation across different clinical endpoints.^260^ One further RCT of a non-IFN-targeted therapy (iberdomide) also showed positive results for the prediction of response using a similar gene assay.^261^ Therefore, the latest evidence reassured the findings of our SLR. Furthermore, IFN-I pathway activation might be related to the progression from preclinical autoimmunity to clinical disease, with limited evidence coming from SLE-related and RA-related studies. However, it is important to note that the incremental, added value provided by IFN-I assays was difficult to evaluate due to the lack of established instruments which to be validated. Finally, the analyses of the responsiveness of IFN-I assays yielded a relatively uniform message across RMDs. IFN-I pathway activation measurements seemed to be relatively stable over time in the absence of systematic changes in treatments or disease status.

This review has some limitations. Although we used a sensitive approach to identify all the available studies, a potential effect of publication bias cannot be excluded, which may lead to an overestimation of ‘positive’ results (more likely to be published). Moreover, the heterogeneity observed prevented the use of meta-analyses or pooled analyses. Moreover, RMDs were grouped according to classification criteria to allow a global comparison across conditions. However, whether differences by disease stage, clinical features (including patient-reported outcomes), and treatment modalities should be considered to evaluate the clinical value of IFN-I pathway activation in disease subsets has not been addressed in the present SLR. Regarding bias assessments, it is important to note that for some research questions, the results of the small number of higher-quality studies are not negated by the less certain or contradictory findings of more numerous low-quality studies.

In conclusion, evidence is supportive of the clinical value of IFN-I pathway activation in RMDs, although the results herein reported revealed a high methodological heterogeneity, risk of bias and important flaws in IFN-I research in this field. In addition to putting figures into these aspects, this SLR urges a need for harmonisation and implementation of a minimum number of elements around these flaws when reporting and performing future research. This SLR informs the ongoing EULAR points to consider for the measurement, reporting and application of IFN-I pathway activation assays in clinical and research practice.

## Data Availability

No data are available. All data are included within the article or uploaded as supplementary information, and no datasets were generated and/or analysed for this study.

## References

[1] Ivashkiv LB, Donlin LT. Regulation of type I interferon responses. Nat Rev Immunol 2014;14:36–49. 10.1038/nri3581 24362405 PMC4084561

[2] Psarras A, Emery P, Vital EM. Type I interferon-mediated autoimmune diseases: pathogenesis, diagnosis and targeted therapy. Rheumatology (Oxford) 2017;56:1662–75. 10.1093/rheumatology/kew431 28122959

[3] Crow MK. Type I interferon in organ-targeted autoimmune and inflammatory diseases. Arthritis Res Ther 2010;12 Suppl 1:S5. 10.1186/ar2886 21303493 PMC2991778

[4] Rönnblom L, Eloranta M-L. The interferon signature in autoimmune diseases. Curr Opin Rheumatol 2013;25:248–53. 10.1097/BOR.0b013e32835c7e32 23249830

[5] van der Heijde D, Aletaha D, Carmona L, et al. 2014 update of the EULAR standardised operating procedures for EULAR-endorsed recommendations. Ann Rheum Dis 2015;74:8–13. 10.1136/annrheumdis-2014-206350 25261577 PMC4283681

[6] Burska AN, Rodríguez-Carrio J, Biesen R, et al. Type I interferon pathway assays in studies of rheumatic and musculoskeletal diseases: a systematic literature review informing EULAR points to consider. RMD Open 2023. 10.1136/rmdopen-2022-002876 PMC9990675 36863752

[7] Abdel Galil SM, El-Shafey AM, Abdul-Maksoud RS, et al. Interferon alpha gene expression and serum level association with low vitamin D levels in Egyptian female patients with systemic lupus erythematosus. Lupus 2018;27:199–209. 10.1177/0961203317716321 28659049

[8] Becker-Merok A, Østli-Eilersten G, Lester S, et al. Circulating interferon-α2 levels are increased in the majority of patients with systemic lupus erythematosus and are associated with disease activity and multiple cytokine activation. Lupus 2013;22:155–63. 10.1177/0961203312468964 23213068

[9] Fragoso-Loyo H, Atisha-Fregoso Y, Núñez-Alvarez CA, et al. Utility of interferon-α as a biomarker in central neuropsychiatric involvement in systemic lupus erythematosus. J Rheumatol 2012;39:504–9. 10.3899/jrheum.110983 22247358

[10] Gao L, OConnell M, Allen M, et al. Bone marrow mesenchymal stem cells from patients with SLE maintain an interferon signature during in vitro culture. Cytokine 2020;132:154725. 10.1016/j.cyto.2019.05.012 31153744

[11] Mozo L, López P, Caminal-Montero L, et al. Anti-ribosomal P antibodies are associated with elevated circulating ifnα and IL-10 levels in systemic lupus erythematosus patients. Lupus 2014;23:1477–85. 10.1177/0961203314546020 25107939

[12] Oke V, Brauner S, Larsson A, et al. IFN-λ1 with th17 axis cytokines and IFN-α define different subsets in systemic lupus erythematosus (SLE). Arthritis Res Ther 2017;19:139. 10.1186/s13075-017-1344-7 28619037 PMC5471711

[13] Postal M, Sinicato NA, Peliçari KO, et al. Clinical and serological manifestations associated with interferon-α levels in childhood-onset systemic lupus erythematosus. Clinics (Sao Paulo) 2012;67:157–62. 10.6061/clinics/2012(02)11 22358241 PMC3275113

[14] Shahin D, El-Refaey AM, El-Hawary AK, et al. Serum interferon-alpha level in first degree relatives of systemic lupus erythematosus patients: correlation with autoantibodies titers. Egyptian Journal of Medical Human Genetics 2011;12:139–46. 10.1016/j.ejmhg.2011.06.009

[15] Willis R, Seif AM, McGwin G, et al. Effect of hydroxychloroquine treatment on pro-inflammatory cytokines and disease activity in SLE patients: data from LUMINA (LXXV), a multiethnic US cohort. Lupus 2012;21:830–5. 10.1177/0961203312437270 22343096 PMC3808832

[16] Ye H, Wang X, Wang L, et al. Full high-throughput sequencing analysis of differences in expression profiles of long noncoding rnas and their mechanisms of action in systemic lupus erythematosus. Arthritis Res Ther 2019;21:70. 10.1186/s13075-019-1853-7 30836987 PMC6402184

[17] Yin Z, Huang J, He W, et al. Serum level of eight cytokines in Han Chinese patients with systemic lupus erythematosus using multiplex fluorescent microsphere method. Cent Eur J Immunol 2014;39:228–35. 10.5114/ceji.2014.43728 26155129 PMC4440016

[18] Zecevic L, Karamehic J, Coric J, et al. Potential immune biomarkers in diagnosis and clinical management for systemic lupus erythematosus. J Med Biochem 2018;37:163–71. 10.1515/jomb-2017-0048 30581353 PMC6294097

[19] Zhuang H, Narain S, Sobel E, et al. Association of anti-nucleoprotein autoantibodies with upregulation of type I interferon-inducible gene transcripts and dendritic cell maturation in systemic lupus erythematosus. Clin Immunol 2005;117:238–50. 10.1016/j.clim.2005.07.009 16126005

[20] Fernández Matilla M, Grau García E, Fernández-Llanio Comella N, et al. Increased interferon-1α, interleukin-10 and BLyS concentrations as clinical activity biomarkers in systemic lupus erythematosus. Medicina Clínica (English Edition) 2019;153:225–31. 10.1016/j.medcle.2019.07.003 30795903

[21] Lood C, Amisten S, Gullstrand B, et al. Platelet transcriptional profile and protein expression in patients with systemic lupus erythematosus: up-regulation of the type I interferon system is strongly associated with vascular disease. Blood 2010;116:1951–7. 10.1182/blood-2010-03-274605 20538795

[22] Hashad DI, Abdelmagid MH, Elsherif SH. MicroRNA146a expression in lupus patients with and without renal complications. J Clin Lab Anal 2012;26:35–40. 10.1002/jcla.20501 24833532 PMC6807509

[23] Kanakoudi-Tsakalidou F, Farmaki E, Tzimouli V, et al. Simultaneous changes in serum HMGB1 and IFN-α levels and in LAIR-1 expression on plasmatoid dendritic cells of patients with juvenile SLE. new therapeutic options? Lupus 2014;23:305–12. 10.1177/0961203313519157 24399813

[24] Ma C, Jiao Y, Zhang J, et al. Elevated plasma level of HMGB1 is associated with disease activity and combined alterations with IFN-α and TNF-α in systemic lupus erythematosus. Rheumatol Int 2012;32:395–402. 10.1007/s00296-010-1636-6 21120500

[25] Mandal M, Tripathy R, Panda AK, et al. Vitamin D levels in Indian systemic lupus erythematosus patients: association with disease activity index and interferon alpha. Arthritis Res Ther 2014;16:R49. 10.1186/ar4479 24507879 PMC3979045

[26] Pacheco Y, Barahona-Correa J, Monsalve DM, et al. Cytokine and autoantibody clusters interaction in systemic lupus erythematosus. J Transl Med 2017;15:239. 10.1186/s12967-017-1345-y 29178890 PMC5702157

[27] Robak E, Smolewski P, Wozniacka A, et al. Relationship between peripheral blood dendritic cells and cytokines involved in the pathogenesis of systemic lupus erythematosus. Eur Cytokine Netw 2004;15:222–30. 15542447

[28] Nielsen CT, Lood C, Ostergaard O, et al. Plasma levels of galectin-3-binding protein reflect type I interferon activity and are increased in patients with systemic lupus erythematosus. Lupus Sci Med 2014;1:e000026. 10.1136/lupus-2014-000026 25452879 PMC4246916

[29] Oliveira JJ, Karrar S, Rainbow DB, et al. The plasma biomarker soluble SIGLEC-1 is associated with the type I interferon transcriptional signature, ethnic background and renal disease in systemic lupus erythematosus. Arthritis Res Ther 2018;20:152. 10.1186/s13075-018-1649-1 30053827 PMC6062988

[30] Olsen NJ, McAloose C, Carter J, et al. Clinical and immunologic profiles in incomplete lupus erythematosus and improvement with hydroxychloroquine treatment. Autoimmune Dis 2016;2016:8791629. 10.1155/2016/8791629 28116147 PMC5225311

[31] van den Hoogen LL, van Roon JAG, Mertens JS, et al. Galectin-9 is an easy to measure biomarker for the interferon signature in systemic lupus erythematosus and antiphospholipid syndrome. Ann Rheum Dis 2018;77:1810–4. 10.1136/annrheumdis-2018-213497 30185417

[32] Wahadat MJ, Bodewes ILA, Maria NI, et al. Type I IFN signature in childhood-onset systemic lupus erythematosus: a conspiracy of DNA- and RNA-sensing receptors? Arthritis Res Ther 2018;20:4. 10.1186/s13075-017-1501-z 29321042 PMC5763828

[33] al-Masri AN, Werfel T, Jakschies D, et al. Intracellular staining of Mx proteins in cells from peripheral blood, bone marrow and skin. Mol Pathol 1997;50:9–14. 10.1136/mp.50.1.9 9208807 PMC379572

[34] Wirestam L, Enocsson H, Skogh T, et al. Interferon-Α coincides with suppressed levels of pentraxin-3 (PTX3) in systemic lupus erythematosus and regulates leucocyte PTX3 in vitro. Clin Exp Immunol 2017;189:83–91. 10.1111/cei.12957 28257596 PMC5461103

[35] Blomberg S, Eloranta ML, Cederblad B, et al. Presence of cutaneous interferon-alpha producing cells in patients with systemic lupus erythematosus. Lupus 2001;10:484–90. 10.1191/096120301678416042 11480846

[36] Mathian A, Mouries-Martin S, Dorgham K, et al. Ultrasensitive serum interferon-α quantification during SLE remission identifies patients at risk for relapse. Ann Rheum Dis 2019;78:1669–76. 10.1136/annrheumdis-2019-215571 31570366

[37] Rodero MP, Decalf J, Bondet V, et al. Detection of interferon alpha protein reveals differential levels and cellular sources in disease. J Exp Med 2017;214:1547–55. 10.1084/jem.20161451 28420733 PMC5413335

[38] Shi SN, Feng SF, Wen YM, et al. Serum interferon in systemic lupus erythematosus. Br J Dermatol 1987;117:155–9. 10.1111/j.1365-2133.1987.tb04111.x 2443158

[39] Kim T, Kanayama Y, Negoro N, et al. Serum levels of interferons in patients with systemic lupus erythematosus. Clin Exp Immunol 1987;70:562–9. doi:2449306 2449306 PMC1542177

[40] Li Y, Lee PY, Kellner ES, et al. Monocyte surface expression of Fcgamma receptor RI (CD64), a biomarker reflecting type-I interferon levels in systemic lupus erythematosus. Arthritis Res Ther 2010;12:R90. 10.1186/ar3017 20478071 PMC2911874

[41] Wilhelm TR, Taddeo A, Winter O, et al. Siglec-1-positive plasmacytoid dendritic cells (pdcs) in human peripheral blood: a semi-mature and myeloid-like subset imbalanced during protective and autoimmune responses. Clin Immunol 2016;163:42–51. 10.1016/j.clim.2015.12.001 26674280

[42] Biesen R, Demir C, Barkhudarova F, et al. Sialic acid-binding Ig-like lectin 1 expression in inflammatory and resident monocytes is a potential biomarker for monitoring disease activity and success of therapy in systemic lupus erythematosus. Arthritis Rheum 2008;58:1136–45. 10.1002/art.23404 18383365

[43] Banchereau R, Hong S, Cantarel B, et al. Personalized immunomonitoring uncovers molecular networks that stratify lupus patients. Cell 2016;165:551–65. 10.1016/j.cell.2016.03.008 27040498 PMC5426482

[44] Flint SM, Jovanovic V, Teo BW, et al. Leucocyte subset-specific type 1 interferon signatures in SLE and other immune-mediated diseases. RMD Open 2016;2:e000183. 10.1136/rmdopen-2015-000183 27252891 PMC4879345

[45] Mackay M, Oswald M, Sanchez-Guerrero J, et al. Molecular signatures in systemic lupus erythematosus: distinction between disease flare and infection. Lupus Sci Med 2016;3:e000159. 10.1136/lupus-2016-000159 27933197 PMC5133406

[46] Olferiev M, Jacek E, Kirou KA, et al. Novel molecular signatures in mononuclear cell populations from patients with systemic lupus erythematosus. Clin Immunol 2016;172:34–43. 10.1016/j.clim.2016.08.018 27576056

[47] Chiche L, Jourde-Chiche N, Whalen E, et al. Modular transcriptional repertoire analyses of adults with systemic lupus erythematosus reveal distinct type I and type II interferon signatures. Arthritis Rheumatol 2014;66:1583–95. 10.1002/art.38628 24644022 PMC4157826

[48] Assassi S, Mayes MD, Arnett FC, et al. Systemic sclerosis and lupus: points in an interferon-mediated continuum. Arthritis Rheum 2010;62:589–98. 10.1002/art.27224 20112391 PMC2879587

[49] Baechler EC, Bauer JW, Slattery CA, et al. An interferon signature in the peripheral blood of dermatomyositis patients is associated with disease activity. Mol Med 2007;13:59–68. 10.2119/2006-00085.Baechler 17515957 PMC1869622

[50] Baechler EC, Batliwalla FM, Karypis G, et al. Interferon-inducible gene expression signature in peripheral blood cells of patients with severe lupus. Proc Natl Acad Sci U S A 2003;100:2610–5. 10.1073/pnas.0337679100 12604793 PMC151388

[51] Becker AM, Dao KH, Han BK, et al. SLE peripheral blood B cell, T cell and myeloid cell transcriptomes display unique profiles and each subset contributes to the interferon signature. PLoS One 2013;8:e67003. 10.1371/journal.pone.0067003 23826184 PMC3691135

[52] Dey-Rao R, Sinha AA. Genome-wide transcriptional profiling of chronic cutaneous lupus erythematosus (CCLE) peripheral blood identifies systemic alterations relevant to the skin manifestation. Genomics 2015;105:90–100. 10.1016/j.ygeno.2014.11.004 25451738

[53] Higgs BW, Liu Z, White B, et al. Patients with systemic lupus erythematosus, myositis, rheumatoid arthritis and scleroderma share activation of a common type I interferon pathway. Ann Rheum Dis 2011;70:2029–36. 10.1136/ard.2011.150326 21803750

[54] Hoffman RW, Merrill JT, Alarcón-Riquelme MME, et al. Gene expression and pharmacodynamic changes in 1,760 systemic lupus erythematosus patients from two phase III trials of BAFF blockade with tabalumab. Arthritis Rheumatol 2017;69:643–54. 10.1002/art.39950 27723281 PMC6585752

[55] Kyogoku C, Smiljanovic B, Grün JR, et al. Cell-specific type I IFN signatures in autoimmunity and viral infection: what makes the difference? PLoS ONE 2013;8:e83776. 10.1371/journal.pone.0083776 24391825 PMC3877094

[56] Lugar PL, Love C, Grammer AC, et al. Molecular characterization of circulating plasma cells in patients with active systemic lupus erythematosus. PLoS One 2012;7:e44362. 10.1371/journal.pone.0044362 23028528 PMC3448624

[57] Ye S, Pang H, Gu Y-Y, et al. Protein interaction for an interferon-inducible systemic lupus associated gene, IFIT1. Rheumatology (Oxford) 2003;42:1155–63. 10.1093/rheumatology/keg315 12777642

[58] Higgs BW, Zhu W, Richman L, et al. Identification of activated cytokine pathways in the blood of systemic lupus erythematosus, myositis, rheumatoid arthritis, and scleroderma patients. Int J Rheum Dis 2012;15:25–35. 10.1111/j.1756-185X.2011.01654.x 22324944

[59] Lauwerys BR, Hachulla E, Spertini F, et al. Down-regulation of interferon signature in systemic lupus erythematosus patients by active immunization with interferon α-kinoid. Arthritis Rheum 2013;65:447–56. 10.1002/art.37785 23203821

[60] Yao Y, Richman L, Higgs BW, et al. Neutralization of interferon-alpha/beta-inducible genes and downstream effect in a phase I trial of an anti-interferon-alpha monoclonal antibody in systemic lupus erythematosus. Arthritis Rheum 2009;60:1785–96. 10.1002/art.24557 19479852

[61] Perez-Sanchez C, Barbarroja N, Messineo S, et al. Gene profiling reveals specific molecular pathways in the pathogenesis of atherosclerosis and cardiovascular disease in antiphospholipid syndrome, systemic lupus erythematosus and antiphospholipid syndrome with lupus. Ann Rheum Dis 2015;74:1441–9. 10.1136/annrheumdis-2013-204600 24618261

[62] Smiljanovic B, Grün JR, Biesen R, et al. The multifaceted balance of TNF-α and type I/II interferon responses in SLE and RA: how monocytes manage the impact of cytokines. J Mol Med (Berl) 2012;90:1295–309. 10.1007/s00109-012-0907-y 22610275

[63] Zhu H, Mi W, Luo H, et al. Whole-genome transcription and DNA methylation analysis of peripheral blood mononuclear cells identified aberrant gene regulation pathways in systemic lupus erythematosus. Arthritis Res Ther 2016;18:162. 10.1186/s13075-016-1050-x 27412348 PMC4942934

[64] Kennedy WP, Maciuca R, Wolslegel K, et al. Association of the interferon signature metric with serological disease manifestations but not global activity scores in multiple cohorts of patients with SLE. Lupus Sci Med 2015;2:e000080. 10.1136/lupus-2014-000080 25861459 PMC4379884

[65] Komatsuda A, Wakui H, Iwamoto K, et al. Up-regulated expression of toll-like receptors mrnas in peripheral blood mononuclear cells from patients with systemic lupus erythematosus. Clin Exp Immunol 2008;152:482–7. 10.1111/j.1365-2249.2008.03646.x 18373699 PMC2453201

[66] Li D, Song L, Fan Y, et al. Down-Regulation of TIPE2 mRNA expression in peripheral blood mononuclear cells from patients with systemic lupus erythematosus. Clin Immunol 2009;133:422–7. 10.1016/j.clim.2009.08.014 19748832

[67] Yuan Y, Ma H, Ye Z, et al. Interferon-Stimulated gene 15 expression in systemic lupus erythematosus. Z Rheumatol 2018;77:256–62. 10.1007/s00393-017-0274-8 28204879

[68] Blokland SLM, van den Hoogen LL, Leijten EFA, et al. Increased expression of Fas on group 2 and 3 innate lymphoid cells is associated with an interferon signature in systemic lupus erythematosus and Sjögren’s syndrome. Rheumatology (Oxford) 2019;58:1740–5. 10.1093/rheumatology/kez116 31220315

[69] Braunstein I, Klein R, Okawa J, et al. The interferon-regulated gene signature is elevated in subacute cutaneous lupus erythematosus and discoid lupus erythematosus and correlates with the cutaneous lupus area and severity index score. Br J Dermatol 2012;166:971–5. 10.1111/j.1365-2133.2012.10825.x 22242767 PMC3336025

[70] Brkic Z, Corneth OBJ, van Helden-Meeuwsen CG, et al. T-Helper 17 cell cytokines and interferon type I: partners in crime in systemic lupus erythematosus? Arthritis Res Ther 2014;16:R62. 10.1186/ar4499 24598455 PMC4060204

[71] de Jong TD, Lübbers J, Turk S, et al. The type I interferon signature in leukocyte subsets from peripheral blood of patients with early arthritis: a major contribution by granulocytes. Arthritis Res Ther 2016;18:165. 10.1186/s13075-016-1065-3 27411379 PMC4944477

[72] Dominguez-Gutierrez PR, Ceribelli A, Satoh M, et al. Elevated signal transducers and activators of transcription 1 correlates with increased C-C motif chemokine ligand 2 and C-X-C motif chemokine 10 levels in peripheral blood of patients with systemic lupus erythematosus. Arthritis Res Ther 2014;16:R20. 10.1186/ar4448 24451065 PMC3978614

[73] Dominguez-Gutierrez PR, Ceribelli A, Satoh M, et al. Positive correlation of STAT1 and miR-146a with anemia in patients with systemic lupus erythematosus. J Clin Immunol 2014;34:171–80. 10.1007/s10875-013-9973-3 24292724 PMC3943692

[74] Ekholm L, Kahlenberg JM, Barbasso Helmers S, et al. Dysfunction of endothelial progenitor cells is associated with the type I IFN pathway in patients with polymyositis and dermatomyositis. Rheumatology (Oxford) 2016;55:1987–92. 10.1093/rheumatology/kew288 27498356 PMC5088625

[75] El-Sherbiny YM, Psarras A, Md Yusof MY, et al. A novel two-score system for interferon status segregates autoimmune diseases and correlates with clinical features. Sci Rep 2018;8:5793. 10.1038/s41598-018-24198-1 29643425 PMC5895784

[76] Feng X, Chen W, Xiao L, et al. Artesunate inhibits type I interferon-induced production of macrophage migration inhibitory factor in patients with systemic lupus erythematosus. Lupus 2017;26:62–72. 10.1177/0961203316651738 27230555 PMC5124426

[77] Feng X, Huang J, Liu Y, et al. Identification of interferon-inducible genes as diagnostic biomarker for systemic lupus erythematosus. Clin Rheumatol 2015;34:71–9. 10.1007/s10067-014-2799-4 25344775

[78] Fu Q, Chen X, Cui H, et al. Association of elevated transcript levels of interferon-inducible chemokines with disease activity and organ damage in systemic lupus erythematosus patients. Arthritis Res Ther 2008;10:R112. 10.1186/ar2510 18793417 PMC2592795

[79] Furie R, Werth VP, Merola JF, et al. Monoclonal antibody targeting BDCA2 ameliorates skin lesions in systemic lupus erythematosus. J Clin Invest 2019;129:1359–71. 10.1172/JCI124466 30645203 PMC6391094

[80] Jin Z, Fan W, Jensen MA, et al. Single-cell gene expression patterns in lupus monocytes independently indicate disease activity, interferon and therapy. Lupus Sci Med 2017;4:e000202. 10.1136/lupus-2016-000202 29238602 PMC5724340

[81] Landolt-Marticorena C, Bonventi G, Lubovich A, et al. Lack of association between the interferon-alpha signature and longitudinal changes in disease activity in systemic lupus erythematosus. Ann Rheum Dis 2009;68:1440–6. 10.1136/ard.2008.093146 18772188

[82] Liu M, Liu J, Hao S, et al. Higher activation of the interferon-gamma signaling pathway in systemic lupus erythematosus patients with a high type I IFN score: relation to disease activity. Clin Rheumatol 2018;37:2675–84. 10.1007/s10067-018-4138-7 29774490

[83] Md Yusof MY, Psarras A, El-Sherbiny YM, et al. Prediction of autoimmune connective tissue disease in an at-risk cohort: prognostic value of a novel two-score system for interferon status. Ann Rheum Dis 2018;77:1432–9. 10.1136/annrheumdis-2018-213386 29929956 PMC6161671

[84] Reynolds JA, Briggs TA, Rice GI, et al. Type I interferon in patients with systemic autoimmune rheumatic disease is associated with haematological abnormalities and specific autoantibody profiles. Arthritis Res Ther 2019;21:147. 10.1186/s13075-019-1929-4 31200750 PMC6567906

[85] Rodríguez-Carrio J, López P, Alperi-López M, et al. Irf4 and irgs delineate clinically relevant gene expression signatures in systemic lupus erythematosus and rheumatoid arthritis. Front Immunol 2018;9:3085. 10.3389/fimmu.2018.03085 30666255 PMC6330328

[86] Tang J, Gu Y, Zhang M, et al. Increased expression of the type I interferon-inducible gene, lymphocyte antigen 6 complex locus E, in peripheral blood cells is predictive of lupus activity in a large cohort of Chinese lupus patients. Lupus 2008;17:805–13. 10.1177/0961203308089694 18755862

[87] Tydén H, Lood C, Gullstrand B, et al. Endothelial dysfunction is associated with activation of the type I interferon system and platelets in patients with systemic lupus erythematosus. RMD Open 2017;3:e000508. 10.1136/rmdopen-2017-000508 29119007 PMC5663269

[88] van den Hoogen LL, Fritsch-Stork RDE, Versnel MA, et al. Monocyte type I interferon signature in antiphospholipid syndrome is related to proinflammatory monocyte subsets, hydroxychloroquine and statin use. Ann Rheum Dis 2016;75:e81. 10.1136/annrheumdis-2016-210485 27689737

[89] Bodewes ILA, Huijser E, van Helden-Meeuwsen CG, et al. TBK1: a key regulator and potential treatment target for interferon positive sjögren’s syndrome, systemic lupus erythematosus and systemic sclerosis. J Autoimmun 2018;91:97–102. 10.1016/j.jaut.2018.02.001 29673738

[90] Casey KA, Guo X, Smith MA, et al. Type I interferon receptor blockade with anifrolumab corrects innate and adaptive immune perturbations of SLE. Lupus Sci Med 2018;5:e000286. 10.1136/lupus-2018-000286 30538817 PMC6257383

[91] Dominguez-Gutierrez PR, Ceribelli A, Satoh M, et al. Reduced levels of CCL2 and CXCL10 in systemic lupus erythematosus patients under treatment with prednisone, mycophenolate mofetil, or hydroxychloroquine, except in a high STAT1 subset. Arthritis Res Ther 2014;16:R23. 10.1186/ar4451 24460726 PMC3978465

[92] Feng X, Wu H, Grossman JM, et al. Association of increased interferon-inducible gene expression with disease activity and lupus nephritis in patients with systemic lupus erythematosus. Arthritis Rheum 2006;54:2951–62. 10.1002/art.22044 16947629

[93] Kirou KA, Lee C, George S, et al. Activation of the interferon-alpha pathway identifies a subgroup of systemic lupus erythematosus patients with distinct serologic features and active disease. Arthritis Rheum 2005;52:1491–503. 10.1002/art.21031 15880830

[94] Lee PY, Li Y, Richards HB, et al. Type I interferon as a novel risk factor for endothelial progenitor cell depletion and endothelial dysfunction in systemic lupus erythematosus. Arthritis Rheum 2007;56:3759–69. 10.1002/art.23035 17968925

[95] Li Q-Z, Zhou J, Lian Y, et al. Interferon signature gene expression is correlated with autoantibody profiles in patients with incomplete lupus syndromes. Clin Exp Immunol 2010;159:281–91. 10.1111/j.1365-2249.2009.04057.x 19968664 PMC2819494

[96] Kellner ES, Lee PY, Li Y, et al. Endogenous type-I interferon activity is not associated with depression or fatigue in systemic lupus erythematosus. J Neuroimmunol 2010;223:13–9. 10.1016/j.jneuroim.2010.03.018 20416954 PMC3580233

[97] Wither J, Johnson SR, Liu T, et al. Presence of an interferon signature in individuals who are anti-nuclear antibody positive lacking a systemic autoimmune rheumatic disease diagnosis. Arthritis Res Ther 2017;19:41. 10.1186/s13075-017-1243-y 28245862 PMC5331647

[98] Coit P, Jeffries M, Altorok N, et al. Genome-wide DNA methylation study suggests epigenetic accessibility and transcriptional poising of interferon-regulated genes in naïve CD4+ T cells from lupus patients. J Autoimmun 2013;43:78–84. 10.1016/j.jaut.2013.04.003 23623029 PMC3790645

[99] Joseph S, George NI, Green-Knox B, et al. Epigenome-wide association study of peripheral blood mononuclear cells in systemic lupus erythematosus: identifying DNA methylation signatures associated with interferon-related genes based on ethnicity and SLEDAI. J Autoimmun 2019;96:147–57. 10.1016/j.jaut.2018.09.007 30301579

[100] Yeung KS, Chung BH-Y, Choufani S, et al. Genome-wide DNA methylation analysis of chinese patients with systemic lupus erythematosus identified hypomethylation in genes related to the type I interferon pathway. PLoS One 2017;12:e0169553. 10.1371/journal.pone.0169553 28085900 PMC5234836

[101] Absher DM, Li X, Waite LL, et al. Genome-wide DNA methylation analysis of systemic lupus erythematosus reveals persistent hypomethylation of interferon genes and compositional changes to CD4+ T-cell populations. PLoS Genet 2013;9:e1003678. 10.1371/journal.pgen.1003678 23950730 PMC3738443

[102] Coit P, Yalavarthi S, Ognenovski M, et al. Epigenome profiling reveals significant DNA demethylation of interferon signature genes in lupus neutrophils. J Autoimmun 2015;58:59–66. 10.1016/j.jaut.2015.01.004 25638528 PMC4363276

[103] Imgenberg-Kreuz J, Almlöf JC, Leonard D, et al. Shared and unique patterns of DNA methylation in systemic lupus erythematosus and primary sjögren’s syndrome. Front Immunol 2019;10:1686. 10.3389/fimmu.2019.01686 31428085 PMC6688520

[104] Ulff-Møller CJ, Asmar F, Liu Y, et al. Twin DNA methylation profiling reveals flare-dependent interferon signature and B cell promoter hypermethylation in systemic lupus erythematosus. Arthritis Rheumatol 2018;70:878–90. 10.1002/art.40422 29361205

[105] Weeding E, Coit P, Yalavarthi S, et al. Genome-wide DNA methylation analysis in primary antiphospholipid syndrome neutrophils. Clin Immunol 2018;196:110–6. 10.1016/j.clim.2018.11.011 30471352 PMC6413498

[106] Zhao M, Zhou Y, Zhu B, et al. IFI44L promoter methylation as a blood biomarker for systemic lupus erythematosus. Ann Rheum Dis 2016;75:1998–2006. 10.1136/annrheumdis-2015-208410 26787370 PMC4955646

[107] Andrade D, Kim M, Blanco LP, et al. Interferon-Α and angiogenic dysregulation in pregnant lupus patients who develop preeclampsia. Arthritis Rheumatol 2015;67:977–87. 10.1002/art.39029 25603823 PMC4380868

[108] Hua J, Kirou K, Lee C, et al. Functional assay of type I interferon in systemic lupus erythematosus plasma and association with anti-RNA binding protein autoantibodies. Arthritis Rheum 2006;54:1906–16. 10.1002/art.21890 16736505

[109] Somers EC, Zhao W, Lewis EE, et al. Type I interferons are associated with subclinical markers of cardiovascular disease in a cohort of systemic lupus erythematosus patients. PLoS One 2012;7:e37000. 10.1371/journal.pone.0037000 22606325 PMC3351452

[110] Niewold TB, Rivera TL, Buyon JP, et al. Serum type I interferon activity is dependent on maternal diagnosis in anti-SSA/ro-positive mothers of children with neonatal lupus. Arthritis Rheum 2008;58:541–6. 10.1002/art.23191 18240214 PMC2755051

[111] Weckerle CE, Mangale D, Franek BS, et al. Large-Scale analysis of tumor necrosis factor α levels in systemic lupus erythematosus. Arthritis Rheum 2012;64:2947–52. 10.1002/art.34483 22488302 PMC3396783

[112] Niewold TB, Hua J, Lehman TJA, et al. High serum IFN-alpha activity is a heritable risk factor for systemic lupus erythematosus. Genes Immun 2007;8:492–502. 10.1038/sj.gene.6364408 17581626 PMC2702174

[113] Kirou KA, Lee C, George S, et al. Coordinate overexpression of interferon-alpha-induced genes in systemic lupus erythematosus. Arthritis Rheum 2004;50:3958–67. 10.1002/art.20798 15593221

[114] Biswas PS, Pawaria S, Maers K, et al. Complement component c5a permits the co-existence of pathogenic th17 cells and type I interferon in lupus. Cytokine 2013;63:248. 10.1016/j.cyto.2013.06.026

[115] Dall’era MC, Cardarelli PM, Preston BT, et al. Type I interferon correlates with serological and clinical manifestations of SLE. Ann Rheum Dis 2005;64:1692–7. 10.1136/ard.2004.033753 15843451 PMC1755300

[116] Kato Y, Park J, Takamatsu H, et al. Apoptosis-derived membrane vesicles drive the cGAS-STING pathway and enhance type I IFN production in systemic lupus erythematosus. Ann Rheum Dis 2018;77:1507–15. 10.1136/annrheumdis-2018-212988 29945921 PMC6161667

[117] Lackovic V, Borecký L, Rovenský J, et al. Periodicity of interferon appearance in serum of patients with systemic lupus erythematosus. Arthritis Rheum 1984;27:597–8. 10.1002/art.1780270523 6202304

[118] Preble OT, Black RJ, Friedman RM, et al. Systemic lupus erythematosus: presence in human serum of an unusual acid-labile leukocyte interferon. Science 1982;216:429–31. 10.1126/science.6176024 6176024

[119] Hervier B, Beziat V, Haroche J, et al. Phenotype and function of natural killer cells in systemic lupus erythematosus: excess interferon-γ production in patients with active disease. Arthritis Rheum 2011;63:1698–706. 10.1002/art.30313 21370226

[120] Hooks JJ, Moutsopoulos HM, Geis SA, et al. Immune interferon in the circulation of patients with autoimmune disease. N Engl J Med 1979;301:5–8. 10.1056/NEJM197907053010102 449915

[121] Cesario TC, Andrews BS, Martin DA, et al. Interferon in synovial fluid and serum of patients with rheumatic disease. J Rheumatol 1983;10:647–50. 6194296

[122] Hooks JJ, Jordan GW, Cupps T, et al. Multiple interferons in the circulation of patients with systemic lupus erythematosus and vasculitis. Arthritis Rheum 1982;25:396–400. 10.1002/art.1780250406 6176247

[123] Ytterberg SR, Schnitzer TJ. Serum interferon levels in patients with systemic lupus erythematosus. Arthritis Rheum 1982;25:401–6. 10.1002/art.1780250407 6176248

[124] Weix J, Häupl T, Raio L, et al. The physiologic increase in expression of some type I IFN-inducible genes during pregnancy is not associated with improved disease activity in pregnant patients with rheumatoid arthritis. Transl Res 2013;161:505–12. 10.1016/j.trsl.2013.02.007 23507374

[125] Rodríguez-Carrio J, de Paz B, López P, et al. IFNα serum levels are associated with endothelial progenitor cells imbalance and disease features in rheumatoid arthritis patients. PLoS One 2014;9:e86069. 10.1371/journal.pone.0086069 24465874 PMC3897639

[126] Lepore L, Pennesi M, Saletta S, et al. Study of IL-2, IL-6, TNF alpha, IFN gamma and beta in the serum and synovial fluid of patients with juvenile chronic arthritis. Clin Exp Rheumatol 1994;12:561–5. 7531125

[127] Båve U, Nordmark G, Lövgren T, et al. Activation of the type I interferon system in primary Sjögren’s syndrome: a possible etiopathogenic mechanism. Arthritis Rheum 2005;52:1185–95. 10.1002/art.20998 15818675

[128] Fong KY, Boey ML, Koh WH, et al. Cytokine concentrations in the synovial fluid and plasma of rheumatoid arthritis patients: correlation with bony erosions. Clin Exp Rheumatol 1994;12:55–8. 8162643

[129] Shiozawa S, Chihara K, Shiozawa K, et al. A sensitive radioimmunoassay for alpha-interferon: circulating alpha-interferon-like substance in the plasma of healthy individuals and rheumatoid arthritis patients. Clin Exp Immunol 1986;66:77–87. 3802576 PMC1542641

[130] Bennett L, Palucka AK, Arce E, et al. Interferon and granulopoiesis signatures in systemic lupus erythematosus blood. J Exp Med 2003;197:711–23. 10.1084/jem.20021553 12642603 PMC2193846

[131] Reynier F, Petit F, Paye M, et al. Importance of correlation between gene expression levels: application to the type I interferon signature in rheumatoid arthritis. PLoS One 2011;6:e24828. 10.1371/journal.pone.0024828 22043277 PMC3197194

[132] van der Pouw Kraan TCTM, Wijbrandts CA, van Baarsen LGM, et al. Rheumatoid arthritis subtypes identified by genomic profiling of peripheral blood cells: assignment of a type I interferon signature in a subpopulation of patients. Ann Rheum Dis 2007;66:1008–14. 10.1136/ard.2006.063412 17223656 PMC1954704

[133] Li Q-Z, Karp DR, Quan J, et al. Risk factors for ANA positivity in healthy persons. Arthritis Res Ther 2011;13:R38. 10.1186/ar3271 21366908 PMC3132017

[134] Castañeda-Delgado JE, Bastián-Hernandez Y, Macias-Segura N, et al. Type I interferon gene response is increased in early and established rheumatoid arthritis and correlates with autoantibody production. Front Immunol 2017;8:285. 10.3389/fimmu.2017.00285 28373872 PMC5357778

[135] de Jong TD, Vosslamber S, Mantel E, et al. Physiological evidence for diversification of ifnα- and ifnβ-mediated response programs in different autoimmune diseases. Arthritis Res Ther 2016;18:49. 10.1186/s13075-016-0946-9 26882897 PMC4756531

[136] Rodríguez-Carrio J, Alperi-López M, López P, et al. Heterogeneity of the type I interferon signature in rheumatoid arthritis: A potential limitation for its use as A clinical biomarker. Front Immunol 2017;8:2007. 10.3389/fimmu.2017.02007 29387065 PMC5775969

[137] Cooles FAH, Anderson AE, Lendrem DW, et al. The interferon gene signature is increased in patients with early treatment-naive rheumatoid arthritis and predicts a poorer response to initial therapy. J Allergy Clin Immunol 2018;141:445–8. 10.1016/j.jaci.2017.08.026 28987811 PMC5751729

[138] Wright HL, Thomas HB, Moots RJ, et al. Interferon gene expression signature in rheumatoid arthritis neutrophils correlates with a good response to tnfi therapy. Rheumatology (Oxford) 2015;54:188–93. 10.1093/rheumatology/keu299 25125592

[139] Mavragani CP, La DT, Stohl W, et al. Association of the response to tumor necrosis factor antagonists with plasma type I interferon activity and interferon-beta/alpha ratios in rheumatoid arthritis patients: A post hoc analysis of A predominantly hispanic cohort. Arthritis Rheum 2010;62:392–401. 10.1002/art.27226 20112385 PMC2821991

[140] Hertzog PJ, Emery P, Cheetham BF, et al. Interferons in rheumatoid arthritis: alterations in production and response related to disease activity. Clin Immunol Immunopathol 1988;48:192–201. 10.1016/0090-1229(88)90083-9 2455616

[141] Arvin AM, Miller JJ. Acid labile alpha-interferon in sera and synovial fluids from patients with juvenile arthritis. Arthritis Rheum 1984;27:582–5. 10.1002/art.1780270517 6721889

[142] Pollard RPE, Abdulahad WH, Bootsma H, et al. Predominantly proinflammatory cytokines decrease after B cell depletion therapy in patients with primary Sjogren’s syndrome. Ann Rheum Dis 2013;72:2048–50. 10.1136/annrheumdis-2013-203447 23864239

[143] Zheng L, Zhang Z, Yu C, et al. Association between IFN-alpha and primary Sjogren’s syndrome. Oral Surg Oral Med Oral Pathol Oral Radiol Endod 2009;107:e12–8. 10.1016/j.tripleo.2008.09.015 19101478

[144] Imgenberg-Kreuz J, Sandling JK, Björk A, et al. Transcription profiling of peripheral B cells in antibody-positive primary Sjögren’s syndrome reveals upregulated expression of CX3CR1 and a type I and type II interferon signature. Scand J Immunol 2018;87:e12662. 10.1111/sji.12662 29655283

[145] Wildenberg ME, van Helden-Meeuwsen CG, van de Merwe JP, et al. Systemic increase in type I interferon activity in Sjögren’s syndrome: a putative role for plasmacytoid dendritic cells. Eur J Immunol 2008;38:2024–33. 10.1002/eji.200738008 18581327

[146] Alunno A, Caneparo V, Bistoni O, et al. FRI0024 Interferon gamma-inducible protein 16 (IFI16) in rheumatoid arthritis: a novel biomarker for pulmonary involvement? Ann Rheum Dis 2015;74:427. 10.1136/annrheumdis-2015-eular.1811

[147] Maria NI, Brkic Z, Waris M, et al. Mxa as a clinically applicable biomarker for identifying systemic interferon type I in primary Sjogren’s syndrome. Ann Rheum Dis 2014;73:1052–9. 10.1136/annrheumdis-2012-202552 23831963 PMC4033122

[148] Rose T, Szelinski F, Lisney A, et al. SIGLEC1 is a biomarker of disease activity and indicates extraglandular manifestation in primary Sjögren’s syndrome. RMD Open 2016;2:e000292. 10.1136/rmdopen-2016-000292 28123773 PMC5237743

[149] Shiozawa S, Shiozawa K, Shimizu S, et al. Immunoreactive circulating alpha-interferon is low in Sjögren’s syndrome. Br J Rheumatol 1990;29:50–2. 10.1093/rheumatology/29.1.50 2306573

[150] Emamian ES, Leon JM, Lessard CJ, et al. Peripheral blood gene expression profiling in Sjögren’s syndrome. Genes Immun 2009;10:285–96. 10.1038/gene.2009.20 19404300 PMC3273959

[151] Kimoto O, Sawada J, Shimoyama K, et al. Activation of the interferon pathway in peripheral blood of patients with Sjogren’s syndrome. J Rheumatol 2011;38:310–6. 10.3899/jrheum.100486 21078725

[152] Bodewes ILA, Gottenberg J-E, van Helden-Meeuwsen CG, et al. Hydroxychloroquine treatment downregulates systemic interferon activation in primary Sjögren’s syndrome in the JOQUER randomized trial. Rheumatology (Oxford) 2020;59:107–11. 10.1093/rheumatology/kez242 31237947 PMC6909893

[153] Brkic Z, Maria NI, van Helden-Meeuwsen CG, et al. Prevalence of interferon type I signature in CD14 monocytes of patients with Sjogren’s syndrome and association with disease activity and BAFF gene expression. Ann Rheum Dis 2013;72:728–35. 10.1136/annrheumdis-2012-201381 22736090 PMC3618683

[154] Davies R, Sarkar I, Hammenfors D, et al. Single cell based phosphorylation profiling identifies alterations in toll-like receptor 7 and 9 signaling in patients with primary sjögren’s syndrome. Front Immunol 2019;10:281. 10.3389/fimmu.2019.00281 30846988 PMC6393381

[155] Nezos A, Gravani F, Tassidou A, et al. Type I and II interferon signatures in sjogren’s syndrome pathogenesis: contributions in distinct clinical phenotypes and sjogren’s related lymphomagenesis. J Autoimmun 2015;63:47–58. 10.1016/j.jaut.2015.07.002 26183766 PMC4564326

[156] Altorok N, Coit P, Hughes T, et al. Genome-Wide DNA methylation patterns in naive CD4+ T cells from patients with primary Sjögren’s syndrome. Arthritis Rheumatol 2014;66:731–9. 10.1002/art.38264 24574234 PMC4009982

[157] Mavragani CP, Niewold TB, Moutsopoulos NM, et al. Augmented interferon-alpha pathway activation in patients with Sjögren’s syndrome treated with etanercept. Arthritis Rheum 2007;56:3995–4004. 10.1002/art.23062 18050196 PMC2737264

[158] Eloranta M-L, Franck-Larsson K, Lövgren T, et al. Type I interferon system activation and association with disease manifestations in systemic sclerosis. Ann Rheum Dis 2010;69:1396–402. 10.1136/ard.2009.121400 20472592

[159] Guo X, Higgs BW, Bay-Jensen AC, et al. Suppression of T cell activation and collagen accumulation by an anti-IFNAR1 mab, anifrolumab, in adult patients with systemic sclerosis. J Invest Dermatol 2015;135:2402–9. 10.1038/jid.2015.188 25993119

[160] Mariotti B, Servaas NH, Rossato M, et al. The long non-coding RNA NRIR drives IFN-response in monocytes: implication for systemic sclerosis. Front Immunol 2019;10:100. 10.3389/fimmu.2019.00100 30804934 PMC6371048

[161] Liu X, Mayes MD, Tan FK, et al. Correlation of interferon-inducible chemokine plasma levels with disease severity in systemic sclerosis. Arthritis Rheum 2013;65:226–35. 10.1002/art.37742 23055137 PMC3687352

[162] York MR, Nagai T, Mangini AJ, et al. A macrophage marker, siglec-1, is increased on circulating monocytes in patients with systemic sclerosis and induced by type i interferons and toll-like receptor agonists. Arthritis Rheum 2007;56:1010–20. 10.1002/art.22382 17328080

[163] Assassi S, Wang X, Chen G, et al. Myeloablation followed by autologous stem cell transplantation normalises systemic sclerosis molecular signatures. Ann Rheum Dis 2019;78:1371–8. 10.1136/annrheumdis-2019-215770 31391177 PMC7167108

[164] Bos CL, van Baarsen LGM, Timmer TCG, et al. Molecular subtypes of systemic sclerosis in association with anti-centromere antibodies and digital ulcers. Genes Immun 2009;10:210–8. 10.1038/gene.2008.98 19129850

[165] Tan FK, Zhou X, Mayes MD, et al. Signatures of differentially regulated interferon gene expression and vasculotrophism in the peripheral blood cells of systemic sclerosis patients. Rheumatology (Oxford) 2006;45:694–702. 10.1093/rheumatology/kei244 16418202

[166] Christmann RB, Hayes E, Pendergrass S, et al. Interferon and alternative activation of monocyte/macrophages in systemic sclerosis-associated pulmonary arterial hypertension. Arthritis Rheum 2011;63:1718–28. 10.1002/art.30318 21425123 PMC4030759

[167] Airò P, Ghidini C, Zanotti C, et al. Upregulation of myxovirus-resistance protein A: a possible marker of type I interferon induction in systemic sclerosis. J Rheumatol 2008;35:2192–200. 10.3899/jrheum.080418 18843779

[168] de Oliveira DB, Almeida GM de F, Guedes ACM, et al. Basal activation of type I interferons (alpha2 and beta) and 2’ ’'OAS genes: insights into differential expression profiles of interferon system components in systemic sclerosis. Int J Rheumatol 2011;2011:275617. 10.1155/2011/275617 22121373 PMC3206376

[169] Brkic Z, van Bon L, Cossu M, et al. The interferon type I signature is present in systemic sclerosis before overt fibrosis and might contribute to its pathogenesis through high BAFF gene expression and high collagen synthesis. Ann Rheum Dis 2016;75:1567–73. 10.1136/annrheumdis-2015-207392 26371289

[170] Wang B, Higgs BW, Chang L, et al. Pharmacogenomics and translational simulations to bridge indications for an anti-interferon-α receptor antibody. Clin Pharmacol Ther 2013;93:483–92. 10.1038/clpt.2013.35 23511714

[171] Ding W, Pu W, Wang L, et al. Genome-wide DNA methylation analysis in systemic sclerosis reveals hypomethylation of IFN-associated genes in CD4^+^ and CD8^+^ T cells. J Invest Dermatol 2018;138:1069–77. 10.1016/j.jid.2017.12.003 29248544

[172] Wuttge DM, Lood C, Tufvesson E, et al. Increased serum type I interferon activity in early systemic sclerosis patients is associated with antibodies against Sjögren’s syndrome antigens and nuclear ribonucleoprotein antigens. Scand J Rheumatol 2013;42:235–40. 10.3109/03009742.2012.736532 23379597

[173] Sun WC, Sun YC, Lin H, et al. Dysregulation of the type I interferon system in adult-onset clinically amyopathic dermatomyositis has a potential contribution to the development of interstitial lung disease. Br J Dermatol 2012;167:1236–44. 10.1111/j.1365-2133.2012.11145.x 23013528

[174] Liao AP, Salajegheh M, Nazareno R, et al. Interferon β is associated with type 1 interferon-inducible gene expression in dermatomyositis. Ann Rheum Dis 2011;70:831–6. 10.1136/ard.2010.139949 21177291

[175] Horai Y, Koga T, Fujikawa K, et al. Serum interferon-α is a useful biomarker in patients with anti-melanoma differentiation-associated gene 5 (MDA5) antibody-positive dermatomyositis. Mod Rheumatol 2015;25:85–9. 10.3109/14397595.2014.900843 24716595

[176] Greenberg SA, Higgs BW, Morehouse C, et al. Relationship between disease activity and type 1 interferon- and other cytokine-inducible gene expression in blood in dermatomyositis and polymyositis. Genes Immun 2012;13:207–13. 10.1038/gene.2011.61 21881594

[177] Walsh RJ, Kong SW, Yao Y, et al. Type I interferon-inducible gene expression in blood is present and reflects disease activity in dermatomyositis and polymyositis. Arthritis Rheum 2007;56:3784–92. 10.1002/art.22928 17968926 PMC2443782

[178] O’Connor KA, Abbott KA, Sabin B, et al. Mxa gene expression in juvenile dermatomyositis peripheral blood mononuclear cells: association with muscle involvement. Clin Immunol 2006;120:319–25. 10.1016/j.clim.2006.05.011 16859997 PMC3163162

[179] Bilgic H, Ytterberg SR, Amin S, et al. Interleukin-6 and type I interferon-regulated genes and chemokines mark disease activity in dermatomyositis. Arthritis Rheum 2009;60:3436–46. 10.1002/art.24936 19877033

[180] Piper CJM, Wilkinson MGLl, Deakin CT, et al. CD19+CD24hiCD38hi B cells are expanded in juvenile dermatomyositis and exhibit a pro-inflammatory phenotype after activation through toll-like receptor 7 and interferon-α. Front Immunol 2018;9:1372. 10.3389/fimmu.2018.01372 29988398 PMC6024011

[181] Niewold TB, Kariuki SN, Morgan GA, et al. Elevated serum interferon-alpha activity in juvenile dermatomyositis: associations with disease activity at diagnosis and after thirty-six months of therapy. Arthritis Rheum 2009;60:1815–24. 10.1002/art.24555 19479879 PMC2697261

[182] Balboni I, Niewold TB, Morgan G, et al. Interferon-Α induction and detection of anti-Ro, Anti-La, anti-Sm, and anti-RNP autoantibodies by autoantigen microarray analysis in juvenile dermatomyositis. Arthritis Rheum 2013;65:2424–9. 10.1002/art.38038 23740815 PMC4169271

[183] Ekholm L, Vosslamber S, Tjärnlund A, et al. Autoantibody specificities and type I interferon pathway activation in idiopathic inflammatory myopathies. Scand J Immunol 2016;84:100–9. 10.1111/sji.12449 27173897

[184] Grenn RC, Yalavarthi S, Gandhi AA, et al. Endothelial progenitor dysfunction associates with a type I interferon signature in primary antiphospholipid syndrome. Ann Rheum Dis 2017;76:450–7. 10.1136/annrheumdis-2016-209442 27432357 PMC5233467

[185] Palli E, Kravvariti E, Tektonidou MG. Type I interferon signature in primary antiphospholipid syndrome: clinical and laboratory associations. Front Immunol 2019;10:487. 10.3389/fimmu.2019.00487 30930907 PMC6428719

[186] Ugolini-Lopes MR, Torrezan GT, Gândara APR, et al. Enhanced type I interferon gene signature in primary antiphospholipid syndrome: association with earlier disease onset and preeclampsia. Autoimmun Rev 2019;18:393–8. 10.1016/j.autrev.2018.11.004 30772492

[187] Puccetti A, Fiore PF, Pelosi A, et al. Gene expression profiling in behcet’s disease indicates an autoimmune component in the pathogenesis of the disease and opens new avenues for targeted therapy. J Immunol Res 2018;2018:4246965. 10.1155/2018/4246965 29850627 PMC5941805

[188] Pay S, Simsek I, Erdem H, et al. Dendritic cell subsets and type I interferon system in behçet’s disease: does functional abnormality in plasmacytoid dendritic cells contribute to th1 polarization? Clin Exp Rheumatol 2007;25:S34–40. 17949549

[189] Yilmaz S, Cinar M, Pekel A, et al. The expression of transmembrane and soluble CXCL16 and the relation with interferon-alpha secretion in patients with behçet’s disease. Clin Exp Rheumatol 2013;31:84–7. 24064021

[190] Lee MT, Hooper LC, Kump L, et al. Interferon-Beta and adhesion molecules (E-selectin and s-intracellular adhesion molecule-1) are detected in sera from patients with retinal vasculitis and are induced in retinal vascular endothelial cells by Toll-like receptor 3 signalling. Clin Exp Immunol 2007;147:71–80. 10.1111/j.1365-2249.2006.03253.x 17177965 PMC1810441

[191] Luan JJ, Xing GQ. Pathogenesis of antimicrobial peptides LL-37 and cpg-ODN in ANCA associated vasculitis. J Nephrol 2017;30:63–71. 10.1007/s40620-016-0336-z 27476166

[192] Zhang Y, Shi W, Tang S, et al. The influence of cathelicidin LL37 in human anti-neutrophils cytoplasmic antibody (ANCA) -associated vasculitis. Arthritis Res Ther 2013;15:R161. 10.1186/ar4344 24286516 PMC3979017

[193] Xu Z, Wang X, Zheng Y. Screening for key genes and transcription factors in ankylosing spondylitis by RNA-seq. Exp Ther Med 2018;15:1394–402. 10.3892/etm.2017.5556 29434723 PMC5774495

[194] De Andrea M, De Santis M, Caneparo V, et al. Serum IFI16 and anti-IFI16 antibodies in psoriatic arthritis. Clin Exp Immunol 2020;199:88–96. 10.1111/cei.13376 31571199 PMC6904656

[195] Yamamoto M, Takano K, Kamekura R, et al. Stage classification of IgG4-related Dacryoadenitis and sialadenitis by the serum cytokine environment. Mod Rheumatol 2018;28:1004–8. 10.1080/14397595.2018.1436029 29385874

[196] Bengtsson AA, Sturfelt G, Truedsson L, et al. Activation of type I interferon system in systemic lupus erythematosus correlates with disease activity but not with antiretroviral antibodies. Lupus 2000;9:664–71. 10.1191/096120300674499064 11199920

[197] Jönsen A, Bengtsson AA, Nived O, et al. The heterogeneity of neuropsychiatric systemic lupus erythematosus is reflected in lack of association with cerebrospinal fluid cytokine profiles. Lupus 2003;12:846–50. 10.1191/0961203303lu472sr 14667101

[198] Rose T, Grützkau A, Hirseland H, et al. Ifnα and its response proteins, IP-10 and SIGLEC-1, are biomarkers of disease activity in systemic lupus erythematosus. Ann Rheum Dis 2013;72:1639–45. 10.1136/annrheumdis-2012-201586 23117242

[199] Rose T, Grützkau A, Klotsche J, et al. Are interferon-related biomarkers advantageous for monitoring disease activity in systemic lupus erythematosus? A longitudinal benchmark study. Rheumatology (Oxford) 2017;56:1618–26. 10.1093/rheumatology/kex220 28859328

[200] Schneider L, Colar da Silva AC, Werres Junior LC, et al. Vitamin D levels and cytokine profiles in patients with systemic lupus erythematosus. Lupus 2015;24:1191–7. 10.1177/0961203315584811 25926056

[201] Oke V, Gunnarsson I, Dorschner J, et al. High levels of circulating interferons type I, type II and type III associate with distinct clinical features of active systemic lupus erythematosus. Arthritis Res Ther 2019;21:107. 10.1186/s13075-019-1878-y 31036046 PMC6489203

[202] Munroe ME, Vista ES, Merrill JT, et al. Pathways of impending disease flare in african-american systemic lupus erythematosus patients. J Autoimmun 2017;78:70–8. 10.1016/j.jaut.2016.12.005 28162788 PMC5340190

[203] Bauer JW, Baechler EC, Petri M, et al. Elevated serum levels of interferon-regulated chemokines are biomarkers for active human systemic lupus erythematosus. PLoS Med 2006;3:e491. 10.1371/journal.pmed.0030491 17177599 PMC1702557

[204] Bauer JW, Petri M, Batliwalla FM, et al. Interferon-Regulated chemokines as biomarkers of systemic lupus erythematosus disease activity: a validation study. Arthritis Rheum 2009;60:3098–107. 10.1002/art.24803 19790071 PMC2842939

[205] Connelly KL, Kandane-Rathnayake R, Hoi A, et al. Association of MIF, but not type I interferon-induced chemokines, with increased disease activity in asian patients with systemic lupus erythematosus. Sci Rep 2016;6:29909. 10.1038/srep29909 27453287 PMC4958969

[206] Connelly KL, Kandane-Rathnayake R, Huq M, et al. Longitudinal association of type 1 interferon-induced chemokines with disease activity in systemic lupus erythematosus. Sci Rep 2018;8:3268. 10.1038/s41598-018-20203-9 29459655 PMC5818532

[207] Lee J-R, Haddon DJ, Wand HE, et al. Multiplex giant magnetoresistive biosensor microarrays identify interferon-associated autoantibodies in systemic lupus erythematosus. Sci Rep 2016;6:27623. 10.1038/srep27623 27279139 PMC4899742

[208] Kawasaki M, Fujishiro M, Yamaguchi A, et al. Possible role of the JAK/STAT pathways in the regulation of T cell-interferon related genes in systemic lupus erythematosus. Lupus 2011;20:1231–9. 10.1177/0961203311409963 21980035

[209] Nikpour M, Dempsey AA, Urowitz MB, et al. Association of a gene expression profile from whole blood with disease activity in systemic lupus erythaematosus. Ann Rheum Dis 2008;67:1069–75. 10.1136/ard.2007.074765 18063674

[210] Sharma S, Jin Z, Rosenzweig E, et al. Widely divergent transcriptional patterns between SLE patients of different ancestral backgrounds in sorted immune cell populations. J Autoimmun 2015;60:51–8. 10.1016/j.jaut.2015.04.002 25921064 PMC4457613

[211] Merrill JT, Immermann F, Whitley M, et al. The biomarkers of lupus disease study: A bold approach may mitigate interference of background immunosuppressants in clinical trials. Arthritis Rheumatol 2017;69:1257–66. 10.1002/art.40086 28257602 PMC5501389

[212] Rich SA, Owens TR, Anzola MC, et al. Induction of lupus inclusions by sera from patients with systemic lupus erythematosus. Arthritis Rheum 1986;29:501–7. 10.1002/art.1780290407 3486663

[213] Petri M, Singh S, Tesfasyone H, et al. Longitudinal expression of type I interferon responsive genes in systemic lupus erythematosus. Lupus 2009;18:980–9. 10.1177/0961203309105529 19762399 PMC4752166

[214] Cantaert T, van Baarsen LG, Wijbrandts CA, et al. Type I interferons have no major influence on humoral autoimmunity in rheumatoid arthritis. Rheumatology (Oxford) 2010;49:156–66. 10.1093/rheumatology/kep345 19933783

[215] Thurlings RM, Boumans M, Tekstra J, et al. Relationship between the type I interferon signature and the response to rituximab in rheumatoid arthritis patients. Arthritis Rheum 2010;62:3607–14. 10.1002/art.27702 20722020

[216] James JA, Guthridge JM, Chen H, et al. Unique Sjögren’s syndrome patient subsets defined by molecular features. Rheumatology (Oxford) 2020;59:860–8. 10.1093/rheumatology/kez335 31497844 PMC7188221

[217] Olsson P, Bodewes ILA, Nilsson AM, et al. Associations of cigarette smoking with disease phenotype and type I interferon expression in primary Sjögren’s syndrome. Rheumatol Int 2019;39:1575–84. 10.1007/s00296-019-04335-3 31139950

[218] Król P, Kryštůfková O, Polanská M, et al. Serum levels of interferon α do not correlate with disease activity in patients with dermatomyositis/polymyositis. Ann Rheum Dis 2011;70:879–80. 10.1136/ard.2010.141051 21068097

[219] Huard C, Gullà SV, Bennett DV, et al. Correlation of cutaneous disease activity with type 1 interferon gene signature and interferon β in dermatomyositis. Br J Dermatol 2017;176:1224–30. 10.1111/bjd.15006 27564228

[220] Mathian A, Mouries-Martin S, Dorgham K, et al. Monitoring disease activity in systemic lupus erythematosus with single-molecule array digital enzyme-linked immunosorbent assay quantification of serum interferon-α. Arthritis Rheumatol 2019;71:756–65. 10.1002/art.40792 30507062

[221] Steiman AJ, Gladman DD, Ibañez D, et al. Lack of interferon and proinflammatory cyto/chemokines in serologically active clinically quiescent systemic lupus erythematosus. J Rheumatol 2015;42:2318–26. 10.3899/jrheum.150040 26568589

[222] van Baarsen LG, Wijbrandts CA, Rustenburg F, et al. Regulation of IFN response gene activity during infliximab treatment in rheumatoid arthritis is associated with clinical response to treatment. Arthritis Res Ther 2010;12:R11. 10.1186/ar2912 20096109 PMC2875639

[223] Lübbers J, Brink M, van de Stadt LA, et al. The type I IFN signature as a biomarker of preclinical rheumatoid arthritis. Ann Rheum Dis 2013;72:776–80. 10.1136/annrheumdis-2012-202753 23434571

[224] Furie R, Khamashta M, Merrill JT, et al. Anifrolumab, an anti-interferon-α receptor monoclonal antibody, in moderate-to-severe systemic lupus erythematosus. Arthritis Rheumatol 2017;69:376–86. 10.1002/art.39962 28130918 PMC5299497

[225] Kalunian KC, Merrill JT, Maciuca R, et al. A phase II study of the efficacy and safety of rontalizumab (rhumab interferon-α) in patients with systemic lupus erythematosus (rose). Ann Rheum Dis 2016;75:196–202. 10.1136/annrheumdis-2014-206090 26038091

[226] Merrill JT, Furie R, Werth VP, et al. Anifrolumab effects on rash and arthritis: impact of the type I interferon gene signature in the phase IIb MUSE study in patients with systemic lupus erythematosus. Lupus Sci Med 2018;5:e000284. 10.1136/lupus-2018-000284 30588322 PMC6280909

[227] Petri M, Wallace DJ, Spindler A, et al. Sifalimumab, a human anti-interferon-α monoclonal antibody, in systemic lupus erythematosus: a phase I randomized, controlled, dose-escalation study. Arthritis Rheum 2013;65:1011–21. 10.1002/art.37824 23400715 PMC3654174

[228] Vosslamber S, Raterman HG, van der Pouw Kraan TCTM, et al. Pharmacological induction of interferon type I activity following treatment with rituximab determines clinical response in rheumatoid arthritis. Ann Rheum Dis 2011;70:1153–9. 10.1136/ard.2010.147199 21444302

[229] Sanayama Y, Ikeda K, Saito Y, et al. Prediction of therapeutic responses to tocilizumab in patients with rheumatoid arthritis: biomarkers identified by analysis of gene expression in peripheral blood mononuclear cells using genome-wide DNA microarray. Arthritis Rheumatol 2014;66:1421–31. 10.1002/art.38400 24591094

[230] Raterman HG, Vosslamber S, de Ridder S, et al. The interferon type I signature towards prediction of non-response to rituximab in rheumatoid arthritis patients. Arthritis Res Ther 2012;14:R95. 10.1186/ar3819 22540992 PMC3446469

[231] de Jong TD, Vosslamber S, Blits M, et al. Effect of prednisone on type I interferon signature in rheumatoid arthritis: consequences for response prediction to rituximab. Arthritis Res Ther 2015;17:78. 10.1186/s13075-015-0564-y 25889713 PMC4416246

[232] Wampler Muskardin T, Vashisht P, Dorschner JM, et al. Increased pretreatment serum IFN-β/α ratio predicts non-response to tumour necrosis factor α inhibition in rheumatoid arthritis. Ann Rheum Dis 2016;75:1757–62. 10.1136/annrheumdis-2015-208001 26546586 PMC4860184

[233] Reed AM, Peterson E, Bilgic H, et al. Changes in novel biomarkers of disease activity in juvenile and adult dermatomyositis are sensitive biomarkers of disease course. Arthritis Rheum 2012;64:4078–86. 10.1002/art.34659 22886447 PMC3510329

[234] Reed AM, Crowson CS, Hein M, et al. Biologic predictors of clinical improvement in rituximab-treated refractory myositis. BMC Musculoskelet Disord 2015;16:257. 10.1186/s12891-015-0710-3 26382217 PMC4574570

[235] Quartuccio L, Mavragani CP, Nezos A, et al. Type I interferon signature may influence the effect of belimumab on immunoglobulin levels, including rheumatoid factor in Sjögren’s syndrome. Clin Exp Rheumatol 2017;35:719–20. 28281461

[236] Aranow C, Kamen DL, Dall’Era M, et al. Randomized, double-blind, placebo-controlled trial of the effect of vitamin D3 on the interferon signature in patients with systemic lupus erythematosus. Arthritis Rheumatol 2015;67:1848–57. 10.1002/art.39108 25777546 PMC4732716

[237] Hasni S, Gupta S, Davis M, et al. Safety and tolerability of omalizumab: a randomized clinical trial of humanized anti-IgE monoclonal antibody in systemic lupus erythematosus. Arthritis Rheumatol 2019;71:1135–40. 10.1002/art.40828 30597768 PMC6594871

[238] McBride JM, Jiang J, Abbas AR, et al. Safety and pharmacodynamics of rontalizumab in patients with systemic lupus erythematosus: results of a phase I, placebo-controlled, double-blind, dose-escalation study. Arthritis Rheum 2012;64:3666–76. 10.1002/art.34632 22833362

[239] Merrill JT, Wallace DJ, Petri M, et al. Safety profile and clinical activity of sifalimumab, a fully human anti-interferon α monoclonal antibody, in systemic lupus erythematosus: a phase I, multicentre, double-blind randomised study. Ann Rheum Dis 2011;70:1905–13. 10.1136/ard.2010.144485 21798883

[240] Alexander T, Sarfert R, Klotsche J, et al. The proteasome inhibitior bortezomib depletes plasma cells and ameliorates clinical manifestations of refractory systemic lupus erythematosus. Ann Rheum Dis 2015;74:1474–8. 10.1136/annrheumdis-2014-206016 25710470 PMC4484251

[241] Petri M, Fu W, Ranger A, et al. Association between changes in gene signatures expression and disease activity among patients with systemic lupus erythematosus. BMC Med Genomics 2019;12:4. 10.1186/s12920-018-0468-1 30626389 PMC6327466

[242] von Wussow P, Jakschies D, Hartung K, et al. Presence of interferon and anti-interferon in patients with systemic lupus erythematosus. Rheumatol Int 1988;8:225–30. 10.1007/BF00269199 3266358

[243] Quartier P, Allantaz F, Cimaz R, et al. A multicentre, randomised, double-blind, placebo-controlled trial with the interleukin-1 receptor antagonist anakinra in patients with systemic-onset juvenile idiopathic arthritis (ANAJIS trial). Ann Rheum Dis 2011;70:747–54. 10.1136/ard.2010.134254 21173013 PMC3070271

[244] Bienkowska J, Allaire N, Thai A, et al. Lymphotoxin-LIGHT pathway regulates the interferon signature in rheumatoid arthritis. PLoS ONE 2014;9:e112545. 10.1371/journal.pone.0112545 25405351 PMC4236099

[245] López De Padilla CM, Crowson CS, Hein MS, et al. Interferon-regulated chemokine score associated with improvement in disease activity in refractory myositis patients treated with rituximab. Clin Exp Rheumatol 2015;33:655–63. 26446265

[246] Higgs BW, Zhu W, Morehouse C, et al. A phase 1B clinical trial evaluating sifalimumab, an anti-IFN-α monoclonal antibody, shows target neutralisation of a type I IFN signature in blood of dermatomyositis and polymyositis patients. Ann Rheum Dis 2014;73:256–62. 10.1136/annrheumdis-2012-202794 23434567 PMC3888620

[247] Dastmalchi M, Grundtman C, Alexanderson H, et al. A high incidence of disease flares in an open pilot study of infliximab in patients with refractory inflammatory myopathies. Ann Rheum Dis 2008;67:1670–7. 10.1136/ard.2007.077974 18272672

[248] McNab F, Mayer-Barber K, Sher A, et al. Type I interferons in infectious disease. Nat Rev Immunol 2015;15:87–103. 10.1038/nri3787 25614319 PMC7162685

[249] Fuertes MB, Woo S-R, Burnett B, et al. Type I interferon response and innate immune sensing of cancer. Trends Immunol 2013;34:67–73. 10.1016/j.it.2012.10.004 23122052 PMC3565059

[250] Chen H-J, Tas SW, de Winther MPJ. Type-I interferons in atherosclerosis. J Exp Med 2020;217:e20190459. 10.1084/jem.20190459 31821440 PMC7037237

[251] Wahadat MJ, Schonenberg-Meinema D, van Helden-Meeuwsen CG, et al. Gene signature fingerprints stratify SLE patients in groups with similar biological disease profiles: a multicentre longitudinal study. Rheumatology (Oxford) 2022;61:4344–54. 10.1093/rheumatology/keac083 35143620 PMC9629374

[252] Northcott M, Gearing LJ, Bonin J, et al. Immunosuppressant exposure confounds gene expression analysis in systemic lupus erythematosus. Front Immunol 2022;13:964263. 10.3389/fimmu.2022.964263 36059457 PMC9430375

[253] Omdal R, Mellgren SI, Koldingsnes W, et al. Fatigue in patients with systemic lupus erythematosus: lack of associations to serum cytokines, antiphospholipid antibodies, or other disease characteristics. J Rheumatol 2002;29:482–6. 11908560

[254] Davies K, Mirza K, Tarn J, et al. Fatigue in primary Sjögren’s syndrome (PSS) is associated with lower levels of proinflammatory cytokines: a validation study. Rheumatol Int 2019;39:1867–73. 10.1007/s00296-019-04354-0 31250166 PMC6791914

[255] Prak RF, Arends S, Verstappen GM, et al. Fatigue in primary Sjögren’s syndrome is associated with an objective decline in physical performance, pain and depression. Clin Exp Rheumatol 2022;40:2318–28. 10.55563/clinexprheumatol/70s6cs 36226629

[256] Howard Tripp N, Tarn J, Natasari A, et al. Fatigue in primary Sjögren’s syndrome is associated with lower levels of proinflammatory cytokines. RMD Open 2016;2:e000282. 10.1136/rmdopen-2016-000282 27493792 PMC4964201

[257] Posada J, Valadkhan S, Burge D, et al. Improvement of severe fatigue following nuclease therapy in patients with primary Sjögren’s syndrome: a randomized clinical trial. Arthritis Rheumatol 2021;73:143–50. 10.1002/art.41489 32798283 PMC7839752

[258] Collins A, Lendrem D, Wason J, et al. Revisiting the JOQUER trial: stratification of primary Sjögren’s syndrome and the clinical and interferon response to hydroxychloroquine. Rheumatol Int 2021;41:1593–600. 10.1007/s00296-021-04927-y 34165604 PMC8316226

[259] Bodewes ILA, Al-Ali S, van Helden-Meeuwsen CG, et al. Systemic interferon type I and type II signatures in primary Sjögren’s syndrome reveal differences in biological disease activity. Rheumatology (Oxford) 2018;57:921–30. 10.1093/rheumatology/kex490 29474655

[260] Vital EM, Merrill JT, Morand EF, et al. Anifrolumab efficacy and safety by type I interferon gene signature and clinical subgroups in patients with SLE: post hoc analysis of pooled data from two phase III trials. Ann Rheum Dis 2022;81:951–61. 10.1136/annrheumdis-2021-221425 35338035 PMC9213795

[261] Lipsky PE, Vollenhoven R van, Dörner T, et al. Biological impact of iberdomide in patients with active systemic lupus erythematosus. Ann Rheum Dis 2022;81:1136–42. 10.1136/annrheumdis-2022-222212 35477518 PMC9279852

