# Supplementary material for: Association between type I interferon pathway activation and clinical outcomes in rheumatic and musculoskeletal diseases: a systematic literature review informing EULAR points to consider
Source: RMD Open. 2023 Mar 7;9(1):e002864. doi: 10.1136/rmdopen-2022-002864 (PMC10008483; doi:10.1136/rmdopen-2022-002864)
Supplement: Supplementary data [file rmdopen-2022-002864supp001.pdf]

SUPPLEMENTARY MATERIAL

**Table of content**
Supplementary Text
Supplementary Text 1: Research questions (PICO framework)
Supplementary Text 2: Search strategy for Ovid MEDLINE
Supplementary Text 3: Search strategy for Embase
Supplementary Text 4: Search strategy for Web of Science
Supplementary Text 5: Inclusion and exclusion criteria (first step)
Supplementary Text 6: Eligibility criteria for clinical research questions (second step)
Supplementary Text 7: Risk of bias instruments
Supplementary Text 8: Summary and characteristics of assays measuring IFN pathway activation
Supplementary Tables
Supplementary Table 1: Summary of the studies reporting assays to evaluate the potential use of IFN assays in the diagnosis of SLE
Supplementary Table 2: Summary of the studies reporting assays to evaluate the potential use of IFN assays in the diagnosis of RA
Supplementary Table 3: Summary of the studies reporting assays to evaluate the potential use of IFN assays in the diagnosis of pSS
Supplementary Table 4: Summary of the studies reporting assays to evaluate the potential use of IFN assays in the diagnosis of SSc
Supplementary Table 5: Summary of the studies reporting assays to evaluate the potential use of IFN assays in the diagnosis of PM/DM
Supplementary Table 6: Summary of the studies reporting assays analyzing associations between IFN assays and disease activity in SLE
Supplementary Table 7: Summary of the studies reporting assays analyzing associations between IFN assays and disease activity in RA
Supplementary Table 8: Summary of the studies reporting assays analyzing associations between IFN assays and disease activity in pSS
Supplementary Table 9: Summary of the studies reporting assays analyzing associations between IFN assays and disease activity in SSc
Supplementary Table 10: Summary of the studies reporting assays analyzing associations between IFN assays and disease activity in PM/DM
Supplementary Table 11: Summary of the studies reporting assays analyzing associations between IFN assays and prognosis (natural history) in SLE
Supplementary Table 12: Summary of the studies reporting assays analyzing associations between IFN assays and prognosis (natural history) in RA
Supplementary Table 13: Summary of the studies reporting assays analyzing associations between IFN assays and prognosis (natural history) in SSc
Supplementary Table 14: Summary of the studies reporting assays analyzing associations between IFN assays and prognosis (response to treatments) in SLE
Supplementary Table 15: Summary of the studies reporting assays analyzing associations between IFN assays and prognosis (response to treatments) in RA

Supplementary Table 16: Summary of the studies reporting assays analyzing associations between IFN assays and prognosis (response to treatments) in PM/DM
Supplementary Table 17: Summary of the studies reporting assays analyzing associations between IFN assays and prognosis (response to treatments) in pSS
Supplementary Table 18: Summary of the studies reporting assays analyzing responsiveness to change of IFN assays in SLE
Supplementary Table 19: Summary of the studies reporting assays analyzing responsiveness to change of IFN assays in RA
Supplementary Table 20: Summary of the studies reporting assays analyzing responsiveness to change of IFN assays in SSc
Supplementary Table 21: Summary of the studies reporting assays analyzing responsiveness to change of IFN assays in pSS
Supplementary Table 22: Summary of the studies reporting assays analyzing responsiveness to change of IFN assays in PM/DM
References

Supplementary Text 1: Research questions (PICO framework)

Research questions	***Population***	***Intervention (Exposure)***	***Comparison***	***Outcome***
**What is the evidence that interferon measurement is useful in the diagnosis of RMDs?**				
	People presenting with any RMD<br><br>Control population	IFN pathway assay measurement	RMD vs control population	Association between measurement of IFN pathway activation and RMD diagnosis
**What is the evidence that interferon measurement reflects disease activity in RMDs?**				
	People presenting with any RMD	IFN pathway assay measurement	Disease activity measurement	Association between measurement of IFN pathway activation and disease activity (difference between groups, association, correlation)
**What is the evidence that interferon measurement is useful for the prognosis (natural history) of clinical status in RMDs?**				
	People presenting with any RMD, 'at-risk' of RMDs or in preclinical	IFN pathway assay measurement	Disease exacerbation / flare vs no disease exacerbation / no flare	Association between measurement of IFN pathway activation and disease exacerbation,

	stages		Progression to clinical RMD (fulfillment of classification criteria)<br><br>Severity of the clinical course (occurrence of comorbidity or organ involvement or damage)	progression to clinical RMD or severity
**What is the evidence that interferon measurement is useful for the prognosis (response to treatment) in RMDs?**				
	People presenting with any RMD starting a new therapy	IFN pathway assay measurement	Clinical response to treatment	Association between measurement of IFN pathway activation and clinical response
**What is the evidence that interferon measurement is responsive (i.e. changes with changing disease status or treatment)?**				
	People presenting with any RMD	IFN pathway assay measurement	Change in serial IFN measurements	Responsiveness (change) of serial measurements of IFN pathway activation

Supplementary Text 2: Search strategy for Ovid MEDLINE

1 interferon/ (21958)

2 (interferon* adj2 (biomarker* or sign*)).ti. (992)

3 (interferon* adj2 (biomarker* or sign*)).ab. /freq=2 (246)

4 exp interferon type i/ (48691)

5 "type 1 IFN".ti. (41)

6 "type 1 IFN".ab. /freq=2 (90)

7 (type 1 adj3 interferon*).ti. (205)

8 (type 1 adj3 interferon*).ab. /freq=2 (98)

9 (interferon* adj1 (alpha or alfa)).ti. (12678)

10 (interferon* adj1 (alpha or alfa)).ab. /freq=2 (5014)

11 (interferon* adj1 beta).ti. (4226)

12 (interferon* adj1 beta).ab. /freq=2 (1557)

13 or/1-12 [inteferons] (71976)

14 Lupus Erythematosus, Systemic/ (53310)

15 (systemic adj2 lupus).ti. (28721)

16 (systemic adj2 lupus).ab. /freq=2 (4269)

17 exp Arthritis, Rheumatoid/ (110425)

18 ((reumat* or rheumat* or psoriatic or juvenile or inflammatory or idiopathic) adj3 (arthrit* or artrit*)).ti. (71763)

19 ((reumat* or rheumat* or psoriatic or juvenile or inflammatory or idiopathic) adj3 (arthrit* or artrit*)).ab. /freq=2 (21982)

20 Arthralgia/ (7938)

21 arthralgia.ti. (631)

22 arthralgia.ab. /freq=2 (888)

23 Connective Tissue Diseases/ (6410)

24 connective tissue disease*.ti. (3395)

25 connective tissue disease*.ab. /freq=2 (1408)

26 exp Scleroderma, Systemic/ (20043)

27 (scleroderma or systemic sclerosis).ti. (17190)

28 (scleroderma or systemic sclerosis).ab. /freq=2 (6774)

29 Sjogren's Syndrome/ (12463)

30 ((sjogren* or sjoegren or sicca) adj2 syndrome).ti. (9830)

31 ((sjogren* or sjoegren or sicca) adj2 syndrome).ab. /freq=2 (3362)

32 (spondyloarthropath* or spondylarthropath*).ti. (1514)

33 (spondyloarthropath* or spondylarthropath*).ab. /freq=2 (748)

34 Spondylitis, Ankylosing/ (14342)

35 ankylosing spondylitis.ti. (8361)

36 ankylosing spondylitis.ab. /freq=2 (2758)

37 Vasculitis/ (12667)

38 vasculitis.ti. (11783)

39 vasculitis.ab. /freq=2 (9137)

40 Antiphospholipid Syndrome/ (7889)

41 (antiphospholipid syndrome or APS or APLS).ti. (5112)

42 (antiphospholipid syndrome or APS or APLS).ab. /freq=2 (9483)

43 Still's Disease, Adult-Onset/ (1280)

44 Still's Disease.ti. (1536)

45 Still's Disease.ab. /freq=2 (311)

46 exp Myositis/ (19181)

47 myositis.ti. (4738)

48 myostitis.ab. /freq=2 (0)

49 (Behcet* adj (disease or syndrome)).ti. (7844)

50 (Behcet* adj (disease or syndrome)).ab. /freq=2 (2829)

51 ((IgG4* or Immunoglobulin G4*) adj2 (syndrome or disease)).ti. (1246)

52 ((IgG4* or Immunoglobulin G4*) adj2 (syndrome or disease)).ab. /freq=2 (1188)

53 or/14-52 [RMD] (291413)

54 13 and 53 (1855)

55 exp animals/ not humans.sh. (4641021)

56 54 not 55 (1754)

57 remove duplicates from 56 (1744)

58 limit 57 to english language (1563)

Supplementary Text 3: Search strategy for Embase

1 interferon/ (78376)

2 (interferon* adj2 (biomarker* or sign*)).ti. (1447)

3 (interferon* adj2 (biomarker* or sign*)).ab. /freq=2 (518)

4 alpha interferon/ (54088)

5 alpha interferon A/ (289)

6 beta interferon/ (24399)

7 (interferon* adj1 (alpha or alfa)).ti. (15287)

8 (interferon* adj1 (alpha or alfa)).ab. /freq=2 (6077)

9 (interferon* adj1 beta).ti. (5808)

10 (interferon* adj1 beta).ab. /freq=2 (2295)

11 (type 1 adj3 interferon*).ti. (350)

12 "type 1 INF*".ab. /freq=2 (86)

13 or/1-12 [type 1 interferon] (151212)

14 systemic lupus erythematosus/ (96577)

15 (systemic adj2 lupus).ti. (40830)

16 (systemic adj2 lupus).ab. /freq=2 (6638)

17 exp Arthritis, Rheumatoid/ (219451)

18 ((reumat* or rheumat* or psoriatic or juvenile or inflammatory or idiopathic) adj3 (arthrit* or artrit*)).ti. (114993)

19 ((reumat* or rheumat* or psoriatic or juvenile or inflammatory or idiopathic) adj3 (arthrit* or artrit*)).ab. /freq=2 (41573)

20 Arthralgia/ (60181)

21 arthralgia.ti. (911)

22 arthralgia.ab. /freq=2 (1866)

23 Connective Tissue Diseases/ (11630)

24 connective tissue disease*.ti. (4727)

25 connective tissue disease*.ab. /freq=2 (2429)

26 Scleroderma, Systemic/ (17314)

27 (scleroderma or systemic sclerosis).ti. (26554)

28 (scleroderma or systemic sclerosis).ab. /freq=2 (12470)

29 Sjogren's Syndrome/ (12089)

30 ((sjogren* or sjoegren or sicca) adj2 syndrome).ti. (13295)

31 (spondyloarthropath* or spondylarthropath*).ab. /freq=2 (1029)

32 Spondylitis, Ankylosing/ (15590)

33 ankylosing spondylitis.ti. (13053)

34 ankylosing spondylitis.ab. /freq=2 (5036)

35 Vasculitis/ (40212)

36 vasculitis.ti. (16629)

37 vasculitis.ab. /freq=2 (15699)

38 Antiphospholipid Syndrome/ (16255)

39 (antiphospholipid syndrome or APS or APLS).ti. (7161)

40 (antiphospholipid syndrome or APS or APLS).ab. /freq=2 (14445)

41 Still's Disease, Adult-Onset/ (1317)

42 still's disease.ti. (2021)

43 still's disease.ab. /freq=2 (519)

44 Myositis/ (16388)

45 myositis.ti. (77)

46 myositis.ab. /freq=2 (37)

47 Behcet Syndrome/ (8519)

48 (Behcet* adj2 (syndrome or disease)).ti. (11087)

49 (Behcet* adj2 (syndrome or disease)).ab. /freq=2 (4765)

50 ((IgG4* or "Immunoglobulin G") adj2 (sydrome or disease)).ti. (1569)

51 ((IgG4* or "Immunoglobulin G") adj2 (sydrome or disease)).ab. /freq=2 (1713)

52 or/14-51 [RMD] (523688)

53 13 and 52 [interferon type 1 and RMD] (9125)

54 (exp animal/ or nonhuman/) not exp human/ (7037850)

55 53 not 54 (8634)

Supplementary Text 4: Search strategy for Web of Science

Indexes=SCI-EXPANDED, CPCI-S, ESCI Timespan=1900-2019

1 (TS=((interferon* Near/1 (alpha or alfa)))) AND LANGUAGE: (English)

2 (TS=((interferon* Near/1 beta))) AND LANGUAGE: (English)

3 (TS=((type 1 Near/3 interferon*))) AND LANGUAGE: (English)

4 TS=("type 1 INF*") or TS=(interferon* Near/2 biomarker*) or TS=(interferon* Near/2 sign*)

5 #4 OR #3 OR #2 OR #1

6 TS=((systemic Near/2 lupus))

7 TS=(((reumat* or rheumat* or psoriatic or juvenile or inflammatory or idiopathic) Near/3 (arthrit* or artrit*)))

8 TS=(Arthralgia)

9 TS=("connective tissue disease*")

10 TS=((scleroderma or "systemic sclerosis"))

11 TS=(((sjogren* or sjoegren or Sjogren's or sicca) Near/2 syndrome))

12 TS=((spondyloarthropath* or spondylarthropath*))

13 TS=("ankylosing spondylitis")

14 TS=(vasculitis)

15 TS=(("antiphospholipid syndrome" or APS or APLS))

16 TS=("still's disease")

17 TS=(Myositis)

18 TS=((Behcet* Near/2 (syndrome or disease)))

19 TS=(((IgG4* or "Immunoglobulin G") Near/2 (sydrome or disease)))

20 #19 OR #18 OR #17 OR #16 OR #15 OR #14 OR #13 OR #12 OR #11 OR #10 OR #9 OR #8 OR #7 OR #6

21 (#20 AND #5) AND LANGUAGE: (English)

22 (TS=("type 1 INF*") or TS=(interferon* Near/2 biomarker*) or TS=(interferon* Near/2 sign*)) AND LANGUAGE: (English)

24 (#23 AND #20) AND LANGUAGE: (English)

23 #22 OR #3 OR #2 OR #1

Supplementary Text 5: Inclusion and exclusion criteria (first step)

Inclusion criteria
• Subjects: human patients with RMDs<br>• RMDs: systemic lupus erythematosus (SLE), rheumatoid arthritis (RA), arthritis, juvenile idiopathic arthritis (JIA), arthralgia, Sjögren's syndrome (pSS), Sicca syndrome, dermatomyositis (DM), polymyositis (PM), myositis, connective tissue disease (CTD), scleroderma, systemic sclerosis (SSc), psoriatic arthritis (PsA), ankylosing spondylitis (AS), spondyloarthropathies (SpA), vasculitis, NCA-associated vasculitis (AAV), giant cell arteritis (GCA), antiphospholipid syndrome (APS), Still disease, adult-onset Still disease (AOSD), Behçet disease (BD), IgG4-related disease (IGRD)<br>• Language: English only<br>• Study design: longitudinal studies, cross-sectional studies, randomized controlled trials, case-control studies, cohort studies, non-controlled trials, intervention studies<br>• Samples: blood, serum, plasma studies
Exclusion criteria
• Subjects: animal studies, pre-clinical studies, genetic studies<br>• Study design: case studies, letters, non-original articles (reviews, editorials, opinion pieces, etc)<br>• Samples: peripheral tissues or fluids other than blood, serum or plasma<br>• Papers that do not specify the type of interferon that the assay measures

Supplementary Text 6: Eligibility criteria for clinical research questions

RQ1: What is the evidence that interferon measurement is useful in the diagnosis of RMDs?

- Clinical outcome: RMD diagnosis (validated classification criteria or diagnosis confirmed by a physician)
- Control population
- Study type: cross-sectional studies, diagnostic accuracy studies, case-control studies, cohort studies

RQ2: What is the evidence that interferon measurement reflects disease activity in RMDs?

- Clinical outcome: any disease activity measured by a validated instrument, including CRP or ESR levels, expressed as continuous or dichotomous variable
- Study type: longitudinal studies, randomized control trials, non-controlled trials, cross-sectional studies, case-control studies, cohort studies

RQ3: What is the evidence that interferon measurement is useful for the prognosis (natural history) of clinical status in RMDs?

- Clinical outcomes:
    - Disease exacerbation (pre-defined increase in disease activity)
    - Flare occurrence (by validated flare instruments)
    - Progression to clinical RMD (fulfillment of classification criteria)
    - Severity of the clinical course (occurrence of comorbidity or organ involvement or damage)
- Study type: longitudinal studies, randomized control trials, non-controlled trials, cohort studies

RQ4: What is the evidence that interferon measurement is useful for the prognosis (response to treatment) in RMDs?

- Clinical outcome: clinical response (validated response criteria or pre-defined change in disease active instrument) upon new treatment
- Study type: longitudinal studies, randomized control trials, non-controlled trials

RQ5: What is the evidence that interferon measurement is responsive (i.e. changes with changing disease status or treatment)?

- Clinical outcomes:
  - Change in measurements of IFN pathway activation before and after any treatment
  - Change in measurements of IFN pathway activation before and after disease exacerbation or flare
- Study type: longitudinal studies, randomized control trials, non-controlled trials, cohort studies

Supplementary Text 7: Risk of bias instruments

Research question	Instrument
**RQ1**	QUADAS-2 [1]
**RQ2**	JBI analytical cross sectional studies critical appraisal tool [2]
**RQ3**	QUIPS [3]
**RQ4**	Cochrane RoB2 [4]<br><br>QUIPS [3]
**RQ5**	Cochrane RoB2 [4]<br><br>JBI case series critical appraisal tool [5]

Supplementary Text 8: Summary and characteristics of assays measuring IFN pathway activation.

Category	Sub-category	Target	Sample source	Readout
**Immunoassays**	*Immunoassay*	Soluble protein(s) (IFNa/b proteins, IFN-stimulated proteins, chemokines, etc)	Serum, plasma, or whole blood lysates	Protein(s) concentration (individual or grouped as protein scores)
	DELFIA	Soluble protein(s) (IFNa/b proteins, IFN-stimulated proteins, chemokines, etc)	Serum or plasma	Protein(s) concentration (individual or grouped as protein scores)
	Simoa	Soluble protein(s) (IFNa/b proteins, IFN-stimulated proteins, chemokines, etc)	Serum or plasma	Protein(s) concentration (individual or grouped as protein scores)
	Acid-precipitation immunoassay	Soluble protein(s) (IFNa/b proteins, IFN-stimulated proteins, chemokines, etc)	Serum or plasma	Protein(s) concentration (individual or grouped as protein scores)
	Radioinmmunoassay	Soluble protein(s) (IFNa/b proteins, IFN-stimulated proteins, chemokines, etc)	Serum or plasma	Protein(s) concentration (individual or grouped as protein scores)
**Flow cytometry**	*Flow cytometry to detect IFN-induced proteins*	Membrane-bound IFN-stimulated proteins	Whole blood or isolated peripheral blood mononuclear cells (PBMCs)	Protein expression levels
**Microarrays**	*Microarray modules*	RNA from subsets of IFN-stimulated genes (usually pre-defined and sharing expression patterns)	RNA isolated from whole blood or PBMCs	Quantitative gene expression (fold enrichment/change) or categorical (number of genes up- and down-regulated, modular expression of gene

				signatures)
	Microarray scores and clustering	RNA from subsets of IFN-stimulated genes (usually pre-defined and sharing expression patterns)	RNA isolated from whole blood or PBMCs	Quantitative gene expression (gene scores)
**qPCR**	*qPCR*	RNA levels (IFN-a/b gene(s), IFN-stimulated gene(s), IFN-induced chemokine(s), etc)	RNA isolated from whole blood or PBMCs	Quantitative gene expression (fold enrichment/changes) either individual or composite scores (set of genes)
**RNA Seq**	*RNA Seq*	RNA from IFN-gene expression in whole transcriptome expression analysis	RNA isolated from whole blood or PBMCs	Quantitative gene expression or categorical (transcriptomic signature)
**Nanostring**	*Nanostring*	RNA from IFN-stimulated genes (pre-defined)	RNA isolated from whole blood or PBMCs	Quantitative gene expression (gene scores)
**DNA methylation**	*DNA methylation arrays*	Analysis of DNA methylation of IFN-stimulated genes in whole genome arrays	DNA from whole blood, isolated PBMCs or tissues	Number of genes with differential methylation, either individually or in gene sets
	Bisulfite sequencing	Analysis of DNA methylation in pre-defined IFN-stimulated genes sets	DNA from whole blood, isolated PBMCs or tissues	Number of genes with differential methylation, either individually or in pre-defined sets)
**Reporter cell assays**	*Reporter cell assays by qPCR*	IFN-stimulated gene(s) expression by qPCR in an experimental system upon stimulation with biological samples	Serum or plasma	Quantitative gene expression (fold

				enrichment/changes)
	Reporter cell assays (other)	IFN-stimulated gene(s) expression by luminometric or colorimetric in an experimental system upon stimulation with biological samples	Serum or plasma	Quantitative gene expression (fold enrichment/changes)
**Cytopathic effect assay**	*Cytopathic effect assay*	Functional (antiviral) effects of IFN in protecting cells from viral infections by analyzing cytopathic features	Serum or plasma	Antiviral activity measured in titres, arbitrary units, etc (quantitative)
**Plaque-reducing assay**	*Plaque-reducing assay*	Functional (antiviral) effects of IFN in protecting cells from viral infections by analyzing plaques of cultured cells	Serum or plasma	Measure of plaques of cultured cells (semi-quantitative)
**Immunohistochemistry (IHQ)**	*Immunohistochemistry (IHQ)*	IFN-stimulated proteins expression in tissues	Whole blood or other tissues	Protein levels (semi-quantitative)

Supplementary Table 1: Summary of the studies reporting assays to evaluate the potential use of IFN assays in the diagnosis of SLE.

Category	Sub-category	n assays	Results	Diagnostic statistics	Risk of bias	References
**Immunoassays**	*Immunoassays detecting IFNa protein*	21	17/21 studies found higher IFNa levels in SLE vs control populations<br>4 studies reported frequency of positive patients (from 40 to 72%) using different cut-offs	1 (Zecevic, 2018)<br>AUC 0.517 (0.382-0.653), p=0.807; S: 7.3%, Sp: 96.0%, PPV: 80.0%, NPV: 32.0%	High	[6–26]
	Immunoassays detecting IFNb protein	1	4 SLE patients exhibited high IFNb serum levels, whereas 2 exhibited similar levels to controls	0	High	[9]
	Immunoassays detecting IFN-induced proteins	6	***Targets:***<br>Mx1 (2), Galectin 3 – binding protein (1), galectin 9 (1), MIP1a (2)<br>***Main findings:***<br>Increased Mx1 levels in whole blood (lysed) in SLE (2)<br>Increased Galectin 3 serum levels in SLE (1)<br>Increased Galectin 9 – binding protein serum levels in SLE (1)<br>Increased MIP1a serum levels in SLE (1)	0	High	[27–32]
	DELFIA for IFNa	2	***Main findings:***<br>2/2 reported IFNa was detected more often in SLE (9/20 and 6/11) samples than controls (0 and 0 HC)	0	High	[33,34]

	DELFIA for IFN-induced proteins	1	***Targets***<br>sSIGLEC-1 (1)<br>***Main findings:***<br>sSIGLEC-1 serum levels increased in SLE vs HC	0	High	[28]
	Simoa for IFNa	2	***Main findings:***<br>2/2 reported IFNa serum levels higher in SLE than controls	0	High	[35,36]
	Other assays (Acid-precipitation immunoassay (1), RIA (2))	3		0	Unclear/high	[34,37,38]
**Flow cytometry**	*Flow cytometry to detect IFN-induced proteins*	6	***Targets:***<br>SIGLEC1 (3), CD64 (1), ITIM1 (1), Mx1 (1)<br>***Main findings:***<br>SIGLEC1 expression was higher in monocytes (2) and pDC (1) in SLE vs controls<br>Increased expression of CD64 (1) and Mx1 (1) in monocytes and ITIM1 in platelets (1) in SLE vs controls	0	High	[20,28,31,39–41]
**Microarrays**	*Microarray modules*	5	***Sources:***<br>WB (3), PBMC (1), sorted cell populations (CD4+ and CD8+ T cells, monocytes and neutrophils) (1)<br>***Main findings:***<br>5/5 reported IFN-related modules among the most upregulated genes in SLE vs HC<br>M1.2, M3.4, and M5.12 reported in 3/5<br>Modular IFN signature observed in SLE ranging from 83% to 100% of the patients	0	High	[42–46]

	Microarray scores and clustering	20	***Sources:***<br>WB (10), PBMC (4), sorted cell populations (CD4+, T-cells, B-cells, monocytes or platelets) (6)<br>***Main findings:***<br>15/20 reported IFN score and/or ISG expression significantly higher in SLE vs controls<br>IFN signature positive/high patients ranged from 50 to 81% (reported in 5/20)<br>1/20 reported IFN score follow a bimodal distribution (1)	0	High	[20,39,41,47–63]
**qPCR**	*qPCR on individual ISG*	4	***Sources:***<br>WB (2), PBMC (2)<br>***Targets:***<br>IFNA (1), Mx1 (2) or ISG15 (1)<br>***Main findings:***<br>Increased expression of IFNA (1), Mx1 (2) or ISG15 (1) in SLE vs controls	1 (Yuan, 2018): ISG15: AUC 0.826 (0.715–0.937, p=0.000015), S: 89.3%, Sp: 68.8%	High	[6,64–66]
	qPCR on several ISG	38	***Sources:***<br>WB (19), leukocytes (3), PBMC (11), sorted cell populations (monocytes or platelets) (5)<br>***Targets:***<br>ISG scores calculated from 2 (1), 3 (7), 4 (5), 5 (9), 6 (3), 8 (1), 9 (2), 10 (1) or 23 (1) genes;<br>4 studies computed 2 scores (GC-A/B, score A/B, type I/II)<br>***Main findings:***<br>38/38 reported IFN score and/or ISG expression significantly higher in SLE vs controls<br>Differences across ISGs reported in	1 (Feng, 2015): IFN score: AUC 0.805, $p=1.4\cdot10^{-5}$	Unclear/high	[18,20,29,31,47,56,62,67–96]

			4/38<br>IFN positive patients ranged from 57 to 64%			
	qPCR on chemokines	1	***Sources:***<br>Leukocytes (1)<br>***Targets:*** CCL2 and IP-10 (1)<br>***Main findings:***<br>Increased expression of CCL2 and IP-10 in SLE vs controls	0	High	[72]
**Nanostring**		1	***Sources:*** WB<br>***Targets:*** 5 ISG<br>***Main findings:***<br>IFN score higher in SLE vs controls	0	High	[97]
**DNA methylation**	*DNA methylation arrays*	9	***Sources:***<br>WB (2), PBMC (2), sorted cell populations (CD4+ T-cells, CD19+ B-cells, CD14+ monocytes, neutrophils and low density granulocytes) (5)<br>***Main findings:***<br>9/9 reported SLE-specific differentially methylated genes (mostly hypomethylated) related to ISG<br>6/9 reported ISG-related DMG among those with the largest size effect	1 (Imgenberg-Kreuz, 2019)<br>Hypomethylation (DMG): AUC 0.940	High	[98–106]
	Bisulfite sequencing	3	***Sources:***<br>WB (2), sorted cell populations (neutrophils) (1)<br>***Main findings:***<br>3/3 confirmed SLE-specific hypomethylated sites	1 (Zhao, 2016)<br>Hypomethylation IFI44L: Site 1: AUC 0.968 (0.954-0.981), S: 93.6%; Sp: 96.8%. Site 2: AUC 0.982 (0.972-0.992), S: 94.1%; Sp:	High	[100,102,107]

				98.2%		
**Reporter cell assays**	*Reporter cell assays by qPCR*	8	***Main findings:***<br>7/8 reported high IFN activity in SLE vs controls<br>1/8 reported no differences<br>1/8 reported important differences across genes	0	High	[86,108–114]
	Reporter cell assays (other)	4	***Main findings:***<br>4/4 reported higher IFN bioactivity in SLE vs controls<br>1 reported high IFN activity in 42% of SLE patients	0	High	[115–117]
**Cytopathic effect assay**		3	***Main findings:***<br>3/3 reported higher IFN activity in SLE vs controls	0	High	[118–120]
**Plaque-reducing assay**		4	***Main findings:***<br>4/4 reported higher IFN activity in SLE compared to controls	0	High	[121–124]
**IHQ**		1	***Target:***<br>MxA<br>***Main findings:***<br>Granulocytes and monocytes from SLE patients also stained positively for MxA protein	0	High	[32]

Supplementary Table 2: Summary of the studies reporting assays to evaluate the potential use of IFN assays in the diagnosis of RA.

Category	Sub-category	n assays	Results	Diagnostic statistics	Risk of bias	References
**Immunoassays**	*Immunoassays detecting IFNa protein*	2	2/2 studies found higher IFNa levels in RA vs control populations	0	High	[125,126]
	Immunoassays detecting IFNb protein	2	1/2 reported IFNb marginally detected in juvenile chronic arthritis patients<br>1/2 reported IFNb serum levels higher in juvenile chronic arthritis patients compared to controls	0	High	[125,127]
	DELFIA for IFNa	1	***Main findings:***<br>IFNa was detected more often in RA (5/19) samples than in controls (0)	0	High	[128]
	Other assays (RIA (2))	2	***Main findings:***<br>2/2 reported no differences in IFNa serum levels in RA patients vs controls	0	High	[129,130]
**Microarrays**	*Microarray scores and clustering*	7	***Sources:***<br>WB (5), PBMC (1), sorted cell populations (monocytes) (1)<br>***Targets:***<br>ISG scores ranging from 5 to 43 ISG<br>***Main findings:***<br>7/7 reported IFN score and/or ISG expression significantly higher in RA vs HC<br>IFN signature positive/high patients ranged from 22% to 45% (reported in 2/7)<br>IFN signature qualitatively differs between RA and SLE (reported in 2/7)	0	High	[52,57,61,131–134]
**qPCR**	*qPCR on individual ISG*	3	***Sources:***<br>WB (3)<br>***Targets:***<br>IFIT1, IFIT2, IFI44L, ISG15, MXA, MXB, EPSTRLI1, RSAD, HERC5, Ly6E, IFI6, IFI35 (1), IFI44, IFI44L, IFI6, and MX1 (2) or IFI35,	0	High	[84,125,135]

			IFIT3, IFI44, IFI44L, OAS1, SIGLEC1 (1)<br>***Main findings:***<br>1 reported MX1, MXB, IFIT1 and IFIT2, HERC5, ISG15, LY6E, RSAD2, IFI25 increased in established RA but not in early RA vs controls. No differences for IFI44, IFI6<br>1 reported IFI44L, IFI6, MX1 and IRF4 increased in RA. No differences in IFI44<br>1 reported IFI35 increased in RA. No differences in IFI44 (1)			
	qPCR on several ISG	6	***Sources:***<br>WB (5), PBMC (1)<br>***Targets:***<br>ISG scores calculated from 3 (1), 4 (1), 5 (2), 7 (1) or 23 (1) genes; 1 study computed 2 scores (GC-A/B)<br>***Main findings:***<br>6/6 studies reported IFN score increased in RA (any subgroup) vs controls<br>1/6 reported IFN score higher in early RA but not in established<br>1/6 reported IFN score higher in established vs very early<br>IFN positive patients ranged from 13% to 40% (reported in 3/6)	0	Unclear/high	[70,77,109,136–138]
	qPCR on chemokines	1	***Sources:*** WB<br>***Targets:***<br>RANTES, MCP-1, CCL19, MIG, IP-10, CXCL11, and IL-8 (1)<br>***Main findings:***<br>Increased expression of IFN-induced chemokine score in RA vs controls	0	High	[77]
**RNA-seq**		1	***Sources:*** sorted cell populations (neutrophils)<br>***Main findings:***<br>Most differently regulated signaling pathway in RA neutrophils was IFN signaling	0	High	[139]

Rodríguez-Carrio J, *et al. RMD Open* 2023; 9:e002864. doi: 10.1136/rmdopen-2022-002864

**Reporter cell assays**	*Reporter cell assays by qPCR*	2	***Main findings:***<br>1/2 reported high IFN activity in 29.7% of RA patients vs 6.3% of controls	0	High	[109,140]
	Reporter cell assays (other)	1	***Main findings:***<br>1/1 detected decreased IFN-like substance in RA vs controls (below sensitivity in 39/49 of RA patients)	0	High	[130]
**Cytopathic effect assay**		1	***Main findings:***<br>Higher OAS activity in RA inactive vs active patients and vs controls	0	Unclear/high	[141]
**Plaque-reducing assay**		3	***Main findings:***<br>3/3 reported higher IFN activity in RA vs controls	0	High	[121,122,142]

Supplementary Table 3: Summary of the studies reporting assays to evaluate the potential use of IFN assays in the diagnosis of pSS.

Category	Sub-category	n assays	Results	Diagnostic statistics	Risk of bias	References
**Immunoassays**	*Immunoassays detecting IFNa protein*	4	4/4 reported higher IFNa serum levels in pSS vs controls	0	High	[97,143–145]
	Immunoassays detecting IFNb protein	1	IFNb detected in most samples, increased in pSS but not significant	0	High	[146]
	Immunoassays detecting IFN-induced proteins	4	***Targets:*** MCP-1, MIP-1α, MIP-1β, MIG and IP-10 (1), Mx1 (1), IFI16 (1), IP-10 (1) ***Main findings:*** 4/4 increased IFN-induced protein serum levels in pSS vs controls	0	Unclear/high	[143,147–149]
	DELFIA for IFNa	1	IFNa was detected more often in pSS (3/38) samples than HC (0 and 0 HC), but low levels in all three patients	0	High	[128]
	Other assays (RIA (1))	1	Circulating IFNa lower in pSS vs controls	0	High	[150]
**Flow cytometry**	*Flow cytometry to detect IFN-induced proteins*	2	***Targets:*** SIGLEC1 (1), Mx1 (1) ***Main findings:*** SIGLEC1 (1/1) and Mx1 (1/1) expression were higher in monocytes in pSS vs controls Mx1 expression was correlated to IFN score (1/1)	0	High	[148,149]
**Microarrays**	*Microarray scores and clustering*	3	***Sources:*** WB (1), PBMC (1), sorted cell populations (CD14+/low monocyte subsets) (1) ***Targets:*** ISG scores ranging from 9 to 128 ISG ***Main findings:*** 3/3 found IFN-related genes among the most differentially expressed genes	0	High	[146,151,152]

**qPCR**	*qPCR on individual ISG*	3	***Sources:***<br>WB (2), sorted cell populations (monocytes) (1)<br>***Targets:***<br>IFNA (2) or IFI27 (1)<br>***Main findings:***<br>Increased expression of IFNA (2/3) or IFI27 (1/3) in pSS vs controls<br>Increased expression of IFNA in a fraction of pSS patients (5/23 patients)		High	[144,146,152]
	qPCR on several ISG	7	***Sources:***<br>WB (4), PBMC (2), sorted cell populations (monocytes) (1)<br>***Targets:***<br>ISG scores calculated from 3 (1), 5 (5), 6 (1); 1 study computed 2 scores (type I/II)<br>***Main findings:***<br>7/7 reported IFN score significantly higher in pSS vs controls<br>IFN positive patients ranged from 51 to 70% (reported in 5/8)	0	High	[67,89,148,153–156]
**RNA-seq**		2	***Sources:***<br>WB (1), sorted cell populations (CD19+ B-cells)<br>***Main findings:***<br>2/2 reported several IFN-related genes among those differentially expressed between pSS and controls		High	[83,145]
**Nanostring**		3	***Sources:***<br>WB (2), sorted cell populations (CD19+ B-cells)<br>***Targets:*** several ISG<br>***Main findings:***<br>2/3 confirmed IFN-related genes increased in pSS vs controls<br>1/3 reported IFN score (5 genes) did not	0	High	[83,97,145]

			differ between pSS and controls			
**DNA methylation**	*DNA methylation arrays*	2	***Sources:***<br>WB (1), sorted cell populations (CD4+ T-cells)<br>***Main findings:***<br>1/2 differentially methylated sites related to IFN pathway<br>1/2 extensive hypomethylation in IFN-related genes (STAT1, IFI44L, IFITM1 and USP18)	1 (Imgenberg-Kreuz, 2018) AUC=0.910	High	[103,157]
**Reporter cell assays**	*Reporter cell assays by qPCR*	3	***Main findings:***<br>3/3 reported high IFN activity in pSS patients vs controls	0	High	[111,146,158]
	Reporter cell assays (other)	1	***Main findings:***<br>1/1 increased IFN activity in pSS vs controls	0	High	[117]
**Plaque-reducing assay**		2	***Main findings:***<br>2/2 reported higher IFN activity in pSS vs controls	0	Unclear/high	[121,122]

Supplementary Table 4: Summary of the studies reporting assays to evaluate the potential use of IFN assays in the diagnosis of SSc.

Category	Sub-category	n assays	Results	Diagnostic statistics	Risk of bias	References
**Immunoassays**	*Immunoassays detecting IFN-induced proteins*	4	***Targets:***<br>93 proteins (1), CXCL10, CXCL11, CCL10 and CCL8 (1), IP-10 and I-TAC (1), IP-10, MCP-1, MIP1a and RANTES (1)<br>***Main findings:***<br>1/4 reported increased IP10 and I-TAC in SSc vs controls<br>1/4 reported CXCL10 and CXCL11 increased in SSc vs controls<br>1/4 reported increased IP-10, MCP-1 and MIP1a in SSc vs controls<br>1/4 reported 37 proteins higher in SSc vs controls (including MCP1, MIP1b, RANTES, MIP3b, MIG, IP10, I-TAC, and MIF)<br>IFN chemokine score: higher in SSc vs controls (39.2% SSc positive in IFN inducible score)	0	High	[159–162]
	DELFIA for IFNa	1	***Main findings:***<br>33 and 23 out of 79 SSc sera induced IFNa when combined with necrotic or apoptotic material, higher compared to controls	0	High	[159]
**Flow cytometry**	*Flow cytometry to detect IFN-induced proteins*	1	***Targets:***<br>SIGLEC1 (1)<br>***Main findings:***<br>SIGLEC1 expression was higher in monocytes from SSc vs controls (almost absent)<br>SIGLEC1 was highly expressed in SSc patients with high IFN score	0	High	[163]

**Microarrays**	*Microarray modules*	1	***Sources:*** WB<br>***Main findings:***<br>IFN-related (M1.2 and M3.4) and one neutrophil module (M5.15) were the only statistically significant upregulated modules in SSc vs controls	0	High	[164]
	Microarray scores and clustering	9	***Sources:***<br>WB (6), PBMC (2), sorted cell populations (monocytes) (1)<br>***Targets:***<br>ISG scores ranging from 9 to 129 ISG<br>***Main findings:***<br>4/9 reported a variable number of IFN-related genes among those genes differentially expressed between SSc vs controls<br>4/9 reported higher IFN scores in SSc vs controls<br>1/9 reported some IFN-related genes among those genes differentially expressed between lcSSc vs controls<br>67% IFN positive patients in SSc (reported in 1/10)<br>IFN positive prevalence varied across disease phenotypes (RP/nc/lc/dc) from 33.3% to 100% (reported in 1/9)	1 (Brkic, 2015)<br>IFN score AUC: 0.823, S: 0.667, Sp: 0.881	Unclear/high	[41,47,52,57,160,163,165–167]
**qPCR**	*qPCR on individual ISG*	5	***Sources:***<br>WB (2), PBMC (3)<br>***Targets:***<br>MxA (1), SIGLEC (2), IRF7, G1P3 and S100A8 (1), or IFNA, IFNB, IRF7 OAS, MxA and 6-16 (1)<br>***Main findings:***<br>1/5 reported increased expression of MxA in 9/50 SSc vs controls	0	High	[163,166–169]

			2/5 reported increased expression of SIGLEC1 in SSc vs controls<br>1/5 reported increased expression of IRF7, G1P3 and S100A8 in SSc vs controls<br>1/5 reported increased expression of IFNA, IFNb and OAS in SSc vs controls			
	qPCR on several ISG	7	***Sources:***<br>WB (6), sorted cell populations (monocytes) (1)<br>***Targets:***<br>ISG scores calculated from 3 (1), 5 (4), 6 (1), or 11 (1) ISGs<br>***Main findings:***<br>6/7 reported IFN score significantly higher in SSc vs controls<br>1/7 reported IFN score elevated in 2/13 SSc patients	0	High	[47,83,89,165,170,171]
**Nanostring**		2	***Sources:***<br>WB (2)<br>***Main findings:***<br>1/2 IFN score in 2/13 SSc patients<br>1/2 IFN score (5 genes) did not exhibit differences between SSc and controls	0	High	[83,97]
**RNA-seq**		1	***Sources:*** sorted cell populations (monocytes) (1)<br>***Main findings:***<br>4/99 IFN-related lnRNA were upregulated in SSc vs controls		High	[161]
**DNA methylation**	*DNA methylation arrays*	1	***Sources:***<br>Sorted cell populations (CD4+ and CD8+ T-cells) (1)<br>***Main findings:***<br>Two IFN pathways were the most significantly enriched among	0	High	[172]

			hypomethylated regions			
**Reporter cell assays**	*Reporter cell assays by qPCR*	1	***Main findings:***<br>High IFN score in SSc (9/13) vs controls (0/13)	0	High	[173]
	Reporter cell assays (other)	2	***Main findings:***<br>2/2 IFN bioactivity did not differ between SSc vs controls	0	High	[117]
**Plaque-reducing assay**		3	***Main findings:***<br>2/3 IFN bioactivity increased in SSc vs controls<br>1/3 IFN bioactivity did not differ in SSc vs controls	0	Unclear/high	[121,122,169]

Supplementary Table 5: Summary of the studies reporting assays to evaluate the potential use of IFN assays in the diagnosis of PM/DM.

Category	Sub-category	n assays	Results	Diagnostic statistics	Risk of bias	References
**Immunoassays**	*Immunoassays detecting IFNa protein*	4	***Main findings:***<br>2/4 reported higher IFNa serum levels in PM/DM vs controls<br>1/4 reported higher IFNa serum levels in a subset of patients (anti-MDA5 positive)<br>1/4 IFNa serum levels did not differ between PM/DM vs controls	0	High	[97,174–176]
	Immunoassays detecting IFNb protein	3	***Main findings:***<br>2/3 IFNb serum levels elevated in PM/DM vs controls<br>1/3 IFNb serum levels did not differ between PM/DM and controls	0	High	[175,176]
	Simoa for IFNa	1	***Main findings:***<br>IFNa was increased in PM/DM vs controls	0	High	[36]
**Microarrays**	*Microarray scores and clustering*	6	***Sources:***<br>WB (5), PBMCs (1)<br>***Targets:***<br>ISG scores ranging from 5 to 43 ISGs<br>***Main findings:***<br>6/6 reported IFN-related genes among the most differentially expressed genes / largest fold change<br>1/6 reported signature in 10/12 PM/DM patients	0	High	[48,52,57,175,177,178]
**qPCR**	*qPCR on individual ISG*	3	***Sources:***<br>WB (2), PBMCs (1)<br>***Targets:***<br>MXA (1), IRF7, ISG15 and MXA (1) or IFIT1 and MXA (1)<br>***Main findings:***	0	High	[174,178,179]

			1/3 reported increased MXA expression in PM/DM<br>1/3 reported increased expression of IRF7, ISG15, MXA in PM/DM<br>1/3 reported increased expression of IFIT1 and MXA in PM/DM vs controls			
	qPCR on several ISG	7	***Sources:***<br>WB (6), PBMC (1)<br>***Targets:***<br>ISG scores calculated from 3 (1), 6 (2), 8 (1), 13 (1), 23 (1); 1 study computed 2 scores (GC-A/B)<br>***Main findings:***<br>6/7 reported IFN score elevated in PM/DM vs controls<br>1/7 reported IFN positive patients in 2/8 PM/DM patients<br>1/7 IFN score in PM/DM is predominant GC-A (SLE-like)	1 (Feng, 2015): IFN score: AUC 0.805, $p=1.4\cdot10^{-5}$	High	[36,73,83,136,177,180]
**RNA-seq**		2	***Sources:***<br>WB (1), sorted cell populations (B-cells)<br>***Main findings:***<br>1/2 reported IFN-related genes among the most significantly expressed genes between PM/DM vs controls<br>1/2 validated microarrays results	0	High	[83,181]
**Nanostring**		2	***Sources:***<br>WB (2)<br>***Main findings:***<br>1/2 IFN score increased in PM/DM patients (100%) vs controls<br>1/2 IFN score in 2/8 PM/DM patients	0	High	[83,97]
**Reporter cell assays**	*Reporter cell assays by qPCR*	3	***Main findings:***<br>1/3 reported high IFN activity in PM/DM patients<br>1/3 reported no increased IFN activity in	0	Unclear/high	[182–184]

			PM/DM (most did not induce up-regulation of the ISG, 2 induced high IFNa activity, and 5 induced low activity (+)) vs controls<br>1/3 high IFN bioactivity was dependent on IFN score status in PM/DM patients			
	Reporter cell assays (other)	1	***Main findings:***<br>Increased IFN activity present in 54% (13/24) of DM, 67% (8/12) of PM and 5% (1/22) of myositis samples	0	Unclear/high	[175]

Supplementary Table 6: Summary of the studies reporting assays analyzing associations between IFN assays and disease activity in SLE.

Category	Sub-category	Reference	n	Disease activity measure	Results	Risk of bias
**Immunoassays**	*Immunoassays detecting IFNa protein*	Abdel Galil, 2018 [6]	126	SLEDAI-2K	Positive correlation (r=0.640, p<0.001)	High
		Baechler, 2003 [49]	48	Number of SLE disease criteria	Positive correlation (p<0.0002)	High
		Becker-Merok, 2013 [7]	87	SLEDAI	No association (p=0.070)	High
		Bengtsson, 2000 [185]	30	SLEDAI	Positive correlation (r=0.479, p=0.007)	High
		Fernández-Matilla, 2000 [19]	142	SELENA-SLEDAI<br>CRP<br>ESR	No association	Unclear
		Fragoso-Loyo, 2012 [8]	34	SLEDAI-2K	Positive correlation (r=0.330, p=0.050)	High
		Jonsen, 2003 [186]	57	SLEDAI	No association	High
		Kim, 1987 [38]	30	Clinical activity score	Positive correlation (r=0.600, p<0.010)	High
		Mathian, 2019 [187]	68	SELENA-SLEDAI	Positive association (high SELENA-SLEDAI score: AUC 0.830; Sensitivity: 68.7 (57.6–78.4); specificity: 85.1 (74.3–92.6); PPV: 85.1; NPV 68.7)	High
		Mathian, 2019b [35]	254	Remission (SELENA-SLEDAI)	Positive association (abnormal IFN levels associated with no remission (OR 11.4, p<0.0001))	High
		Oke, 2017 [11]	261	SLEDAI	No association	Unclear

		Postal, 2012 [12]	57	SLEDAI	Positive correlation (r=0.430, p=0.012)	High
		Rodero, 2017 [36]	72	SLEDAI<br>ESR<br>CRP	SLEDAI: positive association (higher SLEDAI in patients with high IFN (p<0.001)<br>ESR: positive association (higher ESR in patients with high IFN (p<0.010)<br>CRP: no association (ns)	Low
		Rose, 2013 [188]	79	mSLEDAI<br>BILAG-2004	mSLEDAI: positive correlation (r=0.4359, p<0.0001)<br>BILAG-2004: positive correlation (r=0.4476, p<0.0001)	Low
		Rose, 2017 [189]	26	BILAG-2004	Positive correlation (r=0.420, p=0.0022)	High
		Schneider, 2015 [190]	172	SLEDAI<br>SLICC	SLEDAI: positive correlation (r=0.219, p=0.004)<br>SLICC: no association (r=0.063, p=ns)	Low
		Shi, 1987 [37]	47	Disease activity stages	Positive association (81% samples of patients with high disease activity were positive compared to 10% samples positive during quiescent stages; p<0.005)	High
		Willis, 2012 [14]	35	SLAM-R	Positive correlation (r=0.314, p=0.054)	High
		Yin, 2014 [16]	79	SLEDAI<br>CRP	SLEDAI: no correlation (r=0.129, p=0.264)<br>CRP: no correlation (r=0.183, p=0.126)	Unclear
		Hashad, 2012 [21]	52	SLEDAI	Positive correlation (p=0.030)	High
		Ma, 2012 [23]	37	SLEDAI	No association (active SLE vs inactive SLE: p=0.690; active SLE vs HC: p=0.098; inactive SLE vs HC: p=0.077)	High
		Mandal, 2014 [24]	129	SLEDAI	Positive correlation (r=0.260, p=0.002)	High
		Robak, 2004 [26]	36	SLAM	No association	High
		Oke, 2019 [191]	497	SLEDAI<br>SDI<br>SLAM	SLEDAI: no association (high IFNa: OR 0.9 (0.5-1.9), p=ns)<br>SDI: no association (high IFNa: OR 0.8 (0.4-1.6), p=ns)<br>SLAM: no association (high IFNa: OR 0.8 (0.4-1.6), p=ns)	Unclear
	Immunoassays detecting IFNb protein	Munroe, 2017 [192]	13	SELENA-SLEDAI	Positive correlation (p<0.0001)	High

	Immunoassays detecting IFN-induced proteins	Oliveira, 2018 [28]	34	SLEDAI	Positive correlation (sSIGLEC: r=0.100, p=0.070)	High
		Zhuang, 2005 [18]	66	SLEDAI	No association (Mx1: ns)	High
		Bauer, 2006 [193]	30	SLEDAI<br>SLAM-R<br>ESR	SLEDAI: positive correlation (MCP-2: r=0.520, p<0.010; MCP-1: r=0.350, p<0.050; I-TAC: r=0.420, p<0.050; IP-10: r=0.370, p<0.050; MIP-3B: p<0.001; MIP-1A: r=0.590, p<0.010); no association (MIG: p=ns)<br>SLAM-R: positive correlation (MCP-2; r=0.600, p<0.010; MCP-1: r=0.390, p<0.050; MIG: r=0.480, p<0.010; I-TAC: r=0.670, p<0.0001; IP-10: r=0.500, p<0.010; MIP-3B: r=0.57, p<0.001; MIP-1A: r=0.680, p<0.001)<br>ESR: positive correlation (MCP-2: r=0.620, p<0.010; MCP-1: r=0.540, p<0.050; MIG: r=0.560, p<0.010; I-TAC r=0.700, p<0.0001; IP-10: r=0.560, p<0.001; MIP-3B: r=0.330, p<0.050; MIP-1A: r=0.600, p<0.001)	Unclear
		Bauer, 2009 [194]	267	SLEDAI	Positive association (chemokine score: SLEDAI was elevated in chemokine-high patients compared to chemokine-intermediate ($p=3.8\times10^{-5}$) and chemokine-low patients ($p=1.0\times10^{-7}$).	High
		Casey, 2018 [90]	304	SLEDAI-2K<br>CLASI	SLEDAI-2K: positive correlation (27 proteins were significantly correlated with SLEDAI-2K and significantly higher in high SLEDAI-2K vs low SLEDAI-2K)<br>CLASI: positive correlation (7 proteins were significantly correlated with CLASI and showed higher concentrations in patients with high CLASI)	Unclear
		Connelly, 2016 [195]	151	Adjusted mean SLEDAI	Positive association (high IFN chemokine score scores were significantly associated with high disease activity (OR [95% CI]: 2.56 [1.03, 6.37]; p=0.040)	High
		Connelly, 2018 [196]	109	SLEDAI-2K	Positive correlation (IFN chemokine score: r=0.730, (95% CI: 0.12, 1.43), p=0.020)	High
		Lee, 2016 [197]	30	SLEDAI	Positive association (IFN chemokine score high patients exhibited higher SLEDAI compared to IFN low group, p=0.0006)	High

		Munroe, 2017 [192]	13	SELENA-SLEDAI	Positive correlation (MCP1/CCL2: p=0.0203; MCP3/ccl7: p=0.0022; MIP1ab/CCL4: p=0.0388; CXCVL8/IL8: p=0.0034; MIG/CXCL: p=0.0159; IP10/CXCL10: p=0.0298)	High
		Nielsen, 2014 [27]	70 SLE-DK 68 SLE-SE 26 SLE-IFNa	SLEDAI SLICC	SLEDAI: positive association (Galectin 3–binding protein: SLE-DK: r=0.410 p=0.0004; SLE-IFNa: r=0.420 p=0.030); no association (Galectin 3–binding protein: SLE-SE: r=−0.0025 p=0.980) SLICC: no association (Galectin 3–binding protein: SLE-DK: r=−0.08 p=0.530; SLE-SE: r=0.036 p=0.770)	Low
		Rose, 2013 [188]	79	mSLEDAI-2K BILAG-2004	mSLEDAI-2K: positive correlation (IP-10: r=0.335, p=0.001; SIGLEC1: r=0.434, p<0.001) BILAG-2004: positive correlation (IP-10: r=0.385, p<0.001; SIGLEC1: r=0.540, p<0.001)	Low
		Rose, 2017 [189]	26	BILAG-2004	Positive correlation (IP-10: r=0.500, p=0.002)	High
		Van den Hoogen, 2018 [87]	50	SLEDAI	Positive correlation (Galectin-9: r=0.320, p=0.003)	High
		Wahadat, 2018 [31]	23	SELENA-SLEDAI	No association (MxA: ns)	High
**Flow cytometry**	*Flow cytometry to detect IFN-induced proteins*	Biesen, 2008 [41]	25	SLEDAI	***Target (source):*** Siglec1 (monocytes) ***Main findings:*** positive correlation: Siglec1–positive inflammatory monocytes and Siglec1–positive resident monocytes both correlated with SLEDAI (p=0.006 and p=0.005, respectively)	High
		Li, 2010 [39]	108	SLEDAI	***Target (source):*** CD64 (monocytes) ***Main findings:*** positive correlation (r=0.3023, p=0.0017)	High
		Oliveira, 2018 [28]	34	SLEDAI	***Target (source):*** Siglec1 (monocytes) ***Main findings:*** positive correlation (r=0.100, p=0.070)	High
		Rose, 2013 [188]	79	mSLEDAI-2K BILAG-2004	***Target (source):*** Siglec1 (monocytes) ***Main findings:*** mSLEDAI-2K: positive correlation (r=0.434, p<0.0001); BILAG-2004: positive correlation	Low

					(r=0.5409, p<0.0001)	
		Wilhem, 2016 [40]	22	SLEDAI	***Target (source):*** Siglec1 (pDCs), Siglec1 (monocytes)<br>***Main findings:*** positive correlation (Siglec1 (pDCs): r=0.550, p=0.008; Siglec1 (monocytes): r=0.620, p=0.003)	High
**Microarrays**	*Microarray modules*	Chiche, 2014 [46]	62	SELENA-SLEDAI	***Source:*** WB<br>***Main findings:*** significant correlation was observed between expression of M3.4 and M5.12 modules and the presence of a flare	High
		Mackay, 2016 [44]	43	SLEDAI	***Source:*** WB<br>***Main findings:*** no association between SLEDAI and IFN modules	High
	Microarray scores and clustering	Assassi, 2010 [47]	74	SLAM-R	***Source:*** WB<br>***Main findings:*** positive correlation (IFN score: p=0.010)	High
		Baechler, 2003 [49]	48	Number of SLE criteria	***Source:*** PBMC<br>***Main findings:*** positive association (IFN-high group showed higher number of SLE criteria compared to IFN-low group, p=0.004)	High
		Baechler, 2007 [48]	12	SLEDAI	***Source:*** WB<br>***Main findings:*** positive association (SLEDAI was higher in IFNhigh compared to IFNlow, p=0.050)	High
		Bauer, 2006 [193]	81	SLEDAI<br>SLAM-R<br>ESR	***Source:*** WB<br>***Main findings:***<br>SLEDAI: positive correlation (IFN score: r=0.430, p<0.050)<br>SLAM-R: positive correlation (IFN score: r=0.680, p<0.0001)<br>ESR: positive correlation (IFN score: r=0.520, p<0.010)	Low
		Higgs, 2011 [52]	261	SLEDAI<br>BILAG-2004	***Source:*** WB<br>***Main findings:***<br>SLEDAI: positive association (IFN score higher in SLEDAI>8 compared to SLEDAI<8, p<0.0001)<br>BILAG-2004: positive association (IFN score higher in BILAG>8 compared to BILAG<8, p=0.0006)	Unclear
		Kawasaki, 2011 [198]	12	SLEDAI (active vs inactive)	***Source:*** sorted cell populations (T-cells)<br>***Main findings:*** positive association (IFN-related genes were the most frequently annotated among genes differing	High

					between active and inactive SLE patients)	
		Kennedy, 2015 [63]	61<br>60<br>135<br>80<br>238	BILAG<br>SELENA-SLEDAI<br>SLEDAI<br>CLASI	***Source:*** PBMC<br>***Main findings:***<br>BILAG, SELENA-SLEDAI: no associations (ns)<br>CLASI damage score: positive association (IFN score high patients exhibited higher CLASI score compared to IFN score low patients, p=0.0209)<br>CLASI activity score: no associations (ns)	High
		Lauwerys, 2013 [58]	27	SLEDAI-2K	***Source:*** WB<br>***Main findings:*** no associations (ns)	High
		Petri, 2009 [199]	66	SLEDAI	***Source:*** WB<br>***Main findings:*** positive association (high IFNr group patients exhibited greater SLEDAI scores, $p=2.3\cdot10^{-4}$)	High
		Becker, 2013 [50]	15	SLEDAI	***Source:*** sorted cell populations<br>***Main findings:*** positive associations (a number of IFN-related differentially expressed genes was observed between active or inactive disease in B, T and myeloid cells)	High
		Bennet, 2003 [131]	30	SLEDAI	***Source:*** WB<br>***Main findings:*** positive association (10 IFN-related genes correlated with SLEDAI, all p<0.050)	High
		Biessen, 2008 [41]	47	SLEDAI	***Source:*** sorted cell populations (monocytes)<br>***Main findings:*** positive association (IFN-positive patients had higher SLEDAI than IFN-negative patients, p<0.010)	High
		Nikpour, 2008 [200]	269	SLEDAI-2K	***Source:*** WB<br>***Main findings:*** positive association (IFN-positive patients had higher SLEDAI-2K than IFN-negative patients, p<0.001)	High
		Sharma, 2015 [201]	42	SLEDAI	***Source:*** sorted cell populations (CD4+ T-cells, CD8+ T-cells, CD14+ monocytes and CD20+ B-cells)<br>***Main findings:*** no association (ns)	High
**qPCR**	*qPCR on individual ISG*	Abdel Galil, 2018 [6]	126	SLEDAI	***Source:*** WB<br>***Number of ISG:*** 1<br>***Main findings:*** IFNA expression higher in the highly active group than in the mild and moderately active groups	High
		Li, 2009	39	SLEDAI	***Source:*** PBMC	High

		[65]			***Number of ISG:*** 1<br>***Main findings:*** positive correlation (MXA: r=0.4814, p=0.0019)	
		Yuan, 2018 [66]	28	SLEDAI	***Source:*** WB<br>***Number of ISG:*** 1<br>***Main findings:*** positive correlation (ISG15: r=0.520, p=0.0059)	High
		Rodríguez-Carrio, 2019 [84]	75	SLEDAI	***Source:*** WB<br>***Number of ISG:*** 4<br>***Main findings:*** no associations (any ISG)	High
		Tang, 2008 [85]	144	SLEDAI-2K	***Source:*** PBMC<br>***Number of ISG:*** 1<br>***Main findings:*** positive correlation (LY6E: r=0.300, p<0.010)	High
		Zhuang, 2005 [18]	88	SLEDAI	***Source:*** WB<br>***Number of ISG:*** 1<br>***Main findings:*** no association (Mx1)	High
		Kawasaki, 2011 [198]	12	SLEDAI	***Source:*** sorted cell populations (T-cells)<br>***Number of ISG:*** 6<br>***Main findings:*** positive association (IFI35, JAK1, STAT1, IFITM1, JAK2, STAT2 expression was higher in active phase of SLE vs non active phase, all p<0.05)	High
	qPCR for IFN scores	Assassi, 2010 [47]	17	SLAM-R	***Source:*** WB<br>***Number of ISG:*** 3<br>***Main findings:*** positive correlation (IFN score: p=0.010)	High
		Braunstein, 2012 [68]	30	CLASI	***Source:*** PBMC<br>***Number of ISG:*** 5<br>***Main findings:*** positive correlation (IFN score: r=0.550, p=0.001)	Low
		Dominguez-Gutierrez, 2014 [71]	103	SLEDAI: active (SLEDAI >4) or inactive (SLEDAI ≤4)	***Source:*** leukocytes<br>***Number of ISG:*** 3<br>***Main findings:*** no associations	High
		El-Sherbiny, 2018 [74]	114	BILAG Overall, BILAG Skin, BILAG MSK,	***Source:*** WB<br>***Number of ISG:*** 31 (2 scores)<br>***Main findings:*** positive associations depended on organ	Unclear

				BILAG Haem	involvement:<br>IFN score A/IFN score B: OR (90% CI), p<br>Mucocutaneous: 1.71 (1.02-2.86), p=0.042 / 1.41 (1.08-1.84), p=0.012<br>Musculoskeletal: 0.79 (0.46-1.34), p=0.381 / 0.82 (0.63-1.08), p=0.165<br>Haematological: 1.90 (1.11-3.24), p=0.020 / 1.44 (1.09-1.90), p=0.012	
		Feng, 2017 [75]	35	SLEDAI<br>SLICC	***Source:*** WB<br>***Number of ISG:*** 5<br>***Main findings:***<br>SLEDAI: positive correlation (IFN score: r=0.690, p=0.001)<br>SLICC: no associations	Low
		Feng, 2015 [76]	69	SLEDAI	***Source:*** WB<br>***Number of ISG:*** 5<br>***Main findings:*** positive correlation (IFN score: r=0.310, p=0.014)	Low
		Feng, 2006 [92]	48	SELENA-SLEDAI	***Source:*** WB<br>***Number of ISG:*** 5<br>***Main findings:*** positive association (IFN score: higher in active disease (SLEDAI 5–12) or severe disease activity (SLEDAI >12) vs inactive or mild disease activity (SLEDAI 0-4): p=0.009 and p=0.002)	High
		Fu, 2008 [77]	68	SLEDAI	***Source:*** WB<br>***Number of ISG:*** 5<br>***Main findings:*** positive correlation (IFN score: p=0.023)	High
		Kennedy, 2015 [63]		SELENA-SLEDAI<br>CLASI	***Source:*** WB<br>***Number of ISG:*** 3<br>***Main findings:***<br>SELENA-SLEDAI: no associations<br>CLASI damage score: positive association (higher in IFNhigh vs IFNlow patients, p=0.020)<br>CLASI activity score: no associations	Low
		Kirou, 2005 [93]	73	SLEDAI-2K score<br>SLEDAI-2K	***Source:*** PBMC<br>***Number of ISG:*** 3<br>***Main findings:***	Unclear

				truncated<br>ESR<br>Number of SLE criteria<br>SDI	SLEDAI-2K: positive association (higher in IFNhigh vs IFNlow, p=0.003)<br>SDI: positive association (higher in IFNhigh vs IFNlow, p=0.0009)<br>ESR: positive association (higher in IFNhigh vs IFNlow, p=0.0105)<br>Number of ACR criteria: positive association (higher in IFNhigh vs IFNlow, p=0.001)	
		Landolt-Maricortena, 2009 [80]	94	SLEDAI-2K	***Source:*** PBMC<br>***Number of ISG:*** 5<br>***Main findings:*** positive correlation (r=0.253, p=0.0318)	High
		Liu, 2018 [81]	44	SLEDAI	***Source:*** PBMC<br>***Number of ISG:*** 4<br>***Main findings:*** positive association (IFNhigh patients showed higher SLEDAI vs IFNlow, p<0.050)	High
		Wahadat, 2018 [31]	23	SELENA-SLEDAI	***Source:*** sorted populations (monocytes)<br>***Number of ISG:*** 6<br>***Main findings:*** no association	High
		Merrill, 2017 [202]	98	SLEDAI<br>BILAG	***Source:*** WB<br>***Number of ISG:*** 1 score<br>***Main findings:***<br>SLEDAI: positive association (IFNhigh patients showed higher SLEDAI vs IFNlow, p=0.009)<br>BILAG: (IFNhigh patients showed higher SLEDAI vs IFNlow, p=0.040)	Unclear
		Sharma, 2015 [201]	42	SLEDAI	***Source:*** sorted populations (monocytes)<br>***Number of ISG:*** 46<br>***Main findings:*** no association	High
	qPCR for IFN-induced chemokines	Fu, 2008 [77]	68	SLEDAI	***Source:*** WB<br>***Number of ISG:*** 12<br>***Main findings:*** positive correlation (IFN chemokine score: r=0.340, p<0.005)	High
**DNA methylation**	*DNA methylation arrays*	Coit, 2013 [98]	18	SLEDAI	***Source:*** sorted populations (CD4+ T-cells)<br>***Main findings:*** no association	High
		Joseph, 2019 [99]	57	SLEDAI	***Source:*** PBMC<br>***Main findings:*** positive association (higher	High

					hypomethylation in several ISG in active SLE (SLEDAI>6) vs controls, but no differences between inactive SLE (SLEDAI<6) and controls	
		Absher, 2013 [101]	49	SLEDAI	***Source:*** sorted populations (CD4+ T-cells)<br>***Main findings:*** no association	High
	Pyrosequencing	Zhao, 2016 [107]	30	SLEDAI	***Source:*** WB<br>***Main findings:*** positive association (higher methylation in IFI44L promoter in remission vs patients with active disease, p<0.001 and p=0.036)	High
**Reporter cell assays**	*Reporter cell assays by qPCR*	Andrade, 2015 [108]	28	SLEDAI	***Assay:*** Mx1 expression<br>***Main findings:*** no association	High
		Nielsen, 2014 [27]	70 SLE-DK<br>68 SLE-SE<br>26 SLE-IFNa	SLEDAI<br>SLICC	***Assay:*** Mx1 expression<br>***Main findings:*** no association	High
		Oke, 2019 [191]	497	SLEDAI<br>SDI<br>SLAM	***Assay:*** MX1, EIF2AK2 and IFIT1 expression<br>***Main findings:***<br>SLEDAI: positive correlation (r=0.3, p<0.0001)<br>SDI: negative correlation (r=-0.13, p<0.0001)<br>SLAM: positive correlation (r=0.3, p<0.0001)	Low
	Reporter cell assays (other)	Dall'era, 2005 [116]	64	SLEDAI<br>ESR	***Assay:*** luciferase/ chemoluminiscence<br>***Main findings:***<br>SLEDAI: positive correlation (IFN activity: r=0.451, p<0.001)<br>ESR: positive correlation (IFN activity: r=0.481, p<0.001)	Low
		Kato, 2018 [117]	54	SLEDAI	***Assay:*** luciferase/ chemoluminiscence<br>***Main findings:*** positive correlation (IFN bioactivity: r=0.3034, p=0.0185; ISG-inducing activity: r=0.407, p=0.0009)	High
**Cytopathic effect assay**		Hervier, 2011 [120]	54	SLEDAI	Positive association (increased IFN activity in patients with active SLE vs inactive, p=0.004)	High

		Mathian, 2019 [187]	96	SELENA-SLEDAI	Positive association (IFN bioassay identified high disease activity: AUC 0.790; Sensitivity: 62.7 (51.3–73.0); Specificity: 94.0 (85.4–98.4); PPV: 92.9; NPV: 67.0)	High
		Rich, 1986 [203]	11	Disease activity	Positive association (IFN activity positivity associated with disease activity)	High
		Preble, 1982 [119]	86	Flare/remission	Positive association (52.3% samples during flare contained IFN (8-128 IU/ml) vs 25% samples during remission)	High
**Plaque-reducing assay**		Hooks, 1982 [123]	14	Clinical disease activity grades	Positive association (IFN titre positively associated with high disease activity)	High
		Hooks, 1979 [121]	28	Active/inactive disease groups	Positive association (IFN was found in the serum of 71% patients with active disease but only in 21% of those with inactive disease)	High
		Ytterberg, 1982 [124]	23	Disease activity stages (absent, possible, definite mild, definite moderate, definite severe)	Positive correlation (IFN levels: r=0.620, p<0.010)	High

Supplementary Table 7: Summary of the studies reporting assays analyzing associations between IFN assays and disease activity in RA.

Category	Sub-category	Reference	n	Disease activity measure	Results	Risk of bias
**Immunoassays**	*Immunoassays detecting IFNa protein*	Rodríguez-Carrio, 2014 [126]	120	DAS28<br>CRP<br>ESR	DAS28: positive association (higher in IFNhigh vs IFNlow patients, p<0.050)<br>CRP: no association<br>ESR: positive association (higher in IFNhigh vs IFNlow patients, p<0.050)	High
		Weix, 2013 [125]	7	DAS28-CRP	No association	High
	Immunoassays detecting IFNb protein	Weix, 2013 [125]	7	DAS28-CRP	No association	High
**Microarrays**	*Microarray scores and clustering*	Cantaert, 2010 [204]	21	DAS28<br>ESR<br>CRP	***Source:*** WB<br>***Main findings:*** no association	Unclear
		Higgs, 2011 [52]	45	Moderate (n=29) vs severe (n=16) stages	***Source:*** WB<br>***Main findings:*** no association	High
		Reynier, 2011 [132]	81	DAS28	***Source:*** WB<br>***Main findings:*** no association	Unclear
**qPCR**	*qPCR on individual ISG*	Weix, 2013 [125]	10	DAS28-CRP	***Source:*** PBMC<br>***Number of ISG:*** 6<br>***Main findings:*** no association (no differences in any of the genes between active or inactive disease)	High
	qPCR for IFN scores	Rodríguez-Carrio, 2019 [84]	98	DAS28<br>ESR	***Source:*** WB<br>***Number of ISG:*** 4 (cluster analysis)<br>***Main findings:***<br>DAS28: positive association (higher DAS28 in cluster I, low	Unclear

					expression of ISG, p<0.001)<br>ESR: positive association (higher ESR in cluster III, high expression of ISG, p=0.025)	
		Cooles, 2018 [138]	75	DAS28	***Source:*** WB<br>***Number of ISG:*** 6<br>***Main findings:*** positive correlation in early RA (IFN score: r=0.193, p=0.014) and established RA (IFN score: r=0.554, p=0.036)	Unclear
		De Jong, 2016 [70]	182	DAS28<br>ESR<br>CRP	***Source:*** WB<br>***Number of ISG:*** 19<br>***Main findings:*** no associations	Unclear
		Rodríguez-Carrio, 2017 [137]	98	DAS28	***Source:*** WB<br>***Number of ISG:*** 4<br>***Main findings:*** no association in very early RA (IFN score: r=-0.055, p=0.014), or bDMARD-naïve RA (IFN score: p=ns), and negative association in bDMARD patients (IFN score: r=-0.358, p=0.032)	High
		Thurlings, 2010 [205]	20<br>31	DAS28<br>ESR<br>CRP	***Source:*** PBMC<br>***Number of ISG:*** 3<br>***Main findings:*** no association	Unclear
**Reporter cell assays**	*Reporter cell assays (other)*	Shiozawa, 1986 [130]	40	Disease activity groups (active if arthritis w/pain and/or swelling in >4 joints by physician assessment + fatigue and >11 morning stiffness + increased	***Assay:*** luciferase/ chemoluminiscence<br>***Main findings:*** no association	High

				ESR and/or CRP; inactive if criteria for clinical remission)		
**Cytopathic effect assay**		Hertzog, 1988 [141]	12	Disease activity groups: inactive (MS<15min, no fatigue, no joint pain, no joint tenderness, no joint/tendon swelling, ESR<20) or active (no inactive criteria)	Negative association (OAS activity: higher in inactive RA vs controls, p<0.001)	High
**Plaque-reducing assay**		Arvin, 1984 [142]	65	Active vs inactive disease	Positive association (IFN activity: present in 10% inactive samples vs 47% active samples)	High

Supplementary Table 8: Summary of the studies reporting assays analyzing associations between IFN assays and disease activity in pSS.

Category	Sub-category	Reference	n	Disease activity measure	Results	Risk of bias
**Immunoassays**	*Immunoassays detecting IFN-induced proteins*	James, 2019 [206]	47	ESSDAI	Positive association (IFN-induced protein clusters positively associated with ESSDAI)	High
		Maria, 2014 [148]	24	ESSDAI	Positive correlation (MxA: r=0.650, p<0.010)	High
		Rose, 2016 [149]	25	ESSDAI	No association (IP-10: p=0.580)	High
**Flow cytometry**	*Flow cytometry to detect IFN-induced proteins*	Maria, 2014 [148]	28	ESSDAI	***Target (source):*** MxA (monocytes)<br>***Main findings:*** positive correlation (MxA: r=0.450, p=0.020)	High
		Rose, 2016 [149]	25	ESSDAI	***Target (source):*** Siglec1 (monocytes)<br>***Main findings:*** positive correlation (Siglec1: r=0.540, p=0.005)	High
**Microarrays**	*Microarray scores and clustering*	James, 2019 [206]	47	ESSDAI	***Source:*** WB<br>***Main findings:*** no associations between clusters and ESSDAI (p=0.190)	High
**qPCR**	*qPCR on individual ISG*	Kimoto, 2011 [152]	37	ESR<br>Schirmer test (mm/5 min)	***Source:*** WB<br>***Number of ISG:*** 1<br>***Main findings:***<br>ESR: positive correlation (IFI27: r=0.333, p<0.050)<br>Schirmer R: no association (IFI27: r=0.076, p=0.709)<br>Schirmer L: no association (IFI27: r=-0.085, p=0.677)	Low
	qPCR for IFN scores	Bodewes, 2018 [89]	86	ESSDAI<br>ClinESSDAI<br>CRP<br>ESR<br>Schirmer test<br>Saliva flow	***Source:*** WB<br>***Number of ISG:*** 3 scores<br>***Main findings:***<br>ESSDAI: no association (p=0.472)<br>ClinESSDAI: no association (p=0.929)<br>CRP: no association (p=0.567)<br>ESR: positive association (higher ESR in IFN-I/II high patients, p<0.001)	Low

Rodríguez-Carrio J, *et al. RMD Open* 2023; 9:e002864. doi: 10.1136/rmdopen-2022-002864

					Schirmer test: negative association (decreased in IFN-I/II high patients, p=0.028)<br>Saliva flow: no association (p=0.274)	
		Bodewes, 2019 [153]	77	ESSPRI	***Source:*** WB<br>***Number of ISG:*** 7<br>***Main findings:*** negative association (IFN signature associated with reduced scores of the pain and fatigue domain of the ESSPRI)	Unclear
		Brkic, 2013 [154]	38	ESSDAI<br>ESSPRI	***Source:*** monocytes<br>***Number of ISG:*** 5<br>***Main findings:*** positive correlation (IFN signature: r=0.458, p=0.003)	Low
		Maria, 2014 [148]	114	ESSDAI	***Source:*** monocytes<br>***Number of ISG:*** 5<br>***Main findings:*** positive association (IFNhigh patients showed higher ESSDAI than IFNlow patients, p<0.050)	Unclear
		Olsson, 2019 [207]	90	ESSDAI<br>ESSPRI	***Source:*** WB<br>***Number of ISG:*** 5<br>***Main findings:***<br>ESSDAI articular domain: positive association (p<0.010)<br>ESSPRI total score: positive association (p=0.040)<br>ESSPRI sicca score: positive association (p=0.030)<br>ESSPRI pain score: positive association (p=0.020)	Low

Rodríguez-Carrio J, *et al. RMD Open* 2023; 9:e002864. doi: 10.1136/rmdopen-2022-002864

Supplementary Table 9: Summary of the studies reporting assays analyzing associations between IFN assays and disease activity in SSc.

Category	Sub-category	Reference	n	Disease activity measure	Results	Risk of bias
**Immunoassays**	*Immunoassays detecting IFNa protein*	Eloranta, 2010 [159]	70	ESR<br>Digital ulcers	ESR: positive correlation (r=0.390, p<0.001)<br>Digital ulcers: positive association (p=0.029)	High
	Immunoassays detecting IFN-induced proteins	Eloranta, 2010 [159]	70	ESR	Positive correlation (IP-10 r=0.380, p=0.002; MCP-1: r=0.250, p=0.045; MIP-1A: r=0.460, p<0.001)	High
		Liu, 2013 [162]	266	mRSS<br>DLC<br>FVC	mRSS: positive association (chemokine score: r=0.210, p=0.002), no association (IP-10: r=0.16, p=0.014; I-TAC: r=0.04, p=0.205)<br>DLC: negative association (chemokine score: r=-0.18, p=0.008; I-TAC: r=-0.21, p=0.002), no association (IP-10: r=-0.10, p=0.160)<br>FVC: negative association (chemokine score: r=-0.17, p=0.012; I-TAC: r=-0.21, p=0.003), no association (IP-10: r=-0.08, p=0.272)	Low
**Microarrays**	*Microarray scores and clustering*	Assassi, 2010 [47]	74	mRSS<br>FVC<br>DLCO	***Source:*** WB<br>***Main findings:*** no association	Unclear
		Higgs, 2011 [52]	45	mRSS	***Source:*** WB<br>***Main findings:*** positive association (mRSS higher in gene signature positive vs negative groups, p=0.030)	High
		Bos, 2009 [165]	43	Digital ulcers	***Source:*** WB<br>***Main findings:*** positive association (greater number of digital ulcers in IFNhigh patients, p=0.050)	High
		Tan, 2006 [166]	18	mRSS	***Source:*** WB<br>***Main findings:*** no association	High
**qPCR**	*qPCR on individual ISG*	Airo, 2008 [168]	50	FVC<br>DLCO<br>Skin score	***Source:*** WB<br>***Number of ISG:*** 1<br>***Main findings:***	Unclear

				Digital ulcers	FVC: negative association (lower FVC in patients with high MxA levels, p=0.020)<br>DLCO: no association (p=0.070)<br>Skin score: no association (p=0.140)<br>Digital ulcers: positive association (higher number of digital ulcers in patients with high MxA levels, p=0.002)	
**Reporter cell assays**	*Reporter cell assays (other)*	Eloranta, 2010 [159]	70	ESR<br>Digital ulcers	***Assay:*** IFNa-inducing capacity of sera<br>***Main findings:***<br>ESR: positive association (higher ESR in patients with high IFNa-inducing capacity, p=0.022)<br>Digital ulcers: positive association (higher ESR in patients with high IFNa-inducing capacity, p=0.025)	Unclear

Supplementary Table 10: Summary of the studies reporting assays analyzing associations between IFN assays and disease activity in PM/DM.

Category	Sub-category	Reference	n	Disease activity measure	Results	Risk of bias
**Immunoassays**	*Immunoassays detecting IFNa protein*	Krol, 2011 [208]	19	MYOACT<br>MRI-VAS	MYOACT: no association<br>MRI-VAS: negative correlation (r=-0.580, p=0.0095)	High
		Sun, 2012 [174]	16	Skin lesion activity score<br>ESR	Skin lesion activity score: positive correlation (r=0.600, p=0.0147)<br>ESR: positive correlation (r=0.530, p=0.0329)	High
		Huard, 2017 [209]	42	CDASI	CDASI: no association (r=0.290, p=0.090)	Low
	Immunoassays detecting IFNb protein	Huard, 2017 [209]	42	CDASI	CDASI: positive correlation (r=0.540, p=0.0003)	Low
	Immunoassays detecting IFN-induced proteins	Huard, 2017 [209]	42	CDASI	Positive correlation (CXCL10: r=0.630, p<0.0001)	Low
		Baechler, 2007 [48]	12	Disease activity score (muscle strength testing, muscle enzyme elevation, ulcerative skin disease and patient's report of functional assessment)	Positive association (IP-10: elevated in active patients (no statistical analyses reported; MCP-1: r=0.550, p<0.050; MCP-2: r=0.600, p<0.010)	High
**Microarrays**	*Microarray scores and*	Baechler, 2007 [49]	12	Disease activity score	***Source:*** WB<br>***Main findings:*** positive correlation (IFN score: r=0.440,	High

	clustering			(muscle strength testing, muscle enzyme elevation, ulcerative skin disease and patient's report of functional assessment)	p=0.060; higher in active disease vs inactive disease, p=0.050)	
		Greenberg, 2012 [177]	21	MITAX	***Source:*** WB<br>***Main findings:*** positive association (IFN score higher in patients with high disease activity vs low or moderate disease activity, p<0.010)	High
		Higgs, 2011 [52]	45	Low / high disease activity	***Source:*** WB<br>***Main findings:*** positive association (IFN score higher in high disease activity vs low, p=0.004)	Unclear
		Walsh, 2007 [178]	36	Active disease / improving disease	***Source:*** WB<br>***Main findings:*** positive association (genes most upregulated in patients vs controls were highly down-regulated in improving disease group: IFI27, IFI44L, RSAD2, IFI44, OAS1, BIRC4BP)	Unclear
**qPCR**	*qPCR on individual ISG*	O'Connor, 2006 [179]	14	DAS muscle<br>DAS skin	***Source:*** PBMC<br>***Number of ISG:*** 1<br>***Main findings:***<br>DAS muscle: positive correlation (MxA: r=0.800, p<0.001)<br>DAS skin: no association (MxA: r=-0.208, p=0.476)	Low
	qPCR for IFN scores	Bilgic, 2009 [180]	37	Physician global VAS<br>MMT8 score	***Source:*** WB<br>***Number of ISG:*** 3<br>***Main findings:***<br>Physician global VAS: positive correlation (IFN signature: r=0.410, p=0.007)<br>MMT8 score: negative correlation (IFN signature: r=-0.48, p=0.002)	Unclear

		Ekholm, 2016 [184]	92	Physician global disease activity assessment<br>Patient global disease activity assessment<br>MMT8	***Source:*** WB<br>***Number of ISG:*** 8<br>***Main findings:***<br>Physician global disease activity assessment: no association (p=0.442)<br>Patient global disease activity assessment: no association (p=0.443)<br>MMT8: no association (p=0.250)	Unclear
		Huard, 2017 [209]	42	CDASI	***Source:*** WB<br>***Number of ISG:*** 10<br>***Main findings:*** positive correlation (IFN signature: r=0.610, p<0.001)	Low
	qPCR for IFN-induced chemokines	Bilgic, 2009 [180]	37	Physician global VAS<br>Muscle VAS score<br>MMT8 score	***Source:*** WB<br>***Number of ISG:*** 3<br>***Main findings:***<br>VAS score: positive correlation (r=0.61, p<0.0001)<br>Muscle VAS score: positive correlation (r=0.470, p<0.001)<br>MTT8 score: negative correlation (r=-0.440, p=0.002)	Low
**RNA-seq**	*RNA-seq*	Huard, 2017 [209]	42	CDASI	***Source:*** WB<br>***Main findings:*** positive association (K-means: optimal grouping of patients with a CDASI=12 (accuracy=0.952); cluster analysis: type I IFN-induced genes correlated with CDASI, patients with mild CDASI similar to controls)	Unclear
**Reporter cell assays**	*Reporter cell assays by qPCR*	Ekholm, 2016 [184]	40	Physician global disease activity assessment<br>Patient global disease activity assessment	***Assay:*** RSAD2, IFI44L and MX1 expression<br>***Main findings:*** no association	Unclear

				MMT8		
		Niewold, 2009 [182]	30	Muscle enzymes<br>DAS skin	***Assay:*** IFIT1, MX1, PRKR expression<br>***Main findings:***<br>Active patients: positive correlation (serum CPK: r=0.525, p=0.025, AST: r=0.705, p=0.002, and aldolase: r=0.447, p=0.036)<br>Inactive patients: negative correlation (DAS skin: r=-0.781, p=0.002)	Unclear

Supplementary Table 11: Summary of the studies reporting assays analyzing associations between IFN assays and prognosis (natural history) in SLE.

Category	Sub-category	Reference	n	Endpoint	Results	Risk of bias
**Immunoassays**	*Immunoassays detecting IFNa protein*	Rose, 2013 [188]	31	Flare (new BILAG A or B score at 180 days)	Positive association (High IFN serum levels (>20 pg/ml) predicted flare, p=0.0004)	Unclear
	Simoa for IFNa	Mathian, 2019 [187]	74	SELENA-SLEDAI flare instrument	Positive association (IFNa serum levels detect flare: AUC 0.840, sensitivity: 73.0 (61.4–82.7), specificity: 82.9 (72.5–90.6); PPV: 80.6; NPV 75.9)	Low
		Mathian, 2019b [35]	254	SELENA-SLEDAI flare instrument (1 year)	Positive association (elevated IFNa levels at baseline predicted higher risk of relapse, HR 5.5 (2.4-2.5), p<0.0001; isolated elevated IFNa levels predicted flare, HR 5.5 (1.7-18.1), p=0.005)	Low
	DELFIA for IFNa	Rose, 2017 [189]	26	Change in mSLEDAI-2K<br>Change in BILAG-2004<br>Flare (new BILAG A or B score at 180 days)<br>Remitting disease (improving of A or B in BILAG)	Change in mSLEDAI-2K: positive correlation (change in IFNa serum levels: r=0.447, p=0.001)<br>Change in BILAG-2004: positive correlation (change in IFNa serum levels: r=0.420, p=0.002)<br>Flare: positive association (AUC 0.56 (0.31-0.81), p=0.600, sensitivity: 41.67, specificity: 100, PPV: 100)<br>Remitting disease: positive association (AUC 0.84 (0.52-0.97), p=0.005, sensitivity: 62.50 specificity: 90.48, PPV: 76.00)	High
	Immunoassays detecting IFNb protein	Munroe, 2017 [192]	26	SELENA-SLEDAI flare instrument (6-12 weeks)<br>SELENA-SLEDAI at follow-up	SELENA-SLEDAI flare instrument: positive association (IFNb serum levels lower at baseline in patients without flare vs those with flare, p<0.010)<br>SELENA-SLEDAI at follow-up: positive correlation (IFNb: r=0.426, p=0.0691)	High
	Immunoassays detecting IFN-induced proteins	Rose, 2017 [189]	26	Change in mSLEDAI-2K<br>Change in BILAG-2004<br>Flare (new BILAG A or B score at 180	Change in mSLEDAI-2K: positive correlation (r=0.759, p<0.0001)<br>Change in BILAG-2004: positive correlation (r=0.504, p=0.0002)<br>Flare: positive association (AUC 0.75 (0.55-0.91), p=0.017, sensitivity: 50.00, specificity: 95.24, PPV:	High

				days)<br>Remitting disease (improving of A or B in BILAG)	75.00)<br>Remitting disease: positive association (AUC 0.75 (0.49-0.97), p=0.040, sensitivity: 62.50, specificity: 95.24, PPV: 71.43)	
		Munroe, 2017 [192]	26	SELENA-SLEDAI flare instrument (6-12 weeks)<br>SELENA-SLEDAI at follow-up	SELENA-SLEDAI flare instrument:<br>MCP1: positive association (lower at baseline in patients non-flare vs those with flare, p<0.001)<br>MCP3: positive association (lower at baseline in patients non-flare vs those with flare, p<0.001)<br>MIG: positive association (MIG lower at baseline in patients non-flare vs those with flare, p<0.001)<br>IP10: positive association (lower at baseline in patients non-flare vs those with flare, p<0.001)<br>MIP-1b: positive association (lower at baseline in patients non-flare vs those with flare, p<0.001)<br><br>SELENA-SLEDAI at follow-up<br>MCP1: positive correlation (r=0.452, p=0.0547)<br>MCP3: positive correlation (r=0.573, p=0.0178)<br>MIG: positive correlation (r=0.468, p=0.0468)<br>IP10: no association (r=0.426, p=0.0691)<br>MIP-1b: no association (r=0.407, p=0.0697)	High
		Bauer, 2009 [210]	267	Flare (1 year)	Positive association (IFN-chemokine score rise at the time of flare (p<0.001) and decreased as disease remitted (p<0.001); IFN-chemokine score predicted flare in patients with SLEDAI<4 at baseline, HR 2.52 (1.63-4.09), p<0.0001)	Low
**Flow cytometry**	*Flow cytometry to detect IFN-induced proteins*	Rose, 2017 [189]	26	Change in mSLEDAI-2K<br>Change in BILAG-2004<br>Flare (new BILAG A or B score at 180 days)<br>Remitting disease (improving of A or B	***Target (source):*** Siglec1 (monocytes)<br>***Main findings:***<br>Change in mSLEDAI-2K: positive correlation (r=0.463, p<0.001)<br>Change in BILAG-2004: positive correlation (r=0.448, p<0.001)<br>Flare: positive association (AUC 0.78 (0.57-0.96), p=0.008, sensitivity: 83.33, specificity: 90.48, PPV: 71.43)	High

				in BILAG)	Remitting disease: positive association (AUC 0.75 (0.42-1.00), p=0.040, sensitivity: 75.00, specificity: 95.24, PPV: 85.71)	
**Microarrays**	*Microarray scores and clustering*	Hoffman, 2017 [53]	1760	SELENA-SLEDAI flare instrument, 1 year	***Source:*** WB<br>***Number of ISG:*** 34<br>***Main findings:*** positive association (IFN score: RR 5.6, p=0.0015 (ILLUMINATE1 cohort); RR 5.9, p=0.0002 (ILLUMINATE2 cohort))	Low
		Mackay, 2016 [44]	27	SELENA-SLEDAI flare instrument (1 year)	***Source:*** WB<br>***Number of ISG:*** 2 IFN scores (A/B)<br>***Main findings:*** positive association (IFN score higher at baseline in patients with flare, p=0.006)	Unclear
**qPCR**	*qPCR for IFN scores*	El-Sherbiny, 2018 [74]	60	Flare occurrence<br>Organ involvement	***Source:*** PBMC<br>***Number of ISG:*** 1<br>***Main findings:***<br>Flare occurrence: positive association (high IFN score A, p=0.042), no association (IFN score B, p=0.343)<br>Organ involvement: no association (IFN score A, p=0.124), positive association (high IFN score B, p=0.037)	High
		Feng, 2006 [92]	48	SELENA-SLEDAI flare instrument	***Source:*** WB<br>***Number of ISG:*** 6<br>***Main findings:***<br>IFN score: positive association (higher in patients with severe flare vs those mild/moderate (p=0.020) or stable (p=0.020))<br>LY6E: positive association (higher in patients with severe flare vs stable, p=0.020)	High
		Landolt-Maricortena, 2009 [80]	27	Change in disease activity (1 year)	***Source:*** WB<br>***Number of ISG:*** 5<br>***Main findings:*** no association (IFN score: r=-0.022, p=0.910)	Unclear
		Md Yusof, 2018 [82]	105	CTD development (1 year)	***Source:*** PBMC<br>***Number of ISG:*** 2 IFN scores (A/B)<br>***Main findings:*** positive association (IFN scores higher in at-risk progressors vs non-progressors, p=0.018 and	Low

					p<0.001)	
		Steiman, 2015 [211]	102	Clinical quiescence: SACQ (2-year without clinical activity but persistent serologic activity), SQCQ (inactive controls, 2-year without clinical or serologic activity) and SACA (clinical activity requiring use of GC and/or immunosuppressive agents)	***Source:*** WB<br>***Number of ISG:*** 5<br>***Main findings:*** positive association (IFIT1, ISG15, LY6E, MX1 and OAS1 expression were higher in SACA vs SACQ, p=0.0034, p=0.044, p=0.0014, p=0.027 and p=0.0047; IFN score was higher in SACA vs SACQ, p=0.0025)	Unclear
**Nanostring**	*Nanostring*	Wither, 2017 [97]	23 UCTD 19 ANA+	SARD development (1 year)	***Source:*** WB<br>***Number of ISG:*** 5<br>***Main findings:*** no association	High
**DNA methylation**	*DNA methylation arrays*	Ulf-Moller, 2018 [104]	15 twin pairs	Flare (2 years)	***Source:*** sorted populations (CD4+ T-cells, monocytes, granulocytes, B cells)<br>***Main findings:*** positive association (Several ISG exhibit differential methylation, mostly hypomethylation, in twins with flare vs those in remission in CD4+ T-cells (8), monocytes (8), granulocytes (9), and B-cells (8))	High
**Reporter cell assays**	*Reporter cell assays by qPCR*	Andrade, 2015 [108]	28	Poor outcomes of pregnancy	***Assay:*** Mx1 expression<br>***Main findings:*** positive association (MX1 expression was higher in patients who developed preeclampsia vs those with other (p<0.006) or without outcomes (p<0.040))	High
**Cytopathic effect assay**		Mathian, 2019 [187]	74	SELENA-SLEDAI flare instrument	Positive association (increased IFN bioactivity predicted flare: AUC 0.780, sensitivity: 63.5 (51.5–74.4), specificity: 88.2 (78.7–94.4), PPV: 83.9, NPV: 71.3)	Low

Supplementary Table 12: Summary of the studies reporting assays analyzing associations between IFN assays and prognosis (natural history) in RA.

Category	Sub-category	Reference	n	Endpoint	Results	Risk of bias
**Microarrays**	*Microarray scores and clustering*	van Baarsen, 2010 [212]	109	RA development (1 year)	***Source:*** WB<br>***Number of ISG:*** 52 gene sets (cluster analysis)<br>***Main findings:*** positive association (IFN signature associated with RA development, OR 21 (95% CI: 2.8–156.1), p=0.003)	High
**qPCR**	*qPCR for IFN scores*	Cooles, 2018 [138]	632	Disease activity (DAS28) at 6 months	***Source:*** WB<br>***Number of ISG:*** 5<br>***Main findings:*** positive correlation (r=0.319, p=0.002)	High
		Lubbers, 2013 [213]	115<br>73	RA development	***Source:*** WB<br>***Number of ISG:*** 7<br>***Main findings:*** positive association (cohort 1: 15/25 IFNhigh patients developed RA vs 29/90 IFNlow, p=0.001, AUC 0.602 (95% CI 0.491-0.714, p=0.066; cohort 2: IFN score higher in presymptomatic individuals who developed RA vs controls, p=0.002; IFNhigh status: 14/23 RA, 15/25 presymptomatic, 10/45 HC, p=0.004)	High

Supplementary Table 13: Summary of the studies reporting assays analyzing associations between IFN assays and prognosis (natural history) in SSc.

Category	Sub-category	Reference	n	Endpoint	Results	Risk of bias
**Microarrays**	*Microarray modules*	Assassi, 2019 [164]	62	Increase in FVC%, 26 months<br>Change in mRSS, 26 months	***Source:*** WB<br>***Main findings:***<br>Increase in FVC%: negative correlation (M1.2: r=-0.430, p=0.009; M3.4: r=-0.450, p=0.007)<br>Change in mRSS: no associations	High

Supplementary Table 14: Summary of the studies reporting assays analyzing associations between IFN assays and prognosis (response to treatments) in SLE.

Category	Sub-category	Reference	n	Treatment [target]	Endpoint	Results	Risk of bias
**Microarrays**	*Microarray scores and clustering*	Hoffman, 2017 [53]	1760	Tabalumab [B cell activating factor]	SRI (52 weeks)	***Source:*** WB<br>***Number of ISG:*** 34<br>***Main findings:*** no association	Unclear
**qPCR**	*qPCR for IFN scores*	Furie, 2017 [214]	304	Anifrolumab (300 mg, n=99 and 1000 mg, n=103) [IFN alpha receptor subunit 1]	SRI(4) including GC taper (52 weeks)<br>SRI(4) excluding GC taper (52 weeks)<br>SRI(7) (52 weeks)<br>BICLA (52 weeks)	***Source:*** WB<br>***Number of ISG:*** 4<br>***Main findings:*** positive association (greater effect size in IFNhigh patients)<br>Anifrolumab 300 mg<br>SRI(4) including GC taper: IFNhigh OR 4.30 (2.34-7.91), p<0.001; IFNlow 1.47 (0.55-3.93), p=0.514<br>SRI(4) excluding GC taper: IFNhigh OR 2.98 (1.69-5.24), p<0.001; IFNlow 2.07 (0.77-5.53), p=0.225<br>SRI(7): IFNhigh OR 4.59 (2.26-9.33), p<0.001; IFNlow 0.94 (0.29-3.04), p=0.930<br>BICLA: IFNhigh OR 3.65 (2.02-6.60), p<0.001; IFNlow 3.19 (1.16-8.73), p=0.059<br>Anifrolumab 1000 mg<br>SRI(4) including GC taper: IFNhigh OR 2.52 (1.37-4.64), p=0.013; IFNlow 0.89 (0.34-2.35), p=0.849<br>SRI(4) excluding GC taper: IFNhigh OR 2.33 (1.34-4.04), p=0.012; IFNlow 0.85 (0.34-2.12), p=0.763<br>SRI(7): IFNhigh OR 2.65 (1.29-5.34), p=0.26; IFNlow 0.81 (0.25-2.61), p=0.763<br>BICLA: IFNhigh OR 2.41 (1.34-4.35), p=0.014; IFNlow 1.38 (0.51-3.70), p=0.596	Low
		Kalunian, 2016 [215]	238	Rontalizumab [IFN alpha protein]	BILAG index response (24 weeks)<br>SRI4 (24	***Source:*** WB<br>***Number of ISG:*** 3<br>***Main findings:*** positive association (SRI response was higher and steroid use was lower in the IFNlow	Unclear

					weeks)	rontalizumab treated patients)<br><br>BILAG response index:<br>IFNhigh: placebo 21/55 (38.2%) vs rontalizumab 53/123 (43.1%), 5.2% (−7.6 - 18.1%), p=0.510<br>IFNlow: placebo 12/24 (50%) vs rontalizumab 18/33 (54.5%), 4.5% (−17.7 - 26.5%), p=0.790<br>SRI4:<br>IFNhigh: placebo 26/55 (47.3%) vs rontalizumab 55/123 (44.7%), −2.3% (−15.5 - 10.9%), p=0.780<br>IFNlow: placebo 10/24 (41.7%) vs rontalizumab 24/33 (72.7%), 31.1% (8.9 - 51.0%) p=0.030	
		Merrill, 2018 [216]	201	Anifrolumab 300 mg [IFNA receptor subunit 1]	SLEDAI-2K-defined resolution of rash<br>BILAG-defined improvement in rash<br>Improvement in CLASI<br>Resolution in SLEDAI-2K-defined arthritis<br>Improvement in BILAG-defined arthritis<br>Mean change in swollen and tender joint counts	***Source:*** WB<br>***Number of ISG:*** 4<br>***Main findings:*** positive association (greater effect in IFNhigh patients)<br>SLEDAI-2K-defined resolution of rash:<br>IFNhigh: placebo 7/65 (10.8) vs anifrolumab 33/67 (49.3), OR 8.08 (3.72-17.52), p<0.001<br>IFNlow: placebo 6/23 (26.1) vs anifrolumab 6/21 (28.6), OR 1.40 (0.43-4.53), p=0.639<br>BILAG-defined improvement in rash:<br>IFNhigh: placebo 17/64 (26.6) vs anifrolumab 35/61 (57.4), OR 3.78 (2.00-7.14), p<0.001<br>IFNlow: placebo 7/21 (33.3) vs anifrolumab 13/21 (61.9), OR 3.93 (1.28-12.04), p=0.044<br>Improvement in CLASI:<br>IFNhigh: placebo 21/67 (31.3) vs anifrolumab 43/70 (61.4), OR 3.67 (2.01-6.71), p<0.001<br>IFNlow: placebo 9/22 (40.9) vs anifrolumab 14/22 (63.3), OR 2.70 (0.94-7.80), p=0.123<br>Resolution in SLEDAI-2K-defined arthritis:<br>IFNhigh: placebo 29/73 (39.7) vs anifrolumab 41/73 (56.2), OR 2.11 (1.20-3.71), p=0.030<br>IFNlow: placebo 13/26 (50.5) vs anifrolumab 14/24 (58.3), OR 1.41 (0.55-3.64), p=0.547	Unclear

						Improvement in BILAG-defined arthritis:<br>IFNhigh: placebo 34/72 (47.2) vs anifrolumab 47/71 (66.2), OR 2.39 (1.34-4.27), p=0.013<br>IFNlow: placebo 13/23 (56.5) vs anifrolumab 18/23 (78.3), OR (2.91 (0.97-8.72), p=0.110<br>Mean change in swollen and tender joint counts:<br>IFNhigh: placebo -3.0(5.8) vs anifrolumab -4.9(6.1), mean difference (SE) –1.9(0.8), p=0.014<br>IFNlow: placebo -4.5(6.1) vs anifrolumab -7.4(6.3), mean difference (SE), –2.1(1.4), p=0.140	
		Petri, 2013 [217]	213	Sifalimumab (0.3-10 mg) [IFN alpha protein]	Mean reduction in SELENA-SLEDAI	***Source:*** WB<br>***Number of ISG:*** 21<br>***Main findings:*** no association (greater but non-significant SELENA-SLEDAI reduction vs placebo: mean change: -2.6 vs -2.4)	Unclear

Supplementary Table 15: Summary of the studies reporting assays analyzing associations between IFN assays and prognosis (response to treatments) in RA.

Category	Sub-category	Reference	n	Treatment [target]	Endpoint	Results	Risk of bias
**Immunoassays**	*Immunoassays detecting IFNa protein*	Rodríguez-Carrio, 2014 [126]	26	Anti-TNF (various agents) [TNF protein]	EULAR clinical response (12 weeks)	Positive association (lower effect in IFNhigh patients (0% good response, 41.6% moderate response, 58.3% no response) compared to IFNlow (35% good response, 35% moderate response, 0% no response).	High
**Microarrays**	*Microarray scores and clustering*	Cantaert, 2010 [204]	21	Infliximab [TNF protein]	EULAR clinical response (24 weeks)	***Source:*** WB<br>***Number of ISG:*** 43<br>***Main findings:*** no association	High
		Reynier, 2011 [132]	22	Anti-TNF (various agents) [TNF protein]	EULAR clinical response (24 weeks)	***Source:*** WB<br>***Number of ISG:*** 35<br>***Main findings:*** no association	High
		Sanayama, 2014 [218]	40<br>20	Tocilizumab [anti-IL-6R]	Clinical response by physician global assessment	***Source:*** PBMC<br>***Number of ISG:*** number of differentially expressed genes (DEG)<br>***Main findings:*** positive association (409 probes fulfilled criteria for DEG (>1.5 FC): IFI6 (higher in responders, 0.006, AUC 0.693), MX2 (higher in responders, p=0.004, AUC 0.920), OASL (higher in responders, p=0.024, AUC 0.627; IFI6, MX2 and OASL score: AUC 0.853, sensitivity 80.0, specificity 80.0, PPV 92.0, NPV 57.0)	High
		van Baarsen, 2010 [212]	15	Infliximab [TNF protein]	EULAR clinical response (24 weeks)	***Source:*** WB<br>***Number of ISG:*** 15<br>***Main findings:*** positive association (increased IFN score at 4 weeks was associated with poor clinical response, p=0.022)	High
		Vosslamber,	13	Rituximab	Change in	***Source:*** WB	High

		2011 [219]		[CD20]	DAS: >1.2/<1.2 (24 weeks) EULAR clinical response (24 weeks)	***Number of ISG:*** number of differentially expressed genes, gene clusters<br>***Main findings:***<br>Change in DAS: positive association (increased IFN response during treatment was associated with good or moderate response, p=0.018)<br>EULAR clinical response: positive association (equivalent results)	
**qPCR**	*qPCR for IFN scores*	Raterman, 2012 [220]	27	Rituximab [CD20]	Responders (dDAS>1.2) vs non-responders (dDAS<1.2) (24 weeks)	***Source:*** WB<br>***Number of ISG:*** 8<br>***Main findings:*** positive association (IFN score at baseline was negatively correlated with dDAS<1.2, OR: 0.25 (0.09-0.70), p=0.008; IFN score at baseline was lower in responders vs non-responders, AUC 0.820, p=0.0074)	Low
		Cooles, 2018 [138]	32	MTX, HCQ	EULAR clinical response (24 weeks) Need of additional GC doses (24 weeks)	***Source:*** WB<br>***Number of ISG:*** 5<br>***Main findings:***<br>EULAR clinical response: positive association (IFN score at baseline was negatively associated with good response, p=0.044)<br>Need of additional GC doses: positive association (IFN score at baseline was negatively associated with additional GC administration, p=0.0003)	Low
		De Jong, 2015 [221]	40	Rituximab [CD20]	Change dDAS>1.2 (24 weeks)	***Source:*** WB<br>***Number of ISG:*** 8<br>***Main findings:*** positive association (higher IFN score at baseline was associated with non-response, AUC 0.828 (0.699-0.957), p<0.001)	Unclear
		Rodríguez-Carrio, 2017 [137]	18<br>13	GC+MTX<br>Anti-TNF (various agents) [TNF protein]	EULAR clinical response (12 and 24 weeks) DAS28 (24 and 52	***Source:*** WB<br>***Number of ISG:*** 4<br>***Main findings:***<br>VERA cohort (n=18)<br>EULAR clinical response: positive association (higher IFN score was associated with poor clinical response, p=0.006, AUC 0.917 (0.782 – 1.000),	Unclear

					weeks)	p=0.004)<br>DAS28: positive correlation (24 weeks: r=0.620, p=0.008; 52 weeks: r=0.552, p=0.041)<br><br>Anti-TNF (n=13)<br>DAS28: positive association (IFN score at baseline predicted higher DAS28 at 12 weeks after adjusting for confounders, B (95% CI): 0.577 (0.052-1.102), p=0.035)	
		Thurlings, 2010 [205]	20<br>31	Rituximab [CD20]	EULAR clinical response, (12 and 24 weeks)<br>DAS28 decrease (12 and 24 weeks)	***Source:*** PBMC<br>***Number of ISG:*** 3<br>***Main findings:***<br>Cohort 1 (n=20)<br>EULAR clinical response: no association<br>DAS28 decrease: no association<br><br>Cohort 2 (n=31)<br>EULAR clinical response: positive association (IFNhigh patients less likely to achieve a good response, 12 weeks p=0.043, 24 weeks: p=0.059)<br>DAS28 decrease: positive association (lower reduction in IFNhigh patients, 12 weeks: p=0.008, 24 weeks: p=0.027)<br><br>Pooled analysis: positive association (high IFN signature associated with poor clinical response and DAS28 decrease)	Unclear
**RNA-seq**	*RNA-seq*	Wright, 2015 [139]	30	Anti-TNF (various agents) [TNF protein]	EULAR clinical response (12 weeks)<br>Change DAS28>1.2 (12 weeks)<br>Change in DAS28 (12	***Source:*** neutrophils<br>***Number of ISG:*** 128<br>***Main findings:***<br>EULAR clinical response: positive association (high IFN score at baseline predicted good clinical response, AUC 0.760)<br>Change DAS28>1.2: positive association (high IFN score more likely to have a change DAS28<1.2, AUC 0.640)	High

					weeks)	Change in DAS28: positive correlation (r=0.210, p=0.020)	
**Reporter cell assays**	*Reporter cell assays by qPCR*	Thurlings, 2010 [205]	20<br>31	Rituximab [CD20]	EULAR clinical response (12 and 24 weeks)<br>DAS28 decrease (12 and 24 weeks)	***Assay:*** serum IFN bioactivity<br>***Main findings:***<br>EULAR clinical response: no association<br>DAS28 decrease: no association	High
		Mavragani, 2010 [140]	47	Anti-TNF (various agents) [TNF protein]	EULAR clinical response (14 weeks)	***Assay:*** serum IFN bioactivity<br>***Main findings:*** positive association (high IFN bioactivity associated with a good clinical response, OR 1.36 (1.05-1.76), p=0.027)	High
		Muskardin, 2016 [222]	32<br>92	Anti-TNF (various agents) [TNF protein]	EULAR clinical response (12 weeks)	***Assay:*** serum IFN bioactivity<br>***Main findings:***<br>Test cohort (n=32)<br>EULAR clinical response: positive association (IFNb/a ratio >1.3 associated with a lack of EULAR clinical response, p=0.010)<br><br>Validation cohort (n=92)<br>EULAR clinical response: positive association (IFNb/a ratio >1.3 associated with no response at 12 weeks, OR 6.67 (1.37-32.55), p=0.018)	High

Supplementary Table 16: Summary of the studies reporting assays analyzing associations between IFN assays and prognosis (response to treatments) in PM/DM.

Category	Sub-category	Reference	n	Treatment [target]	Endpoint	Results	Risk of bias
**Immunoassays**	*Immunoassays detecting IFNa protein*	Reed, 2012 [223]	51	Immunomodulatory treatment (AZA, MTX, MMF, HCQ and GC)	Change in global VAS Change in muscle VAS Change in extra-skeletal VAS	Change in global VAS: no association (IFNa: r=-0.140, p=0.460) Change in muscle VAS: no association (IFNa: r=-0.17, p=0.380) Change in extra-skeletal VAS: no association (IFNa: r=-0.220, p=0.260)	High
	Immunoassays for IFN-induced proteins	Reed, 2012 [223]	51	Immunomodulatory treatment (AZA, MTX, MMF, HCQ and GC)	Change in global VAS Change in muscle VAS Change in extra-skeletal VAS	Change in global VAS: negative correlation (MIP1a: r=-0.190, p=0.320, IP-10: r=-0.610, p<0.001; IFN-chemokine score: r=-0.590, p<0.001) Change in muscle VAS: no association (MIP1a: r=-0.190, p=0.330); negative correlation (IP-10: r=-0.510, p<0.001; IFN-chemokine score: r=-0.500, p<0.0001) Change in extra-skeletal VAS: no association (MIP1a: r=-0.090, p=0.670), negative correlation (IP-10: r=-0.640, p<0.001; IFN-chemokine score: r=-0.620, p<0.001)	High
		Reed, 2015 [224]	177	Rituximab [CD20]	Changes in muscle VAS (16 weeks) Physician global VAS scores (16	Changes in muscle VAS: positive association (IFN-chemokine score at baseline predicted larger improvements in muscle VAS, depending on the autoantibody profile) Physician global VAS scores: no association (p=0.090)	High

					weeks)		
**qPCR**	*qPCR for IFN scores*	Reed, 2012 [223]	51	Immunomodulatory treatment (AZA, MTX, MMF, HCQ and GC)	Change in global VAS<br>Change in muscle VAS<br>Change in extra-skeletal VAS	***Source:*** WB<br>***Number of ISG:*** 3<br>***Main findings:***<br>Change in global VAS: negative correlation (IFN score: r=-0.430, p=0.003)<br>Change in muscle VAS: negative correlation (IFN score: r=-0.560, p<0.0001)<br>Change in extra-skeletal VAS: negative correlation (IFN score: r=-0.300, p=0.048)	High

Supplementary Table 17: Summary of the studies reporting assays analyzing associations between IFN assays and prognosis (response to treatments) in pSS.

Category	Sub-category	Reference	n	Treatment	Endpoint	Results	Risk of bias
**qPCR**	*qPCR for IFN scores*	Bodewes, 2019 [153]	37	HCQ	Change in ESR (12, 24 and 48 weeks)<br>Change in IgG and IgM levels (12, 24 and 48 weeks)<br>Change in ESSDAI and ESSPRI (12, 24 and 48 weeks)<br>Change in ocular or oral dryness (12, 24 and 48 weeks)<br>Change in SF-36 or HAD scales (12, 24 and 48 weeks)	***Source:*** WB<br>***Number of ISG:*** 5<br>***Main findings:***<br>Change in ESR: no association<br>Change in IgG and IgM levels: no association<br>Change in ESSDAI and ESSPRI: no association<br>Change in ocular or oral dryness: no association Change in SF-36 or HAD scales: no association	High
		Quartuccio, 2017 [225]	12	Belimumab [B lymphocyte	Change in ESSDAI (28 and 52	***Source:*** PBMC<br>***Number of ISG:*** 3<br>***Main findings:*** no association	High

				stimulator]	weeks)		

Supplementary Table 18: Summary of the studies reporting assays analyzing responsiveness to change of IFN assays in SLE.

Category	Sub-category	Reference	n	Treatment [target]	Timepoints	Results	Risk of bias
**Immunoassays**	*Immunoassays detecting IFNa protein*	Bengtsson, 2000 [185]	30	Usual care	Pre-flare / flare / post-flare visits<br>Intervals:<br>Pre-flare vs flare: median 4 months (1-21)<br>Post-flare 1 vs flare: 2 months (0.5-9)<br>Post-flare 2 vs post-flare 1: 2.5 months (1-5)	Changes observed: IFNa levels increased at flare vs pre-flare (p=0.005), post-flare 1 (p=0.0008) and post-flare 2 (p=0.002)	Unclear
		Fragoso-Loyo, 2012 [8]	20	Usual care	6 months	No change (IFNa: T0 52.4 (3.2–1074.4) vs T6 77.6 (3.2–2321.4), p=0.200)	High
		Willis, 2012 [14]	35	HCQ	Consecutive visits (not specified)	No change (IFNa decreased (non-significant) by 33.5%: 573.06 to 381.03, p=0.2507)	Low
	Immunoassays for IFN-induced proteins	Bauer, 2009 [210]	73	Usual care	Consecutive visits from patients who exhibited a reduction in disease	***Target:*** 3 chemokines (CXCL10, CCL19, CCL2)<br>***Main findings:*** changes observed (IFN-chemokine score drop at disease activity reduction, p<0.0010; Chemokine score increased at interim visits (42% increase) and at flare visits (53% increase))	Low

					activity (decrease in SLEDAI ≥3) along 1 year		
		Connelly, 2018 [196]	109	Usual care	Consecutive visits (median length follow up = 3.2 years, median clinical visits = 7)	***Target:*** 3 chemokines (CXCL10, CCL19, CCL2)<br>***Main findings:*** changes observed in association with disease activity (increase of one unit in IFN-CK score was significantly associated with an increase in SLEDAI-2K of 0.7 (RC = 0.73, (95% CI: 0.12, 1.43) p = 0.02))	Unclear
		Casey, 2018 [90]	304	Anifrolumab (3, 300 or 1000 mg) [IFNA receptor subunit 1]	1 year	***Target:*** 134 chemokines (CXCL10, CCL19, CCL2)<br>***Main findings:*** changes observed (Anifrolumab suppressed 11/27 proteins elevated in patients with SLEDAI-2K>10 and 5/7 proteins elevated in patients with CLASI>10)	Unclear
**Flow cytometry**		Alexander, 2015 [226]	12	Bortezomib (21-day cycles) [proteasome]	2 (mean) 21-day cycles	***Target (source):*** Siglec1 (monocytes)<br>***Main findings:*** changes observed (Siglec1 expression decreased upon treatment, baseline: 42.3 vs post-treatment: 18.8, p<0.001)	Low
		Li, 2010 [39]	4	High dose IV prednisolone (1 g/day)	3 days	***Target (source):*** CD64 (monocytes)<br>***Main findings:*** changes observed (CD64 decreased at day 3, p=0.006)	High
**Microarrays**	*Microarray modules*	Chiche, 2014 [46]	29	Usual care	At least three visits: median follow-up: 8.3 (2–28) months, median interval	***Source:*** WB<br>***Main findings:*** changes observed (module M1.2 very stable over time within individual patients (coefficient of variation=0.05); module M3.4: greater variation (CV=0.39); module M5.12: much greater variation (CV=0.91))	Low

					between visits:3.2 (0.5–19)		
		Petri, 2019 [227]	243	Usual care	2 or more clinical visits (not specified)	***Source:*** WB<br>***Main findings:*** no changes (IFN modules highly stable over time: M1.2 (ICC 0.88), M3.4 (ICC 0.79), M5.12 (ICC 0.75))	Unclear
	Microarray scores and clustering	Petri, 2009 [199]	11	Usual care	2-3 longitudinal visits (not specified)	***Source:*** WB<br>***Number of ISG:*** 4<br>***Main findings:*** no changes (IFN scores remained constant, despite significant changes in disease activity)	Unclear
		Bennet, 2003 [131]	3	IV glucocorticoids 30 mg/kg/day	3 days	***Source:*** PBMC<br>***Number of ISG:*** several ISG<br>***Main findings:*** changes observed (significant downregulation of several ISG, overall p<0.001; interferon signature decreased, but granulocyte-related signature not affected)	High
		Hoffman, 2017 [53]	1760	Tabalumab [B cell activating factor]	1 year	***Source:*** WB<br>***Number of ISG:*** 34 ISG<br>***Main findings:*** no change (no change in IFN score over the 52-week duration in the placebo or tabalumab group; IFN signature was not down-modulated in patients who showed improvement in disease activity)	Unclear
		Kawasaki, 2011 [198]	12	Prednisolone initiation or increase in doses	Before (active phase) and after (inactive phase) of steroid treatment	***Source:*** CD3+ T-cells<br>***Number of ISG:*** signaling pathways and differentially expressed genes (DEG)<br>***Main findings:*** changes observed (711 DEG detected; interferon pathway ranked the top, with higher levels during the active phase)	Low
		Lauwerys, 2013 [58]	27	IFNa-kinoid	168 days	***Source:*** WB<br>***Number of ISG:*** 21	Unclear

						Main findings: changes observed (21 ISG changed from day 0 to day 122 and 168; IFNa-kinoid reduced the expression of ISG in IFNpositive patients)	
		Petri, 2013 [217]	161	Sifalimumab (0.3-10 mg/kg) every other 2 weeks [IFNa protein]	26 weeks under treatment + 24 weeks follow up	***Source:*** WB<br>***Number of ISG:*** 21<br>***Main findings:*** changes observed (sustained inhibition with sifalimumab in IFNhigh patients, maximum average inhibition of 38.7% in the 1 mg/kg group)	Low
**qPCR**	*qPCR for IFN scores*	Kennedy, 2015 [63]	61	Rontalizumab [IFNa protein]	36 weeks	***Source:*** PBMC<br>***Number of ISG:*** 3<br>***Main findings:*** changes observed (rontalizumab decreased IFN score over time vs placebo, IFNlow patients exhibited a greater effect but downregulation in IFNhigh did not reach IFNlow levels)	Unclear
		Dominguez-Gutierrez, 2014 [71]	60	Usual care	Follow-up of patients with at least 2 visits (not specified)	***Source:*** PBMC<br>***Number of ISG:*** 3<br>***Main findings:*** changes observed (patients can be classified into increasing and decreasing IFN score groups)	High
		Liu, 2018 [81]	7	Not specified	Not specified	***Source:*** PBMC<br>***Number of ISG:*** 4<br>***Main findings:*** changes observed (IFN-I score decrease (p<0.050) upon treatment or follow up (duration and treatment not disclosed))	High
		Aranow, 2015 [228]	48	Vitamin D (0, 2000 IU or 4000 IU)	12 weeks	***Source:*** WB<br>***Number of ISG:*** 3<br>***Main findings:*** no change (IFN score was unchanged upon vitamin D treatment)	Low
		Fu, 2008 [77]	4	Treatment for active nephritis (various agents)	12 weeks	***Source:*** WB<br>***Number of ISG:*** 7 (IFN-induced chemokines)<br>***Main findings:*** changes observed (3/4 had their chemokine-induced score reduced and improved nephritis; 1/4 had their score	High

						increased and progressed to renal failure)	
		Furie, 2019 [79]	12	BIIB059 (20 mg/kg) [BDCA2]	1 day	***Source:*** WB<br>***Number of ISG:*** 9<br>***Main findings:*** changes observed (a single BIIB059 dose lead to a rapid and partial neutralization of ISG expression compared to placebo)	Unclear
		Hasni, 2019 [229]	10	Omalizumab [IgE]	16 weeks	***Source:*** WB<br>***Number of ISG:*** 4<br>***Main findings:*** no change (IFN score: all group p=0.110, IFNhigh p=0.052)	Unclear
		Kawasaki, 2011 [198]	12	Prednisolone initiation or increase in doses	Before (active phase) and after (inactive phase) of steroid treatment	***Source:*** CD3+ T-cells<br>***Number of ISG:*** 6<br>***Main findings:*** changes observed (higher ISG expression in active vs non-active phase, all p<0.050)	Low
		Li, 2010 [39]	4	High dose IV prednisolone (1 g/day)	3 days	***Source:*** WB<br>***Number of ISG:*** 3<br>***Main findings:*** changes observed (Mx1 expression decreased at day 3, p<0.050)	High
		McBride, 2012 [230]	32	Rontalizumab (single-dose or repeat dose) [IFN alpha protein]	6 months	***Source:*** WB<br>***Number of ISG:*** 7<br>***Main findings:*** changes observed (rontalizumab induced a substantial decline (>50%) in relative gene expression at 3 mg/kg (31.3% of baseline) and 10 mg/kg (23.1% of baseline), different doses lead to different length of the sustained suppression)	Unclear
		Merrill, 2011 [231]	34	Sifalimumab (doses from 0 to 30 mg/kg) [IFN alpha protein]	84 days	***Source:*** WB<br>***Number of ISG:*** 21<br>***Main findings:*** changes observed (doses 0, 3 and 10 led to changes in IFN score at day 14 (p<0.050, p=0.010 and p=0.010); no changes	Unclear

						with doses 1 or 3, p=0.090 and p=0.060)	
**Reporter cell assays**	*Reporter cell assays by qPCR*	Hua, 2006 [109]	2	Usual care	5 or 7 months	***Number of ISG:*** 5 ***Main findings:*** changes observed (IFN score varied over time, but fluctuations may be confounded by GC pulses or HCQ)	High
		Niewold, 2008 [111]	28	Usual care	Not specified	***Number of ISG:*** 3 ***Main findings:*** changes observed (patients could be classified into stable or unstable IFN signature)	High
**Cytopathic cell assay**		Lackovic, 1984 [118]	4	Usual care	Not specified	No change (no consistent pattern)	High
		von Wussow, 1988 [232]	61	Usual care	Not specified	No change (no consistent pattern)	High
**Plaque assay**		Ytterberg, 1982 [124]	14	Usual care	Not specified	Changes observed (IFN bioactivity patterns found in association with disease activity, p<0.050)	High

Supplementary Table 19: Summary of the studies reporting assays analyzing responsiveness to change of IFN assays in RA.

Category	Sub-category	Reference	n	Treatment [target]	Timepoints	Results	Risk of bias
**Immunoassays**	*Immunoassays for IFN-induced proteins*	Quartier, 2011 [233]	24	Anakinra [IL-1a]	6 months	***Target:*** 2 chemokines (IP-10 and TRAIL) ***Main findings:*** changes observed (IP-10 and TRAIL significantly increased in anakinra-treated patients at 6 months, p=0.0316 and p=0.0003)	Unclear
**Microarrays**	*Microarray modules*	Quartier, 2011 [233]	24	Anakinra [IL-1a]	6 months	***Source:*** WB ***Main findings:*** changes observed (coordinated upregulation of type I IFN-inducible transcripts (module M3.1) in the anakinra but not in the placebo-treated group, regardless of the clinical response)	Unclear
	Microarray scores and clustering	van Baarsen, 2010 [212]	15	Anti-TNF (infliximab) [TNF protein]	1 month	***Source:*** WB ***Number of ISG:*** 34 ***Main findings:*** no changes (no variation in ISG (mean 34 genes or mean 3 IFN genes) after 1 month upon anti-TNF)	Low
**qPCR**	*qPCR for IFN scores*	Bienkowska, 2014 [234]	340	Baminercept (700 or 200 mg) [lymphotoxin β]	14 weeks	***Source:*** WB ***Number of ISG:*** 15/3 ***Main findings:*** changes observed (IFN reduced in IFNhigh patients upon treatment in two cohorts)	Unclear
		Cantaert, 2010 [204]	18	Anti-TNF [TNF protein]	1 month	***Source:*** WB ***Number of ISG:*** 43 ***Main findings:*** no change (TNF blockade did not modulate the mean expression of ISG, increasing in 3 patients, decrease in 11 patients)	Low
		Cooles, 2018 [138]	11	Initiation of GC, MTX, HCQ	6 months	***Source:*** WB ***Number of ISG:*** 5 ***Main findings:*** changes observed (sustained decrease in IGS after 6 months of treatment, 1 month: p<0.010; 3 months: p<0.010 and 6 months: p<0.050)	Low

		Rodríguez-Carrio, 2017 [137]	13	Anti-TNF (various agents) [TNF protein]	3 months	***Source:*** WB<br>***Number of ISG:*** 4<br>***Main findings:*** no change (no changes in ISG or IFN score upon TNF-blockade, all p>0.050)	Low
		Rodríguez-Carrio, 2019 [84]	13	Anti-TNF (various agents) [TNF protein]	3 months	***Source:*** WB<br>***Number of ISG:*** 5<br>***Main findings:*** no change (no changes in ISG or IFN score upon TNF-blockade regardless of the response status, all p>0.050)	Low
		Vosslamber, 2011 [219]	22	Rituximab [CD20]	3 months	***Source:*** WB<br>***Number of ISG:*** 6<br>***Main findings:*** changes observed (IFN score increased at 3 months compared to baseline in responders but not in non-responders, p=0.0040)	Low
		Weix, 2013 [125]	10	Not specified (pregnant RA patients)	Pre-pregnancy to postpartum	***Source:*** PBMC<br>***Number of ISG:*** 6<br>***Main findings:*** changes observed (IFI35 and IFI44 expression changed along pregnancy and postpartum; no differences in IFI44L, IFIT3 and OAS1)	Unclear
**Reporter cell assays**	*Reporter cell assays by qPCR*	Muskardin, 2015 [222]	32<br>92	Anti-TNF (various agents) [TNF protein]	4-6 weeks	***Number of ISG:*** 5<br>***Main findings:*** changes observed (decrease in total IFN activity, IFNb activity and IFNb/a ratio was associated with no response, all p<0.050)	Unclear

Supplementary Table 20: Summary of the studies reporting assays analyzing responsiveness to change of IFN assays in SSc.

Category	Sub-category	Reference	n	Treatment	Timepoints	Results	Risk of bias
**Immunoassays**	*Immunoassays for IFN-induced proteins*	Liu, 2013 [162]	63	Usual care	Consecutive visits (mean time in study: 3.1±1.2 years)	***Target:*** IP-10 and I-TAC<br>***Main findings:*** no changes observed (IP-10 p=0.977; I-TAC p=0.512; IFN-induced chemokine score p=0.621)	Unclear
		Assassi, 2019 [164]	62	Cyclophosphamide (n=35)<br>Hematopoietic cell transplant (n=27)	6 and 12 months	***Target:*** 15 chemokines<br>***Main findings:*** changes observed (chemokine protein score significantly decreased in the hematopoietic cell transplant arm; no significant change in the cyclophosphamide group)	Unclear
**Microarrays**	*Microarray modules*	Assassi, 2019 [164]	62	Cyclophosphamide (n=35)<br>Hematopoietic cell transplant (n=27)	6 and 12 months	***Source:*** WB<br>***Main findings:*** changes observed (decline in IFN modules (M1.2 and M3.4) post treatment, greater effect in the hematopoietic cell transplant group)	Unclear

Supplementary Table 21: Summary of the studies reporting assays analyzing responsiveness to change of IFN assays in pSS.

Category	Sub-category	Reference	n	Treatment [target]	Timepoints	Results	Risk of bias
**Immunoassays**	*Immunoassays detecting IFNa protein*	Pollard, 2013 [143]	28	Rituximab (1000 mg, day 1 and 15) [CD20]	Pre- and post-treatment (5, 12, 24, 36 and 48 weeks)	Changes observed (IFNa decreased levels after treatment at 5 and 12 weeks, p<0.050)	High
	Immunoassays for IFN-induced proteins	Pollard, 2013 [143]	28	Rituximab (1000 mg, day 1 and 15) [CD20]	Pre- and post-treatment (5, 12, 24, 36 and 48 weeks)	***Target:*** MIP-1b and MIG<br>***Main findings:*** changes observed (MIP1b decreased levels after treatment at 12 weeks, p<0.050; MIG decreased levels after treatment at 5, 12 and 36 weeks, all p<0.050)	High
**Flow cytometry**		Rose, 2016 [149]	11	GC (n=5) HCQ (n=6)	Pre- and post-treatment (GC: 5.5 months, HCQ: 5 months)	***Target (source):*** Siglec1 (monocytes)<br>***Main findings:*** changes observed (Siglec1 expression reduced upon GC and HCQ, p=0.028 and p=0.046)	Unclear
**qPCR**	*qPCR for IFN scores*	Brkic, 2013 [154]	69	Usual care	Two measurements (3.6±2.5 years)	***Source:*** sorted cell populations (monocytes)<br>***Number of ISG:*** 5<br>***Main findings:*** no change	High
		Bodewes, 2019 [153]	77	HCQ	Pre- and post-treatment (24 weeks)	***Source:*** WB<br>***Number of ISG:*** 5<br>***Main findings:*** changes observed (IFN-I score reduced upon HCQ treatment at 24 weeks, p<0.050)	Unclear
**Reporter cell assays**	*Reporter cell assays by qPCR*	Mavragani, 2007 [158]	10	Etanercept (25 mg, twice weekly) [TNF protein]	Pre- and post-treatment (12 weeks)	***Number of ISG:*** 2<br>***Main findings:*** changes observed (IFN bioactivity increased upon treatment, baseline 4.12±1.77 vs post-treatment 7.46±5.34, p=0.040)	Unclear

Supplementary Table 22: Summary of the studies reporting assays analyzing responsiveness to change of IFN assays in PM/DM.

Category	Sub-category	Reference	n	Treatment [target]	Timepoints	Results	Risk of bias
**Immunoassays**	*Immunoassays for IFN-induced proteins*	Reed, 2015 [224]	177	Rituximab [CD20]	Pre- and post-treatment (16 weeks)	***Target:*** IP-10, I-TAC and MCP-1<br>***Main findings:*** changes observed (IFN chemokine scores fluctuated upon treatment depending on autoantibody profiles)	Unclear
		Reed, 2012 [223]	51	Immunosuppressive regimens (various agents)	Two consecutive visits (interval not specified)	***Target:*** IP-10, I-TAC and MCP-1<br>***Main findings:*** changes observed (IFN chemokine scores correlated with changes in global (r=0.530, p<0.001), muscle (r=0.500, p<0.001) and extra-skeletal VAS (r=0.550, p<0.001); changes in IP-10 correlated changes in global (r=0.530, p<0.001), muscle (r=0.440, p=0.002) and extra-skeletal VAS (r=0.520, p<0.001)	Low
		López de Padilla, 2015 [235]	200	Rituximab [CD20]	Pre- and post-treatment (8 and 16 weeks)	***Target:*** MCP1, IP10, I-TAC<br>***Main findings:*** no changes	Unclear
**Microarrays**	*Microarray scores and clustering*	Higgs, 2014 [236]	39	Sifalimumab [IFN alpha protein]	Pre- and post-treatment (28, 56 and 98 days)	***Source:*** WB<br>***Number of ISG:*** 13<br>***Main findings:*** changes observed (IFN score neutralized (from 53% to 66%) across the three time points vs placebo, p=0.019)	Unclear
**qPCR**	*qPCR for IFN scores*	Greenberg, 2012 [177]	24	Usual care	Over 80 visits (at least 2/patient)	***Source:*** sorted cell populations (monocytes)<br>***Number of ISG:*** 13<br>***Main findings:*** changes observed (21/24 patients exhibited an elevation of the type I IFN score<br>IFN score, IFI44L and RSAD2 expression	Unclear

						changed in parallel to disease activity over time; 3/24 patients experienced no change in disease activity and exhibited no change in IFN score, IFI44L or RSAD2)	
		Huard, 2017 [209]	13	Usual care	2 or more longitudinal visits/patient	***Source:*** WB<br>***Number of ISG:*** 10<br>***Main findings:*** changes observed (IFN score changed from baseline correlated changes in CDASI, r=0.280, p=0.060)	High
		Walsh, 2007 [178]	9	Usual care	2 paired samples (active vs improving disease)	***Source:*** WB<br>***Number of ISG:*** 25<br>***Main findings:*** changes observed (MxA, RSAD2, IFI44L, HERC5, ISG15 and OASL expression decreased with disease improvement; little changes observed in all genes in refractory patients)	High
		O'Connor, 2006 [179]	14	Immunosuppressive therapy (>1 dose of IV methyprednisolone 30 mg/kg, parenteral MTX, folic acid and others)	1 year	***Source:*** WB<br>***Number of ISG:*** 1<br>***Main findings:*** changes observed (change in MxA expression correlated change in DAS muscle (r=0.630, p=0.040) but not DAS skin (r=0.000, p>0.990))	High
		Reed, 2012 [223]	51	Immunosuppressive regimens (various agents)	Two consecutive visits (interval not specified)	***Source:*** WB<br>***Number of ISG:*** 3<br>***Main findings:*** changes observed (changes in IFN gene score correlated changes in global (r=0.330, p=0.023) and muscle VAS (r=0.440, p=0.002)	Low
**Reporter cell assays**	*Reporter cell assays by qPCR*	Dastmalchi, 2008 [237]	10	Infliximab [TNF protein]	Not specified	***Number of ISG:*** 3<br>***Main findings:*** changes observed (IFN bioactivity increased upon treatment, regardless of disease improvement, baseline: 1.54 (2.41) vs post-treatment: 3.88 (4.03), p=0.037)	Low

REFERENCES

1 Whiting PF. QUADAS-2: A Revised Tool for the Quality Assessment of Diagnostic Accuracy Studies. *Ann Intern Med* 2011;**155**:529. doi:10.7326/0003-4819-155-8-201110180-00009

2 Moola S, Munn Z, Tufanaru C, *et al.* Chapter 7: Systematic reviews of etiology and risk. In: Aromataris E, Munn Z, eds. *JBI Manual for Evidence Synthesis*. JBI 2020.

3 Hayden JA, van der Windt DA, Cartwright JL, *et al.* Assessing Bias in Studies of Prognostic Factors. *Ann Intern Med* 2013;**158**:280. doi:10.7326/0003-4819-158-4-201302190-00009

4 Sterne JAC, Savović J, Page MJ, *et al.* RoB 2: a revised tool for assessing risk of bias in randomised trials. *BMJ* 2019;:l4898. doi:10.1136/bmj.l4898

5 Munn Z, Barker TH, Moola S, *et al.* Methodological quality of case series studies: an introduction to the JBI critical appraisal tool. *JBI Evid Synth* 2020;**18**:2127–33. doi:10.11124/JBISRIR-D-19-00099

6 Abdel Galil SM, El-Shafey AM, Abdul-Maksoud RS, *et al.* Interferon alpha gene expression and serum level association with low vitamin D levels in Egyptian female patients with systemic lupus erythematosus. *Lupus* 2018;**27**:199–209. doi:https://dx.doi.org/10.1177/0961203317716321

7 Becker-Merok A, Ostli-Eilersten G, Lester S, *et al.* Circulating interferon-alpha2 levels are increased in the majority of patients with systemic lupus erythematosus and are associated with disease activity and multiple cytokine activation. *Lupus* 2013;**22**:155–63. doi:https://dx.doi.org/10.1177/0961203312468964

8 Fragoso-Loyo H, Atisha-Fregoso Y, Nunez-Alvarez CA, *et al.* Utility of interferon-alpha as a biomarker in central neuropsychiatric involvement in systemic lupus erythematosus. *Journal of Rheumatology* 2012;**39**:504–9. doi:https://dx.doi.org/10.3899/jrheum.110983

9 Gao L, M OC, Allen M, *et al.* Bone marrow mesenchymal stem cells from patients with SLE maintain an interferon signature during in vitro culture. *Cytokine* 2019;**(no pagina**. doi:http://dx.doi.org/10.1016/j.cyto.2019.05.012

10 Mozo L, Lopez P, Caminal-Montero L, *et al.* Anti-ribosomal P antibodies are associated with elevated circulating IFNalpha and IL-10 levels in systemic lupus erythematosus patients. *Lupus* 2014;**23**:1477–85. doi:https://dx.doi.org/10.1177/0961203314546020

11 Oke V, Brauner S, Larsson A, *et al.* IFN-lambda1 with Th17 axis cytokines and IFN-alpha define different subsets in systemic lupus erythematosus (SLE). *Arthritis Res Ther* 2017;**19**:139. doi:https://dx.doi.org/10.1186/s13075-017-1344-7

12 Postal M, Sinicato NA, Pelicari KO, *et al.* Clinical and serological manifestations associated with interferon-alpha levels in childhood-onset systemic lupus erythematosus. *Clinics (Sao Paulo)* 2012;**67**:157–62.

13 Shahin D, El-Refaey AM, El-Hawary AK, *et al.* Serum interferon-alpha level in first degree relatives of systemic lupus erythematosus patients: Correlation with autoantibodies titers. *Egyptian Journal of Medical Human Genetics* 2011;**12**:139–46. doi:http://dx.doi.org/10.1016/j.ejmhg.2011.06.009

14 Willis R, Seif AM, McGwin Jr. G, *et al.* Effect of hydroxychloroquine treatment on pro-inflammatory cytokines and disease activity in SLE patients: data from LUMINA (LXXV), a multiethnic US cohort. *Lupus* 2012;**21**:830–5. doi:https://dx.doi.org/10.1177/0961203312437270

15 Ye H, Wang X, Wang L, *et al.* Full high-throughput sequencing analysis of differences in expression profiles of long noncoding RNAs and their mechanisms of action in systemic lupus erythematosus. *Arthritis Res Ther* 2019;**21 (1) (no**. doi:http://dx.doi.org/10.1186/s13075-019-1853-7

16 Yin ZH, Huang JX, He WZ, *et al.* Serum level of eight cytokines in Han Chinese patients with systemic lupus erythematosus using multiplex fluorescent microsphere method. *Central European Journal of Immunology* 2014;**39**:228–35. doi:10.5114/ceji.2014.43728

17 Zecevic L, Karamehic J, Coric J, *et al.* Potential Immune Biomarkers in Diagnosis and Clinical Management for Systemic Lupus Erythematosus. *J Med Biochem* 2018;**37**:163–71. doi:http://dx.doi.org/10.1515/jomb-2017-0048

18 Zhuang H, Narain S, Sobel E, *et al.* Association of anti-nucleoprotein autoantibodies with upregulation of Type I interferon-inducible gene transcripts and dendritic cell maturation in systemic lupus erythematosus. *Clinical Immunology* 2005;**117**:238–50.

19 Fernández Matilla M, Grau García E, Fernández-Llanio Comella N, *et al.* Increased interferon-1α, interleukin-10 and BLyS concentrations as clinical activity biomarkers in systemic lupus erythematosus. *Medicina Clínica (English Edition)* 2019;**153**:225–31. doi:10.1016/j.medcle.2019.07.003

20 Lood C, Amisten S, Gullstrand B, *et al.* Platelet transcriptional profile and protein expression in patients with systemic lupus erythematosus: up-regulation of the type I interferon system is strongly associated with vascular disease. *Blood* 2010;**116**:1951–7. doi:10.1182/blood-2010-03-274605

21 Hashad D, Abdelmagid M, Elsherif S. microRNA146a Expression in Lupus Patients With and Without Renal Complications. *J Clin Lab Anal* 2012;**26**:35–40. doi:10.1002/jcla.20501

22 Kanakoudi-Tsakalidou F, Farmaki E, Tzimouli V, *et al.* Simultaneous changes in serum HMGB1 and IFN-α levels and in LAIR-1 expression on plasmatoid dendritic cells of patients with juvenile SLE.. New therapeutic options? *Lupus* 2014;**23**:305–12. doi:10.1177/0961203313519157

23 Ma C, Jiao Y, Zhang J, *et al.* Elevated plasma level of HMGB1 is associated with disease activity and combined alterations with IFN-alpha and TNF-alpha in systemic lupus erythematosus. *Rheumatol Int* 2012;**32**:395–402. doi:10.1007/s00296-010-1636-6

24 Mandal M, Tripathy R, Panda AK, *et al.* Vitamin D levels in Indian systemic lupus erythematosus patients: association with disease activity index and interferon

alpha. *Arthritis Res Ther* 2014;**16**:R49. doi:10.1186/ar4479

25 Pacheco Y, Barahona-Correa J, Monsalve DM, *et al.* Cytokine and autoantibody clusters interaction in systemic lupus erythematosus. *J Transl Med* 2017;**15**:239. doi:10.1186/s12967-017-1345-y

26 Robak E, Smolewski P, Wozniacka A, *et al.* Relationship between peripheral blood dendritic cells and cytokines involved in the pathogenesis of systemic lupus erythematosus. *Eur Cytokine Netw*;**15**:222–30.

27 Nielsen CT, Lood C, Ostergaard O, *et al.* Plasma levels of galectin-3-binding protein reflect type i interferon activity and are increased in patients with systemic lupus erythematosus. *Lupus Sci Med* 2014;**1 (1) (no**. doi:http://dx.doi.org/10.1136/lupus-2014-000026

28 Oliveira JJ, Karrar S, Rainbow DB, *et al.* The plasma biomarker soluble SIGLEC-1 is associated with the type I interferon transcriptional signature, ethnic background and renal disease in systemic lupus erythematosus. *Arthritis Res Ther* 2018;**20**:152. doi:https://dx.doi.org/10.1186/s13075-018-1649-1

29 Olsen NJ, McAloose C, Carter J, *et al.* Clinical and Immunologic Profiles in Incomplete Lupus Erythematosus and Improvement with Hydroxychloroquine Treatment. *Autoimmune Dis* 2016;**2016 (no p**. doi:http://dx.doi.org/10.1155/2016/8791629

30 Van Den Hoogen LL, Van Der Heijden EHM, Hillen MR, *et al.* Galectin-9 reflects the interferon signature and correlates with disease activity in systemic autoimmune diseases. *Ann Rheum Dis* Published Online First: 2018. doi:http://dx.doi.org/10.1136/annrheumdis-2018-214651

31 Wahadat MJ, Bodewes ILA, Maria NI, *et al.* Type I IFN signature in childhood-onset systemic lupus erythematosus: a conspiracy of DNA- and RNA-sensing receptors? *Arthritis Res Ther* 2018;**20**:4. doi:https://dx.doi.org/10.1186/s13075-017-1501-z

32 al-Masri AN, Werfel T, Jakschies D, *et al.* Intracellular staining of Mx proteins in cells from peripheral blood, bone marrow and skin. *Molecular Pathology* 1997;**50**:9–14. doi:10.1136/mp.50.1.9

33 Wirestam L, Enocsson H, Skogh T, *et al.* Interferon-α coincides with suppressed levels of pentraxin-3 (PTX3) in systemic lupus erythematosus and regulates leucocyte PTX3 in vitro. *Clin Exp Immunol* 2017;**189**:83–91. doi:10.1111/cei.12957

34 Blomberg S, Eloranta ML, Cederblad B, *et al.* Presence of cutaneous interferon-a producing cells in patients with systemic lupus erythematosus. *Lupus* 2001;**10**:484–90. doi:10.1191/096120301678416042

35 Mathian A, Mouries-Martin S, Dorgham K, *et al.* Ultrasensitive serum interferon-alpha quantification during SLE remission identifies patients at risk for relapse. *Ann Rheum Dis* 2019;**30**:30. doi:https://dx.doi.org/10.1136/annrheumdis-2019-215571

36 Rodero MP, Decalf J, Bondet V, *et al.* Detection of interferon alpha protein reveals differential levels and cellular sources in disease. *Journal of*

Experimental Medicine 2017;**214**:1547–55. doi:10.1084/jem.20161451

37 SHOU-NEE S, FANG FS, YUMEI W, *et al.* Serum interferon in systemic lupus erythematosus. *British Journal of Dermatology* 1987;**117**:155–9. doi:10.1111/j.1365-2133.1987.tb04111.x

38 Kim T, Kanayama Y, Negoro N, *et al.* Serum levels of interferons in patients with systemic lupus erythematosus. *Clin Exp Immunol* 1987;**70**:562–9. doi:2449306

39 Li Y, Lee PY, Kellner ES, *et al.* Monocyte surface expression of Fcgamma receptor RI (CD64), a biomarker reflecting type-I interferon levels in systemic lupus erythematosus. *Arthritis Res Ther* 2010;**12**:R90. doi:https://dx.doi.org/10.1186/ar3017

40 Wilhelm TR, Taddeo A, Winter O, *et al.* Siglec-1-positive plasmacytoid dendritic cells (pDCs) in human peripheral blood: A semi-mature and myeloid-like subset imbalanced during protective and autoimmune responses. *Clinical Immunology* 2016;**163**:42–51. doi:https://dx.doi.org/10.1016/j.clim.2015.12.001

41 Biesen R, Demir C, Barkhudarova F, *et al.* Sialic acid-binding Ig-like lectin 1 expression in inflammatory and resident monocytes is a potential biomarker for monitoring disease activity and success of therapy in systemic lupus erythematosus. *Arthritis Rheum* 2008;**58**:1136–45. doi:10.1002/art.23404

42 Banchereau R, Hong S, Cantarel B, *et al.* Personalized Immunomonitoring Uncovers Molecular Networks that Stratify Lupus Patients. *Cell* 2016;**165**:551–65. doi:http://dx.doi.org/10.1016/j.cell.2016.03.008

43 Flint SM, Jovanovic V, Teo BW, *et al.* Leucocyte subset-specific type 1 interferon signatures in SLE and other immune-mediated diseases. *RMD Open* 2016;**2**. doi:10.1136/rmdopen-2015-000183

44 Mackay M, Oswald M, Sanchez-Guerrero J, *et al.* Molecular signatures in systemic lupus erythematosus: Distinction between disease flare and infection. *Lupus Sci Med* 2016;**3 (1) (no**. doi:http://dx.doi.org/10.1136/lupus-2016-000159

45 Olferiev M, Jacek E, Kirou KA, *et al.* Novel molecular signatures in mononuclear cell populations from patients with systemic lupus erythematosus. *Clinical Immunology* 2016;**172**:34–43. doi:10.1016/j.clim.2016.08.018

46 Chiche L, Jourde-Chiche N, Whalen E, *et al.* Modular Transcriptional Repertoire Analyses of Adults With Systemic Lupus Erythematosus Reveal Distinct Type I and Type II Interferon Signatures. *Arthritis & Rheumatology* 2014;**66**:1583–95. doi:10.1002/art.38628

47 Assassi S, Mayes MD, Arnett FC, *et al.* Systemic sclerosis and lupus: Points in an interferon-mediated continuum. *Arthritis Rheum* 2010;**62**:589–98. doi:10.1002/art.27224

48 Baechler EC, Bauer JW, Slattery CA, *et al.* An interferon signature in the peripheral blood of dermatomyositis patients is associated with disease activity. *Molecular Medicine* 2007;**13**:59–68.

49 Baechler EC, Batliwalla FM, Karypis G, *et al.* Interferon-inducible gene expression signature in peripheral blood cells of patients with severe lupus. *Proceedings of the National Academy of Sciences* 2003;**100**:2610–5.

doi:10.1073/pnas.0337679100

50 Becker AM, Dao KH, Han BK, *et al.* SLE peripheral blood B cell, T cell and myeloid cell transcriptomes display unique profiles and each subset contributes to the interferon signature. *PLoS ONE [Electronic Resource]* 2013;**8**:e67003. doi:https://dx.doi.org/10.1371/journal.pone.0067003

51 Dey-Rao R, Sinha AA. Genome-wide transcriptional profiling of chronic cutaneous lupus erythematosus (CCLE) peripheral blood identifies systemic alterations relevant to the skin manifestation. *Genomics* 2015;**105**:90–100. doi:10.1016/j.ygeno.2014.11.004

52 Higgs BW, Liu Z, White B, *et al.* Patients with systemic lupus erythematosus, myositis, rheumatoid arthritis and scleroderma share activation of a common type I interferon pathway. *Ann Rheum Dis* 2011;**70**:2029–36. doi:https://dx.doi.org/10.1136/ard.2011.150326

53 Hoffman RW, Merrill JT, Alarcon-Riquelme MME, *et al.* Gene Expression and Pharmacodynamic Changes in 1,760 Systemic Lupus Erythematosus Patients From Two Phase III Trials of BAFF Blockade With Tabalumab. *Arthritis and Rheumatology* 2017;**69**:643–54. doi:http://dx.doi.org/10.1002/art.39950

54 Kyogoku C, Smiljanovic B, Grun JR, *et al.* Cell-specific type I IFN signatures in autoimmunity and viral infection: what makes the difference? *PLoS ONE [Electronic Resource]* 2013;**8**:e83776. doi:https://dx.doi.org/10.1371/journal.pone.0083776

55 Lugar PL, Love C, Grammer AC, *et al.* Molecular Characterization of Circulating Plasma Cells in Patients with Active Systemic Lupus Erythematosus. *PLoS One* 2012;**7 (9) (no**. doi:http://dx.doi.org/10.1371/journal.pone.0044362

56 Ye S, Pang H, Gu YY, *et al.* Protein interaction for an interferon-inducible systemic lupus associated gene, IFIT1. *Rheumatology* 2003;**42**:1155–63. doi:http://dx.doi.org/10.1093/rheumatology/keg315

57 HIGGS BW, ZHU W, RICHMAN L, *et al.* Identification of activated cytokine pathways in the blood of systemic lupus erythematosus, myositis, rheumatoid arthritis, and scleroderma patients. *Int J Rheum Dis* 2012;**15**:25–35. doi:10.1111/j.1756-185X.2011.01654.x

58 Lauwerys BR, Hachulla E, Spertini F, *et al.* Down-regulation of interferon signature in systemic lupus erythematosus patients by active immunization with interferon α-kinoid. *Arthritis Rheum* 2013;**65**:447–56. doi:10.1002/art.37785

59 Yao Y, Richman L, Higgs BW, *et al.* Neutralization of interferon-α/β-inducible genes and downstream effect in a phase I trial of an anti-interferon-α monoclonal antibody in systemic lupus erythematosus. *Arthritis Rheum* 2009;**60**:1785–96. doi:10.1002/art.24557

60 Perez-Sanchez C, Barbarroja N, Messineo S, *et al.* Gene profiling reveals specific molecular pathways in the pathogenesis of atherosclerosis and cardiovascular disease in antiphospholipid syndrome, systemic lupus erythematosus and antiphospholipid syndrome with lupus. *Ann Rheum Dis* 2015;**74**:1441–9. doi:10.1136/annrheumdis-2013-204600

61 Smiljanovic B, Grün JR, Biesen R, *et al.* The multifaceted balance of TNF-α and type I/II interferon responses in SLE and RA: how monocytes manage the impact of cytokines. *J Mol Med* 2012;**90**:1295–309. doi:10.1007/s00109-012-0907-y

62 Zhu H, Mi W, Luo H, *et al.* Whole-genome transcription and DNA methylation analysis of peripheral blood mononuclear cells identified aberrant gene regulation pathways in systemic lupus erythematosus. *Arthritis Res Ther* 2016;**18**:162. doi:10.1186/s13075-016-1050-x

63 Kennedy WP, Maciuca R, Wolslegel K, *et al.* Association of the interferon signature metric with serological disease manifestations but not global activity scores in multiple cohorts of patients with SLE. *Lupus Sci Med* 2015;**2**:e000080–e000080. doi:10.1136/lupus-2014-000080

64 Komatsuda A, Wakui H, Iwamoto K, *et al.* Up-regulated expression of Toll-like receptors mRNAs in peripheral blood mononuclear cells from patients with systemic lupus erythematosus. *Clin Exp Immunol* 2008;**152**:482–7. doi:10.1111/j.1365-2249.2008.03646.x

65 Li D, Song L, Fan Y, *et al.* Down-regulation of TIPE2 mRNA expression in peripheral blood mononuclear cells from patients with systemic lupus erythematosus. *Clinical Immunology* 2009;**133**:422–7. doi:10.1016/j.clim.2009.08.014

66 Yuan Y, Ma H, Ye Z, *et al.* Interferon-stimulated gene 15 expression in systemic lupus erythematosus. *Z Rheumatol* 2018;**77**:256–62. doi:10.1007/s00393-017-0274-8

67 Blokland SLM, van den Hoogen LL, Leijten EFA, *et al.* Increased expression of Fas on group 2 and 3 innate lymphoid cells is associated with an interferon signature in systemic lupus erythematosus and Sjogren’s syndrome. *Rheumatology* 2019;**58**:1740–5. doi:https://dx.doi.org/10.1093/rheumatology/kez116

68 Braunstein I, Klein R, Okawa J, *et al.* The interferon-regulated gene signature is elevated in subacute cutaneous lupus erythematosus and discoid lupus erythematosus and correlates with the cutaneous lupus area and severity index score. *British Journal of Dermatology* 2012;**166**:971–5. doi:10.1111/j.1365-2133.2012.10825.x

69 Brkic Z, Corneth OB, van Helden-Meeuwsen CG, *et al.* T-helper 17 cell cytokines and interferon type I: partners in crime in systemic lupus erythematosus? *Arthritis Res Ther* 2014;**16**:R62. doi:https://dx.doi.org/10.1186/ar4499

70 de Jong TD, Lübbers J, Turk S, *et al.* The type I interferon signature in leukocyte subsets from peripheral blood of patients with early arthritis: a major contribution by granulocytes. *Arthritis Res Ther* 2016;**18**:165. doi:10.1186/s13075-016-1065-3

71 Dominguez-Gutierrez PR, Ceribelli A, Satoh M, *et al.* Elevated signal transducers and activators of transcription 1 correlates with increased C-C motif chemokine ligand 2 and C-X-C motif chemokine 10 levels in peripheral blood of patients with systemic lupus erythematosus. *Arthritis Res Ther* 2014;**16 (1) (no**. doi:http://dx.doi.org/10.1186/ar4448

72 Dominguez-Gutierrez PR, Ceribelli A, Satoh M, *et al.* Positive Correlation of STAT1 and miR-146a with Anemia in Patients with Systemic Lupus Erythematosus. *J Clin Immunol* 2014;**34**:171–80. doi:10.1007/s10875-013-9973-3

73 Ekholm L, Kahlenberg JM, Barbasso Helmers S, *et al.* Dysfunction of endothelial progenitor cells is associated with the type I IFN pathway in patients with polymyositis and dermatomyositis. *Rheumatology* 2016;**55**:1987–92.

74 El-Sherbiny YM, Psarras A, Md Yusof MY, *et al.* A novel two-score system for interferon status segregates autoimmune diseases and correlates with clinical features. *Sci Rep* 2018;**8**:5793. doi:10.1038/s41598-018-24198-1

75 Feng X, Chen W, Xiao L, *et al.* Artesunate inhibits type I interferon-induced production of macrophage migration inhibitory factor in patients with systemic lupus erythematosus. *Lupus* 2017;**26**:62–72.

76 Feng X, Huang J, Liu Y, *et al.* Identification of interferon-inducible genes as diagnostic biomarker for systemic lupus erythematosus. *Clin Rheumatol* 2015;**34**:71–9. doi:http://dx.doi.org/10.1007/s10067-014-2799-4

77 Fu Q, Chen X, Cui H, *et al.* Association of elevated transcript levels of interferon-inducible chemokines with disease activity and organ damage in systemic lupus erythematosus patients. *Arthritis Res Ther* 2008;**10**:R112. doi:10.1186/ar2510

78 Furie R, Werth VP, Merola JF, *et al.* Monoclonal antibody targeting BDCA2 ameliorates skin lesions in systemic lupus erythematosus. *Journal of Clinical Investigation* 2019;**129**:1359–71. doi:http://dx.doi.org/10.1172/JCI124466

79 Jin Z, Fan W, Jensen MA, *et al.* Single-cell gene expression patterns in lupus monocytes independently indicate disease activity, interferon and therapy. *Lupus Sci Med* 2017;**4 (1) (no**. doi:http://dx.doi.org/10.1136/lupus-2016-000202

80 Landolt-Marticorena C, Bonventi G, Lubovich A, *et al.* Lack of association between the interferon-alpha signature and longitudinal changes in disease activity in systemic lupus erythematosus. *Ann Rheum Dis* 2009;**68**:1440–6. doi:https://dx.doi.org/10.1136/ard.2008.093146

81 Liu M, Liu J, Hao S, *et al.* Higher activation of the interferon-gamma signaling pathway in systemic lupus erythematosus patients with a high type I IFN score: relation to disease activity. *Clin Rheumatol* 2018;**37**:2675–84. doi:https://dx.doi.org/10.1007/s10067-018-4138-7

82 Md Yusof MY, Psarras A, El-Sherbiny YM, *et al.* Prediction of autoimmune connective tissue disease in an at-risk cohort: prognostic value of a novel two-score system for interferon status. *Ann Rheum Dis* 2018;**77**:1432–9. doi:10.1136/annrheumdis-2018-213386

83 Reynolds JA, Briggs TA, Rice GI, *et al.* Type i interferon in patients with systemic autoimmune rheumatic disease is associated with haematological abnormalities and specific autoantibody profiles. *Arthritis Res Ther* 2019;**21 (1) (no**. doi:http://dx.doi.org/10.1186/s13075-019-1929-4

84 Rodriguez-Carrio J, Lopez P, Alperi-Lopez M, *et al.* IRF4 and IRG Delineate Clinically Relevant Gene Expression Signatures in Systemic Lupus

Erythematosus end Rheumatoid Arthritis. *Front Immunol* 2019;**9**. doi:10.3389/fimmu.2018.03085

85 Tang J, Gu Y, Zhang M, *et al.* Increased expression of the type I interferon-inducible gene, lymphocyte antigen 6 complex locus E, in peripheral blood cells is predictive of lupus activity in a large cohort of Chinese lupus patients. *Lupus* 2008;**17**:805–13. doi:https://dx.doi.org/10.1177/0961203308089694

86 Tyden H, Lood C, Gullstrand B, *et al.* Endothelial dysfunction is associated with activation of the type i interferon system and platelets in patients with systemic lupus erythematosus. *RMD Open* 2017;**3 (2) (no**. doi:http://dx.doi.org/10.1136/rmdopen-2017-000508

87 van den Hoogen LL, van Roon JAG, Mertens JS, *et al.* Galectin-9 is an easy to measure biomarker for the interferon signature in systemic lupus erythematosus and antiphospholipid syndrome. *Ann Rheum Dis* 2018;**77**:1810–4. doi:10.1136/annrheumdis-2018-213497

88 van den Hoogen LL, Fritsch-Stork RDE, Versnel MA, *et al.* Monocyte type I interferon signature in antiphospholipid syndrome is related to proinflammatory monocyte subsets, hydroxychloroquine and statin use. *Ann Rheum Dis* 2016;**75**:e81–e81. doi:10.1136/annrheumdis-2016-210485

89 Bodewes ILA, Huijser E, van Helden-Meeuwsen CG, *et al.* TBK1: A key regulator and potential treatment target for interferon positive Sjogren's syndrome, systemic lupus erythematosus and systemic sclerosis. *J Autoimmun* 2018;**91**:97–102. doi:https://dx.doi.org/10.1016/j.jaut.2018.02.001

90 Casey KA, Guo X, Smith MA, *et al.* Type I interferon receptor blockade with anifrolumab corrects innate and adaptive immune perturbations of SLE. *Lupus Sci Med* 2018;**5**:e000286. doi:10.1136/lupus-2018-000286

91 Dominguez-Gutierrez PR, Ceribelli A, Satoh M, *et al.* Reduced levels of CCL2 and CXCL10 in systemic lupus erythematosus patients under treatment with prednisone, mycophenolate mofetil, or hydroxychloroquine, except in a high STAT1 subset. *Arthritis Res Ther* 2014;**16**:R23. doi:10.1186/ar4451

92 Feng X, Wu H, Grossman JM, *et al.* Association of increased interferon-inducible gene expression with disease activity and lupus nephritis in patients with systemic lupus erythematosus. *Arthritis Rheum* 2006;**54**:2951–62. doi:10.1002/art.22044

93 Kirou KA, Lee C, George S, *et al.* Activation of the interferon-α pathway identifies a subgroup of systemic lupus erythematosus patients with distinct serologic features and active disease. *Arthritis Rheum* 2005;**52**:1491–503. doi:10.1002/art.21031

94 Lee PY, Li Y, Richards HB, *et al.* Type I interferon as a novel risk factor for endothelial progenitor cell depletion and endothelial dysfunction in systemic lupus erythematosus. *Arthritis Rheum* 2007;**56**:3759–69. doi:10.1002/art.23035

95 Li Q-Z, Zhou J, Lian Y, *et al.* Interferon signature gene expression is correlated with autoantibody profiles in patients with incomplete lupus syndromes. *Clin Exp Immunol* 2010;**159**:281–91. doi:10.1111/j.1365-2249.2009.04057.x

96 Kellner ES, Lee PY, Li Y, *et al.* Endogenous type-I interferon activity is not associated with depression or fatigue in systemic lupus erythematosus. *J Neuroimmunol* 2010;**223**:13–9. doi:10.1016/j.jneuroim.2010.03.018

97 Wither J, Johnson SR, Liu T, *et al.* Presence of an interferon signature in individuals who are anti-nuclear antibody positive lacking a systemic autoimmune rheumatic disease diagnosis. *Arthritis Res Ther* 2017;**19**. doi:10.1186/s13075-017-1243-y

98 Coit P, Jeffries M, Altorok N, *et al.* Genome-wide DNA methylation study suggests epigenetic accessibility and transcriptional poising of interferon-regulated genes in naive CD4+ T cells from lupus patients. *J Autoimmun* 2013;**43**:78–84. doi:https://dx.doi.org/10.1016/j.jaut.2013.04.003

99 Joseph S, George NI, Green-Knox B, *et al.* Epigenome-wide association study of peripheral blood mononuclear cells in systemic lupus erythematosus: Identifying DNA methylation signatures associated with interferon-related genes based on ethnicity and SLEDAI. *J Autoimmun* 2019;**96**:147–57. doi:http://dx.doi.org/10.1016/j.jaut.2018.09.007

100 Yeung KS, Chung BH, Choufani S, *et al.* Genome-Wide DNA Methylation Analysis of Chinese Patients with Systemic Lupus Erythematosus Identified Hypomethylation in Genes Related to the Type I Interferon Pathway. *PLoS ONE [Electronic Resource]* 2017;**12**:e0169553. doi:https://dx.doi.org/10.1371/journal.pone.0169553

101 Absher DM, Li X, Waite LL, *et al.* Genome-Wide DNA Methylation Analysis of Systemic Lupus Erythematosus Reveals Persistent Hypomethylation of Interferon Genes and Compositional Changes to CD4+ T-cell Populations. *PLoS Genet* 2013;**9**:e1003678. doi:10.1371/journal.pgen.1003678

102 Coit P, Yalavarthi S, Ognenovski M, *et al.* Epigenome profiling reveals significant DNA demethylation of interferon signature genes in lupus neutrophils. *J Autoimmun* 2015;**58**:59–66. doi:10.1016/j.jaut.2015.01.004

103 Imgenberg-Kreuz J, Almlöf JC, Leonard D, *et al.* Shared and Unique Patterns of DNA Methylation in Systemic Lupus Erythematosus and Primary Sjögren's Syndrome. *Front Immunol* 2019;**10**. doi:10.3389/fimmu.2019.01686

104 Ulff-Møller CJ, Asmar F, Liu Y, *et al.* Twin DNA Methylation Profiling Reveals Flare-Dependent Interferon Signature and B Cell Promoter Hypermethylation in Systemic Lupus Erythematosus. *Arthritis & Rheumatology* 2018;**70**:878–90. doi:10.1002/art.40422

105 Weeding E, Coit P, Yalavarthi S, *et al.* Genome-wide DNA methylation analysis in primary antiphospholipid syndrome neutrophils. *Clinical Immunology* 2018;**196**:110–6. doi:10.1016/j.clim.2018.11.011

106 Zhu H, Mi W, Luo H, *et al.* Whole-genome transcription and DNA methylation analysis of peripheral blood mononuclear cells identified aberrant gene regulation pathways in systemic lupus erythematosus. *Arthritis Res Ther* 2016;**18**:162. doi:10.1186/s13075-016-1050-x

107 Zhao M, Zhou Y, Zhu B, *et al.* IFI44L promoter methylation as a blood biomarker for systemic lupus erythematosus. *Ann Rheum Dis* 2016;**75**:1998–2006.

doi:10.1136/annrheumdis-2015-208410

108 Andrade D, Kim M, Blanco LP, *et al.* Interferon-α and Angiogenic Dysregulation in Pregnant Lupus Patients Who Develop Preeclampsia. *Arthritis & Rheumatology* 2015;**67**:977–87. doi:10.1002/art.39029

109 Hua J, Kirou K, Lee C, *et al.* Functional assay of type I interferon in systemic lupus erythematosus plasma and association with anti–RNA binding protein autoantibodies. *Arthritis Rheum* 2006;**54**:1906–16. doi:10.1002/art.21890

110 Somers EC, Zhao W, Lewis EE, *et al.* Type I interferons are associated with subclinical markers of cardiovascular disease in a cohort of systemic lupus erythematosus patients. *PLoS ONE [Electronic Resource]* 2012;**7**:e37000. doi:https://dx.doi.org/10.1371/journal.pone.0037000

111 Niewold TB, Rivera TL, Buyon JP, *et al.* Serum type I interferon activity is dependent on maternal diagnosis in anti-SSA/Ro–positive mothers of children with neonatal lupus. *Arthritis Rheum* 2008;**58**:541–6. doi:10.1002/art.23191

112 Weckerle CE, Mangale D, Franek BS, *et al.* Brief Report: Large-scale analysis of tumor necrosis factor α levels in systemic lupus erythematosus. *Arthritis Rheum* 2012;**64**:2947–52. doi:10.1002/art.34483

113 Niewold TB, Hua J, Lehman TJA, *et al.* High serum IFN-α activity is a heritable risk factor for systemic lupus erythematosus. *Genes Immun* 2007;**8**:492–502. doi:10.1038/sj.gene.6364408

114 Kirou KA, Lee C, George S, *et al.* Coordinate overexpression of interferon-?-induced genes in systemic lupus erythematosus. *Arthritis Rheum* 2004;**50**:3958–67. doi:10.1002/art.20798

115 Biswas PS, Pawaria S, Maers K, *et al.* Complement component C5a permits the co-existence of pathogenic Th17 cells and type I interferon in lupus. *Cytokine* 2013;**63 (3)**:248. doi:http://dx.doi.org/10.1016/j.cyto.2013.06.026

116 Dall'era MC, Cardarelli PM, Preston BT, *et al.* Type I interferon correlates with serological and clinical manifestations of SLE. *Ann Rheum Dis* 2005;**64**:1692–7.

117 Kato Y, Park J, Takamatsu H, *et al.* Apoptosis-derived membrane vesicles drive the cGAS-STING pathway and enhance type I IFN production in systemic lupus erythematosus. *Ann Rheum Dis* 2018;**77**:1507–15. doi:https://dx.doi.org/10.1136/annrheumdis-2018-212988

118 Lackovic V, Borecky L, Rovensky J, *et al.* Periodicity of interferon appearance in serum of patients with systemic lupus erythematosus. *Arthritis Rheum* 1984;**27**:597–8.

119 Preble O, Black R, Friedman R, *et al.* Systemic lupus erythematosus: presence in human serum of an unusual acid-labile leukocyte interferon. *Science (1979)* 1982;**216**:429–31. doi:10.1126/science.6176024

120 Hervier B, Beziat V, Haroche J, *et al.* Phenotype and function of natural killer cells in systemic lupus erythematosus: Excess interferon-γ production in patients with active disease. *Arthritis Rheum* 2011;**63**:1698–706. doi:10.1002/art.30313

121 Hooks JJ, Moutsopoulos HM, Geis SA, *et al.* Immune interferon in the circulation

of patients with autoimmune disease. *New England Journal of Medicine* 1979;**301**:5–8.

122 Cesario TC, Andrews BS, Martin DA, *et al.* Interferon in synovial fluid and serum of patients with rheumatic disease. *J Rheumatol* 1983;**10**:647–50.

123 Hooks JJ, Jordan GW, Cupps T, *et al.* Multiple interferons in the circulation of patients with systemic lupus erythematosus and vasculitis. *Arthritis Rheum* 1982;**25**:396–400. doi:10.1002/art.1780250406

124 Ytterberg SR, Schnitzer TJ. Serum interferon levels in patients with systemic lupus erythematosus. *Arthritis Rheum* 1982;**25**:401–6. doi:10.1002/art.1780250407

125 Weix J, Haupl T, Raio L, *et al.* The physiologic increase in expression of some type I IFN-inducible genes during pregnancy is not associated with improved disease activity in pregnant patients with rheumatoid arthritis. *Transl Res* 2013;**161**:505–12. doi:https://dx.doi.org/10.1016/j.trsl.2013.02.007

126 Rodriguez-Carrio J, de Paz B, Lopez P, *et al.* IFNalpha serum levels are associated with endothelial progenitor cells imbalance and disease features in rheumatoid arthritis patients. *PLoS ONE [Electronic Resource]* 2014;**9**:e86069. doi:https://dx.doi.org/10.1371/journal.pone.0086069

127 Lepore L, Pennesi M, Saletta S, *et al.* Study of IL-2, IL-6, TNF alpha, IFN gamma and beta in the serum and synovial fluid of patients with juvenile chronic arthritis. *Clin Exp Rheumatol*;**12**:561–5. doi:7531125

128 Båve U, Nordmark G, Lövgren T, *et al.* Activation of the type I interferon system in primary Sjögren's syndrome: A possible etiopathogenic mechanism. *Arthritis Rheum* 2005;**52**:1185–95. doi:10.1002/art.20998

129 Fong KY, Boey ML, Koh WH, *et al.* Cytokine concentrations in the synovial fluid and plasma of rheumatoid arthritis patients: correlation with bony erosions. *Clin Exp Rheumatol*;**12**:55–8. doi:8162643

130 Shiozawa S, Chihara K, Shiozawa K. A sensitive radioimmunoassay for alpha-interferon: Circulating alpha-interferon-like substance in the plasma of health individuals and rheumatoid arthritis patients. *Clin Exp Immunol* 1986;**66**:77–87.

131 Bennett L, Palucka AK, Arce E, *et al.* Interferon and Granulopoiesis Signatures in Systemic Lupus Erythematosus Blood. *J Exp Med* 2003;**197**:711–23. doi:10.1084/jem.20021553

132 Reynier F, Petit F, Paye M, *et al.* Importance of correlation between gene expression levels: application to the type I interferon signature in rheumatoid arthritis. *PLoS ONE [Electronic Resource]* 2011;**6**:e24828. doi:https://dx.doi.org/10.1371/journal.pone.0024828

133 van der Pouw Kraan TC, Wijbrandts CA, van Baarsen LG, *et al.* Rheumatoid arthritis subtypes identified by genomic profiling of peripheral blood cells: assignment of a type I interferon signature in a subpopulation of patients. *Ann Rheum Dis* 2007;**66**:1008–14.http://ovidsp.ovid.com/ovidweb.cgi?T=JS&CSC=Y&NEWS=N&PAGE=fulltext&D=med5&AN=17223656

134 Li Q-Z, Karp DR, Quan J, *et al.* Risk factors for ANA positivity in healthy persons. *Arthritis Res Ther* 2011;**13**:R38. doi:10.1186/ar3271

135 Castaneda-Delgado JE, Bastian-Hernandez Y, Macias-Segura N, *et al.* Type I interferon gene response is increased in early and established rheumatoid arthritis and correlates with autoantibody production. *Front Immunol* 2017;**8 (MAR) (n**. doi:http://dx.doi.org/10.3389/fimmu.2017.00285

136 de Jong TD, Vosslamber S, Mantel E, *et al.* Physiological evidence for diversification of IFNaalpha- and IFNbeta-mediated response programs in different autoimmune diseases. *Arthritis Res Ther* 2016;**18 (1) (no**. doi:http://dx.doi.org/10.1186/s13075-016-0946-9

137 Rodriguez-Carrio J, Alperi-Lopez M, Lopez P, *et al.* Heterogeneity of the Type I Interferon Signature in Rheumatoid Arthritis: A Potential Limitation for Its Use As a Clinical Biomarker. *Front Immunol* 2017;**8**:2007. doi:https://dx.doi.org/10.3389/fimmu.2017.02007

138 Cooles FAH, Anderson AE, Lendrem DW, *et al.* The interferon gene signature is increased in patients with early treatment-naive rheumatoid arthritis and predicts a poorer response to initial therapy. *Journal of Allergy & Clinical Immunology* 2018;**141**:445-448.e4. doi:https://dx.doi.org/10.1016/j.jaci.2017.08.026

139 Wright HL, Thomas HB, Moots RJ, *et al.* Interferon gene expression signature in rheumatoid arthritis neutrophils correlates with a good response to TNFi therapy. *Rheumatology* 2015;**54**:188–93. doi:https://dx.doi.org/10.1093/rheumatology/keu299

140 Mavragani CP, La DT, Stohl W, *et al.* Association of the response to tumor necrosis factor antagonists with plasma type I interferon activity and interferon-β/α ratios in rheumatoid arthritis patients: A post hoc analysis of a predominantly Hispanic cohort. *Arthritis Rheum* 2010;**62**:392–401. doi:10.1002/art.27226

141 Hertzog PJ, Emery P, Cheetham BF, *et al.* Interferons in rheumatoid arthritis: alterations in production and response related to disease activity. *Clin Immunol Immunopathol* 1988;**48**:192–201. doi:10.1016/0090-1229(88)90083-9

142 Arvin AM, Miller 3rd JJ. Acid labile alpha-interferon in sera and synovial fluids from patients with juvenile arthritis. *Arthritis Rheum* 1984;**27**:582–5.

143 Pollard RPE, Abdulahad WH, Bootsma H, *et al.* Predominantly proinflammatory cytokines decrease after B cell depletion therapy in patients with primary Sjogren's syndrome. *Ann Rheum Dis* 2013;**72**:2048–50. doi:http://dx.doi.org/10.1136/annrheumdis-2013-203447

144 Zheng L, Zhang Z, Yu C, *et al.* Association between IFN-alpha and primary Sjogren's syndrome. *Oral Surg Oral Med Oral Pathol Oral Radiol Endod* 2009;**107**:e12-8. doi:https://dx.doi.org/10.1016/j.tripleo.2008.09.015

145 Imgenberg-Kreuz J, Sandling JK, Björk A, *et al.* Transcription profiling of peripheral B cells in antibody-positive primary Sjögren's syndrome reveals upregulated expression of CX3CR1 and a type I and type II interferon signature. *Scand J Immunol* 2018;**87**:e12662. doi:10.1111/sji.12662

146 Wildenberg ME, van Helden-Meeuwsen CG, van de Merwe JP, *et al.* Systemic

increase in type I interferon activity in Sjögren's syndrome: A putative role for plasmacytoid dendritic cells. *Eur J Immunol* 2008;**38**:2024–33. doi:10.1002/eji.200738008

147 Alunno A, Caneparo V, Bistoni O, *et al.* Interferon gamma-inducible protein 16 (IFI16) in rheumatoid arthritis: A novel biomarker for pulmonary involvement? *Ann Rheum Dis* 2015;**2)**:427. doi:http://dx.doi.org/10.1136/annrheumdis-2015-eular.1811

148 Maria NI, Brkic Z, Waris M, *et al.* MxA as a clinically applicable biomarker for identifying systemic interferon type I in primary Sjogren's syndrome. *Ann Rheum Dis* 2014;**73**:1052–9. doi:https://dx.doi.org/10.1136/annrheumdis-2012-202552

149 Rose T, Szelinski F, Lisney A, *et al.* SIGLEC1 is a biomarker of disease activity and indicates extraglandular manifestation in primary Sjogren's syndrome. *RMD Open* 2016;**2**. doi:10.1136/rmdopen-2016-000292

150 SHIOZAWA S, SHIOZAWA K, SHIMIZU S, *et al.* IMMUNOREACTIVE CIRCULATING ALPHA-INTERFERON IS LOW IN SJÖGREN'S SYNDROME. *Rheumatology* 1990;**29**:50–2. doi:10.1093/rheumatology/29.1.50

151 Emamian ES, Leon JM, Lessard CJ, *et al.* Peripheral blood gene expression profiling in Sjogren's syndrome. *Genes Immun* 2009;**10**:285–96. doi:https://dx.doi.org/10.1038/gene.2009.20

152 Kimoto O, Sawada J, Shimoyama K, *et al.* Activation of the interferon pathway in peripheral blood of patients with Sjogren's syndrome. *Journal of Rheumatology* 2011;**38**:310–6. doi:https://dx.doi.org/10.3899/jrheum.100486

153 Bodewes ILA, Gottenberg JE, van Helden-Meeuwsen CG, *et al.* Hydroxychloroquine treatment downregulates systemic interferon activation in primary Sjogren's syndrome in the JOQUER randomized trial. *Rheumatology* 2019;**25**. doi:http://dx.doi.org/10.1093/rheumatology/kez242

154 Brkic Z, Maria NI, van Helden-Meeuwsen CG, *et al.* Prevalence of interferon type I signature in CD14 monocytes of patients with Sjogren's syndrome and association with disease activity and BAFF gene expression. *Ann Rheum Dis* 2013;**72**:728–35. doi:10.1136/annrheumdis-2012-201381

155 Davies R, Sarkar I, Hammenfors D, *et al.* Single cell based phosphorylation profiling identifies alterations in toll-like receptor 7 and 9 signaling in patients with primary sjogren's syndrome. *Front Immunol* 2019;**10 (FEB) (**. doi:http://dx.doi.org/10.3389/fimmu.2019.00281

156 Nezos A, Gravani F, Tassidou A, *et al.* Type I and II interferon signatures in Sjogren's syndrome pathogenesis: Contributions in distinct clinical phenotypes and Sjogren's related lymphomagenesis. *J Autoimmun* 2015;**63**:47–58. doi:https://dx.doi.org/10.1016/j.jaut.2015.07.002

157 Altorok N, Coit P, Hughes T, *et al.* Genome-wide DNA methylation patterns in naive cd4+ t cells from patients with primary sjogren's syndrome. *Arthritis and Rheumatology* 2014;**66**:731–9. doi:http://dx.doi.org/10.1002/art.38264

158 Mavragani CP, Niewold TB, Moutsopoulos NM, *et al.* Augmented interferon-α pathway activation in patients with Sjögren's syndrome treated with etanercept.

Arthritis Rheum 2007;**56**:3995–4004. doi:10.1002/art.23062

159 Eloranta ML, Franck-Larsson K, Lovgren T, *et al.* Type I interferon system activation and association with disease manifestations in systemic sclerosis. *Ann Rheum Dis* 2010;**69**:1396–402. doi:https://dx.doi.org/10.1136/ard.2009.121400

160 Guo X, Higgs BW, Bay-Jensen AC, *et al.* Suppression of T Cell Activation and Collagen Accumulation by an Anti-IFNAR1 mAb, Anifrolumab, in Adult Patients with Systemic Sclerosis. *Journal of Investigative Dermatology* 2015;**135**:2402–9. doi:http://dx.doi.org/10.1038/jid.2015.188

161 Mariotti B, Servaas NH, Rossato M, *et al.* The Long Non-coding RNA NRIR Drives IFN-Response in Monocytes: Implication for Systemic Sclerosis. *Front Immunol* 2019;**10**. doi:10.3389/fimmu.2019.00100

162 Liu X, Mayes MD, Tan FK, *et al.* Correlation of interferon-inducible chemokine plasma levels with disease severity in systemic sclerosis. *Arthritis Rheum* 2013;**65**:226–35. doi:10.1002/art.37742

163 York MR, Nagai T, Mangini AJ, *et al.* A macrophage marker, siglec-1, is increased on circulating monocytes in patients with systemic sclerosis and induced by type i interferons and toll-like receptor agonists. *Arthritis Rheum* 2007;**56**:1010–20. doi:10.1002/art.22382

164 Assassi S, Wang X, Chen G, *et al.* Myeloablation followed by autologous stem cell transplantation normalises systemic sclerosis molecular signatures. *Ann Rheum Dis* 2019;**78**:1371–8. doi:http://dx.doi.org/10.1136/annrheumdis-2019-215770

165 Bos CL, van Baarsen LG, Timmer TC, *et al.* Molecular subtypes of systemic sclerosis in association with anti-centromere antibodies and digital ulcers. *Genes Immun* 2009;**10**:210–8. doi:https://dx.doi.org/10.1038/gene.2008.98

166 Tan FK, Zhou X, Mayes MD, *et al.* Signatures of differentially regulated interferon gene expression and vasculotrophism in the peripheral blood cells of systemic sclerosis patients. *Rheumatology* 2006;**45**:694–702.http://ovidsp.ovid.com/ovidweb.cgi?T=JS&CSC=Y&NEWS=N&PAGE=fulltext&D=med5&AN=16418202

167 Christmann RB, Hayes E, Pendergrass S, *et al.* Interferon and alternative activation of monocyte/macrophages in systemic sclerosis-associated pulmonary arterial hypertension. *Arthritis Rheum* 2011;**63**:1718–28. doi:10.1002/art.30318

168 Airo P, Ghidini C, Zanotti C, *et al.* Upregulation of myxovirus-resistance protein A: a possible marker of type I interferon induction in systemic sclerosis. *Journal of Rheumatology* 2008;**35**:2192–200.http://ovidsp.ovid.com/ovidweb.cgi?T=JS&CSC=Y&NEWS=N&PAGE=fulltext&D=med6&AN=18843779

169 Oliveira DBD, Almeida GMDF, Guedes ACM, *et al.* Basal activation of type i interferons (alpha2 and beta) and 2 ' 5 ' OAS genes: Insights into differential expression profiles of interferon system components in systemic sclerosis. *Int J Rheumatol* 2011;**2011 (no p**. doi:http://dx.doi.org/10.1155/2011/275617

170 Brkic Z, van Bon L, Cossu M, *et al.* The interferon type I signature is present in

systemic sclerosis before overt fibrosis and might contribute to its pathogenesis through high BAFF gene expression and high collagen synthesis. *Ann Rheum Dis* 2016;**75**:1567–73. doi:10.1136/annrheumdis-2015-207392

171 Wang B, Higgs BW, Chang L, *et al.* Pharmacogenomics and Translational Simulations to Bridge Indications for an Anti-Interferon-α Receptor Antibody. *Clin Pharmacol Ther* 2013;**93**:483–92. doi:10.1038/clpt.2013.35

172 Ding W, Pu W, Wang L, *et al.* Genome-Wide DNA Methylation Analysis in Systemic Sclerosis Reveals Hypomethylation of IFN-Associated Genes in CD4+ and CD8+ T Cells. *Journal of Investigative Dermatology* 2018;**138**:1069–77. doi:https://dx.doi.org/10.1016/j.jid.2017.12.003

173 Wuttge DM, Lood C, Tufvesson E, *et al.* Increased serum type I interferon activity in early systemic sclerosis patients is associated with antibodies against Sjogren's syndrome antigens and nuclear ribonucleoprotein antigens. *Scand J Rheumatol* 2013;**42**:235–40. doi:https://dx.doi.org/10.3109/03009742.2012.736532

174 Sun WC, Sun YC, Lin H, *et al.* Dysregulation of the type I interferon system in adult-onset clinically amyopathic dermatomyositis has a potential contribution to the development of interstitial lung disease. *British Journal of Dermatology* 2012;**167**:1236–44. doi:10.1111/j.1365-2133.2012.11145.x

175 Liao AP, Salajegheh M, Nazareno R, *et al.* Interferon beta is associated with type 1 interferon-inducible gene expression in dermatomyositis. *Ann Rheum Dis* 2011;**70**:831–6. doi:https://dx.doi.org/10.1136/ard.2010.139949

176 Horai Y, Koga T, Fujikawa K, *et al.* Serum interferon-alpha is a useful biomarker in patients with anti-melanoma differentiation-associated gene 5 (MDA5) antibody-positive dermatomyositis. *Mod Rheumatol* 2015;**25**:85–9. doi:https://dx.doi.org/10.3109/14397595.2014.900843

177 Greenberg SA, Higgs BW, Morehouse C, *et al.* Relationship between disease activity and type 1 interferon- and other cytokine-inducible gene expression in blood in dermatomyositis and polymyositis. *Genes Immun* 2012;**13**:207–13. doi:https://dx.doi.org/10.1038/gene.2011.61

178 Walsh RJ, Kong SW, Yao Y, *et al.* Type I interferon–inducible gene expression in blood is present and reflects disease activity in dermatomyositis and polymyositis. *Arthritis Rheum* 2007;**56**:3784–92. doi:10.1002/art.22928

179 O'Connor KA, Abbott KA, Sabin B, *et al.* MxA gene expression in juvenile dermatomyositis peripheral blood mononuclear cells: Association with muscle involvement. *Clinical Immunology* 2006;**120**:319–25. doi:10.1016/j.clim.2006.05.011

180 Bilgic H, Ytterberg SR, Amin S, *et al.* Interleukin-6 and type I interferon-regulated genes and chemokines mark disease activity in dermatomyositis. *Arthritis Rheum* 2009;**60**:3436–46. doi:10.1002/art.24936

181 Piper CJM, Li Wilkinson MG, Deakin CT, *et al.* CD19(+)CD24(hi)CD38(hi) B Cells Are Expanded in Juvenile Dermatomyositis and Exhibit a Pro-Inflammatory Phenotype After Activation Through Toll-Like Receptor 7 and Interferon-alpha. *Front Immunol* 2018;**9**. doi:10.3389/fimmu.2018.01372

182 Niewold TB, Kariuki SN, Morgan GA, *et al.* Elevated serum interferon-α activity in juvenile dermatomyositis: Associations with disease activity at diagnosis and after thirty-six months of therapy. *Arthritis Rheum* 2009;**60**:1815–24. doi:10.1002/art.24555

183 Balboni I, Niewold TB, Morgan G, *et al.* Brief Report: Interferon-α Induction and Detection of Anti-Ro, Anti-La, Anti-Sm, and Anti-RNP Autoantibodies by Autoantigen Microarray Analysis in Juvenile Dermatomyositis. *Arthritis Rheum* 2013;**65**:2424–9. doi:10.1002/art.38038

184 Ekholm L, Vosslamber S, Tjärnlund A, *et al.* Autoantibody Specificities and Type I Interferon Pathway Activation in Idiopathic Inflammatory Myopathies. *Scand J Immunol* 2016;**84**:100–9. doi:10.1111/sji.12449

185 Bengtsson AA, Sturfelt G, Truedsson L, *et al.* Activation of type I interferon system in systemic lupus erythematosus correlates with disease activity but not with antiretroviral antibodies. *Lupus* 2000;**9**:664–71.

186 Jonsen A, Bengtsson AA, Nived O, *et al.* The heterogeneity of neuropsychiatric systemic lupus erythematosus is reflected in lack of association with cerebrospinal fluid cytokine profiles. *Lupus* 2003;**12**:846–50.

187 Mathian A, Mouries-Martin S, Dorgham K, *et al.* Monitoring Disease Activity in Systemic Lupus Erythematosus With Single-Molecule Array Digital Enzyme-Linked Immunosorbent Assay Quantification of Serum Interferon-α. *Arthritis & Rheumatology* 2019;**71**:756–65. doi:10.1002/art.40792

188 Rose T, Grutzkau A, Hirseland H, *et al.* IFNalpha and its response proteins, IP-10 and SIGLEC-1, are biomarkers of disease activity in systemic lupus erythematosus. *Ann Rheum Dis* 2013;**72**:1639–45. doi:https://dx.doi.org/10.1136/annrheumdis-2012-201586

189 Rose T, Grutzkau A, Klotsche J, *et al.* Are interferon-related biomarkers advantageous for monitoring disease activity in systemic lupus erythematosus? A longitudinal benchmark study. *Rheumatology* 2017;**56**:1618–26. doi:https://dx.doi.org/10.1093/rheumatology/kex220

190 Schneider L, Colar da Silva AC, Werres Junior LC, *et al.* Vitamin D levels and cytokine profiles in patients with systemic lupus erythematosus. *Lupus* 2015;**24**:1191–7. doi:https://dx.doi.org/10.1177/0961203315584811

191 Oke V, Gunnarsson I, Dorschner J, *et al.* High levels of circulating interferons type I, type II and type III associate with distinct clinical features of active systemic lupus erythematosus. *Arthritis Res Ther* 2019;**21 (1) (no**. doi:http://dx.doi.org/10.1186/s13075-019-1878-y

192 Munroe ME, Vista ES, Merrill JT, *et al.* Pathways of impending disease flare in African-American systemic lupus erythematosus patients. *J Autoimmun* 2017;**78**:70–8. doi:http://dx.doi.org/10.1016/j.jaut.2016.12.005

193 Bauer JW, Baechler EC, Petri M, *et al.* Elevated serum levels of interferon-regulated chemokines are biomarkers for active human systemic lupus erythematosus. *PLoS Medicine / Public Library of Science* 2006;**3**:e491.http://ovidsp.ovid.com/ovidweb.cgi?T=JS&CSC=Y&NEWS=N&PAGE=fulltext&D=med5&AN=17177599

194 Bauer JW, Petri M, Batliwalla FM, *et al.* Interferon-regulated chemokines as biomarkers of systemic lupus erythematosus disease activity: A validation study. *Arthritis Rheum* 2009;**60**:3098–107. doi:10.1002/art.24803

195 Connelly KL, Kandane-Rathnayake R, Hoi A, *et al.* Association of MIF, but not type I interferon-induced chemokines, with increased disease activity in Asian patients with systemic lupus erythematosus. *Sci Rep* 2016;**6**:29909. doi:https://dx.doi.org/10.1038/srep29909

196 Connelly KL, Kandane-Rathnayake R, Huq M, *et al.* Longitudinal association of type 1 interferon-induced chemokines with disease activity in systemic lupus erythematosus. *Sci Rep* 2018;**8**:3268. doi:https://dx.doi.org/10.1038/s41598-018-20203-9

197 Lee JR, Haddon DJ, Wand HE, *et al.* Multiplex giant magnetoresistive biosensor microarrays identify interferon-associated autoantibodies in systemic lupus erythematosus. *Sci Rep* 2016;**6**:27623. doi:https://dx.doi.org/10.1038/srep27623

198 Kawasaki M, Fujishiro M, Yamaguchi A, *et al.* Possible role of the JAK/STAT pathways in the regulation of T cell-interferon related genes in systemic lupus erythematosus. *Lupus* 2011;**20**:1231–9. doi:http://dx.doi.org/10.1177/0961203311409963

199 Petri M, Singh S, Tesfasyone H, *et al.* Longitudinal expression of type I interferon responsive genes in systemic lupus erythematosus. *Lupus* 2009;**18**:980–9. doi:https://dx.doi.org/10.1177/0961203309105529

200 Nikpour M, Dempsey AA, Urowitz MB, *et al.* Association of a gene expression profile from whole blood with disease activity in systemic lupus erythaematosus. *Ann Rheum Dis* 2008;**67**:1069–75.

201 Sharma S, Jin Z, Rosenzweig E, *et al.* Widely divergent transcriptional patterns between SLE patients of different ancestral backgrounds in sorted immune cell populations. *J Autoimmun* 2015;**60**:51–8. doi:https://dx.doi.org/10.1016/j.jaut.2015.04.002

202 Merrill JT, Immermann F, Whitley M, *et al.* The Biomarkers of Lupus Disease Study: A Bold Approach May Mitigate Interference of Background Immunosuppressants in Clinical Trials. *Arthritis & Rheumatology* 2017;**69**:1257–66. doi:10.1002/art.40086

203 Rich SA, Owens TR, Anzola MC, *et al.* Induction of lupus inclusions by sera from patients with systemic lupus erythematosus. *Arthritis Rheum* 1986;**29**:501–7. doi:10.1002/art.1780290407

204 Cantaert T, van Baarsen LG, Wijbrandts CA, *et al.* Type I interferons have no major influence on humoral autoimmunity in rheumatoid arthritis. *Rheumatology* 2010;**49**:156–66. doi:https://dx.doi.org/10.1093/rheumatology/kep345

205 Thurlings RM, Boumans M, Tekstra J, *et al.* Relationship between the type I interferon signature and the response to rituximab in rheumatoid arthritis patients. *Arthritis Rheum* 2010;**62**:3607–14. doi:10.1002/art.27702

206 James JA, Guthridge JM, Chen H, *et al.* Unique Sjogren’s syndrome patient subsets defined by molecular features. *Rheumatology* 2019;**08**.

doi:http://dx.doi.org/10.1093/rheumatology/kez335

207 Olsson P, Bodewes ILA, Nilsson AM, *et al.* Associations of cigarette smoking with disease phenotype and type I interferon expression in primary Sjogren's syndrome. *Rheumatol Int* 2019;**39**:1575–84. doi:http://dx.doi.org/10.1007/s00296-019-04335-3

208 Krol P, Krystufkova O, Polanska M, *et al.* Serum levels of interferon alpha do not correlate with disease activity in patients with dermatomyositis/polymyositis. *Ann Rheum Dis* 2011;**70**:879–80. doi:https://dx.doi.org/10.1136/ard.2010.141051

209 Huard C, Gullà SV, Bennett DV, *et al.* Correlation of cutaneous disease activity with type 1 interferon gene signature and interferon β in dermatomyositis. *British Journal of Dermatology* 2017;**176**:1224–30. doi:10.1111/bjd.15006

210 Bauer JW, Petri M, Batliwalla FM, *et al.* Interferon-regulated chemokines as biomarkers of systemic lupus erythematosus disease activity: A validation study. *Arthritis Rheum* 2009;**60**:3098–107. doi:10.1002/art.24803

211 Steiman AJ, Gladman DD, Ibanez D, *et al.* Lack of Interferon and Proinflammatory Cyto/chemokines in Serologically Active Clinically Quiescent Systemic Lupus Erythematosus. *Journal of Rheumatology* 2015;**42**:2318–26. doi:https://dx.doi.org/10.3899/jrheum.150040

212 van Baarsen LG, Wijbrandts CA, Rustenburg F, *et al.* Regulation of IFN response gene activity during infliximab treatment in rheumatoid arthritis is associated with clinical response to treatment. *Arthritis Res Ther* 2010;**12**:R11. doi:10.1186/ar2912

213 Lubbers J, Brink M, van de Stadt LA, *et al.* The type I IFN signature as a biomarker of preclinical rheumatoid arthritis. *Ann Rheum Dis* 2013;**72**:776–80. doi:https://dx.doi.org/10.1136/annrheumdis-2012-202753

214 Furie R, Khamashta M, Merrill JT, *et al.* Anifrolumab, an Anti-Interferon-α Receptor Monoclonal Antibody, in Moderate-to-Severe Systemic Lupus Erythematosus. *Arthritis & Rheumatology* 2017;**69**:376–86. doi:10.1002/art.39962

215 Kalunian KC, Merrill JT, Maciuca R, *et al.* A Phase II study of the efficacy and safety of rontalizumab (rhuMAb interferon-alpha) in patients with systemic lupus erythematosus (ROSE). *Ann Rheum Dis* 2016;**75**:196–202. doi:https://dx.doi.org/10.1136/annrheumdis-2014-206090

216 Merrill JT, Furie R, Werth VP, *et al.* Anifrolumab effects on rash and arthritis: impact of the type I interferon gene signature in the phase IIb MUSE study in patients with systemic lupus erythematosus. *Lupus Sci Med* 2018;**5**:e000284. doi:https://dx.doi.org/10.1136/lupus-2018-000284

217 Petri M, Wallace DJ, Spindler A, *et al.* Sifalimumab, a Human Anti–Interferon-α Monoclonal Antibody, in Systemic Lupus Erythematosus: A Phase I Randomized, Controlled, Dose-Escalation Study. *Arthritis Rheum* 2013;**65**:1011–21. doi:10.1002/art.37824

218 Sanayama Y, Ikeda K, Saito Y, *et al.* Prediction of Therapeutic Responses to Tocilizumab in Patients With Rheumatoid Arthritis: Biomarkers Identified by

Analysis of Gene Expression in Peripheral Blood Mononuclear Cells Using Genome-Wide DNA Microarray. *Arthritis & Rheumatology* 2014;**66**:1421–31. doi:10.1002/art.38400

219 Vosslamber S, Raterman HG, van der Pouw Kraan TC, *et al.* Pharmacological induction of interferon type I activity following treatment with rituximab determines clinical response in rheumatoid arthritis. *Ann Rheum Dis* 2011;**70**:1153–9. doi:https://dx.doi.org/10.1136/ard.2010.147199

220 Raterman HG, Vosslamber S, de Ridder S, *et al.* The interferon type I signature towards prediction of non-response to rituximab in rheumatoid arthritis patients. *Arthritis Res Ther* 2012;**14**:R95. doi:https://dx.doi.org/10.1186/ar3819

221 de Jong TD, Vosslamber S, Blits M, *et al.* Effect of prednisone on type I interferon signature in rheumatoid arthritis: consequences for response prediction to rituximab. *Arthritis Res Ther* 2015;**17**:78. doi:10.1186/s13075-015-0564-y

222 Muskardin TW, Vashisht P, Dorschner JM, *et al.* Increased pretreatment serum IFN-/ ratio predicts non-response to tumour necrosis factor inhibition in rheumatoid arthritis. *Ann Rheum Dis* 2016;**75**:1757–62. doi:10.1136/annrheumdis-2015-208001

223 Reed AM, Peterson E, Bilgic H, *et al.* Changes in novel biomarkers of disease activity in juvenile and adult dermatomyositis are sensitive biomarkers of disease course. *Arthritis Rheum* 2012;**64**:4078–86. doi:10.1002/art.34659

224 Reed AM, Crowson CS, Hein M, *et al.* Biologic predictors of clinical improvement in rituximab-treated refractory myositis. *BMC Musculoskelet Disord* 2015;**16**:257. doi:https://dx.doi.org/10.1186/s12891-015-0710-3

225 Quartuccio L, Mavragani CP, Nezos A, *et al.* Type I interferon signature may influence the effect of belimumab on immunoglobulin levels, including rheumatoid factor in Sjogren's syndrome. *Clin Exp Rheumatol* 2017;**35**:719–20.

226 Alexander T, Sarfert R, Klotsche J, *et al.* The proteasome inhibitior bortezomib depletes plasma cells and ameliorates clinical manifestations of refractory systemic lupus erythematosus. *Ann Rheum Dis* 2015;**74**:1474–8. doi:https://dx.doi.org/10.1136/annrheumdis-2014-206016

227 Petri M, Fu W, Ranger A, *et al.* Association between changes in gene signatures expression and disease activity among patients with systemic lupus erythematosus. *BMC Medical Genomics [Electronic Resource]* 2019;**12**:4. doi:https://dx.doi.org/10.1186/s12920-018-0468-1

228 Aranow C, Kamen DL, Dall'Era M, *et al.* Randomized, double-blind, placebo-controlled trial of the effect of vitamin D<inf>3</inf> on the interferon signature in patients with systemic lupus erythematosus. *Arthritis and Rheumatology* 2015;**67**:1848–57. doi:http://dx.doi.org/10.1002/art.39108

229 Hasni S, Gupta S, Davis M, *et al.* Safety and Tolerability of Omalizumab: A Randomized Clinical Trial of Humanized Anti-IgE Monoclonal Antibody in Systemic Lupus Erythematosus. *Arthritis & Rheumatology* 2019;**71**:1135–40. doi:10.1002/art.40828

230 McBride JM, Jiang J, Abbas AR, *et al.* Safety and pharmacodynamics of

rontalizumab in patients with systemic lupus erythematosus: Results of a phase I, placebo-controlled, double-blind, dose-escalation study. *Arthritis Rheum* 2012;**64**:3666–76. doi:10.1002/art.34632

231 Merrill JT, Wallace DJ, Petri M, *et al.* Safety profile and clinical activity of sifalimumab, a fully human anti-interferon alpha monoclonal antibody, in systemic lupus erythematosus: a phase I, multicentre, double-blind randomised study. *Ann Rheum Dis* 2011;**70**:1905–13. doi:https://dx.doi.org/10.1136/ard.2010.144485

232 von Wussow P, Jakschies D, Hartung K, *et al.* Presence of interferon and anti-interferon in patients with systemic lupus erythematosus. *Rheumatol Int* 1988;**8**:225–30. doi:10.1007/BF00269199

233 Quartier P, Allantaz F, Cimaz R, *et al.* A multicentre, randomised, double-blind, placebo-controlled trial with the interleukin-1 receptor antagonist anakinra in patients with systemic-onset juvenile idiopathic arthritis (ANAJIS trial). *Ann Rheum Dis* 2011;**70**:747–54. doi:http://dx.doi.org/10.1136/ard.2010.134254

234 Bienkowska J, Allaire N, Thai A, *et al.* Lymphotoxin-LIGHT pathway regulates the interferon signature in rheumatoid arthritis. *PLoS ONE [Electronic Resource]* 2014;**9**:e112545. doi:https://dx.doi.org/10.1371/journal.pone.0112545

235 López De Padilla CM, Crowson CS, Hein MS, *et al.* Interferon-regulated chemokine score associated with improvement in disease activity in refractory myositis patients treated with rituximab. *Clin Exp Rheumatol*;**33**:655–63.

236 Higgs BW, Zhu W, Morehouse C, *et al.* A phase 1b clinical trial evaluating sifalimumab, an anti-IFN-alpha monoclonal antibody, shows target neutralisation of a type I IFN signature in blood of dermatomyositis and polymyositis patients. *Ann Rheum Dis* 2014;**73**:256–62. doi:https://dx.doi.org/10.1136/annrheumdis-2012-202794

237 Dastmalchi M, Grundtman C, Alexanderson H, *et al.* A high incidence of disease flares in an open pilot study of infliximab in patients with refractory inflammatory myopathies. *Ann Rheum Dis* 2008;**67**:1670–7. doi:http://dx.doi.org/10.1136/ard.2007.077974
